# Genomic signatures and evolutionary history of the endangered blue-crowned laughingthrush and other *Garrulax* species

**DOI:** 10.1186/s12915-022-01390-4

**Published:** 2022-08-24

**Authors:** Hao Chen, Min Huang, Daoqiang Liu, Hongbo Tang, Sumei Zheng, Jing Ouyang, Hui Zhang, Luping Wang, Keyi Luo, Yuren Gao, Yongfei Wu, Yan Wu, Yanpeng Xiong, Tao Luo, Yuxuan Huang, Rui Xiong, Jun Ren, Jianhua Huang, Xueming Yan

**Affiliations:** 1 College of Life Science, Jiangxi Science & Technology Normal University, Nanchang, Jiangxi Province China; 2 College of Animal Science, South China Agricultural University, Guangzhou, Guangdong China; 3 Nanchang zoo, Nanchang, Jiangxi Province China

**Keywords:** Blue-crowned laughingthrush, Cooperative breeding, Demographic inference, Genetic diversity, Conservation strategies

## Abstract

**Background:**

The blue-crowned laughingthrush (*Garrulax courtoisi*) is a critically endangered songbird endemic to Wuyuan, China, with population of ~323 individuals. It has attracted widespread attention, but the lack of a published genome has limited research and species protection.

**Results:**

We report two laughingthrush genome assemblies and reveal the taxonomic status of laughingthrush species among 25 common avian species according to the comparative genomic analysis. The blue-crowned laughingthrush, black-throated laughingthrush, masked laughingthrush, white-browed laughingthrush, and rusty laughingthrush showed a close genetic relationship, and they diverged from a common ancestor between ~2.81 and 12.31 million years ago estimated by the population structure and divergence analysis using 66 whole-genome sequencing birds from eight laughingthrush species and one out group (*Cyanopica cyanus*). Population inference revealed that the laughingthrush species experienced a rapid population decline during the last ice age and a serious bottleneck caused by a cold wave during the Chinese Song Dynasty (960–1279 AD). The blue-crowned laughingthrush is still in a bottleneck, which may be the result of a cold wave together with human exploitation. Interestingly, the existing blue-crowned laughingthrush exhibits extremely rich genetic diversity compared to other laughingthrushes. These genetic characteristics and demographic inference patterns suggest a genetic heritage of population abundance in the blue-crowned laughingthrush. The results also suggest that fewer deleterious mutations in the blue-crowned laughingthrush genomes have allowed them to thrive even with a small population size. We believe that cooperative breeding behavior and a long reproduction period may enable the blue-crowned laughingthrush to maintain genetic diversity and avoid inbreeding depression. We identified 43 short tandem repeats that can be used as markers to identify the sex of the blue-crowned laughingthrush and aid in its genetic conservation.

**Conclusions:**

This study supplies the missing reference genome of laughingthrush, provides insight into the genetic variability, evolutionary potential, and molecular ecology of laughingthrush and provides a genomic resource for future research and conservation.

**Supplementary Information:**

The online version contains supplementary material available at 10.1186/s12915-022-01390-4.

## Background

Laughingthrush (*Garrulax*) is one of the most common genera (46 species) in the Timaliinae family and is widely distributed in Southeast Asia. Three quarters of the species are found in China [1]. Among them, the blue-crowned laughingthrush (*Garrulax courtoisi*, *GCO*) is endemic to China and only exists in Wuyuan, Jiangxi Province. It has a critically endangered (CR) status on the Redlist of China’s Biodiversity and was updated to the most critical class, I (Endangered rating, “I” means critically endangered), in the National Key Protective Species List [2], as well as on the International Union for Conservation of Nature (IUCN) Red List [3] (https://www.iucnredlist.org/).

**Fig. 1:**
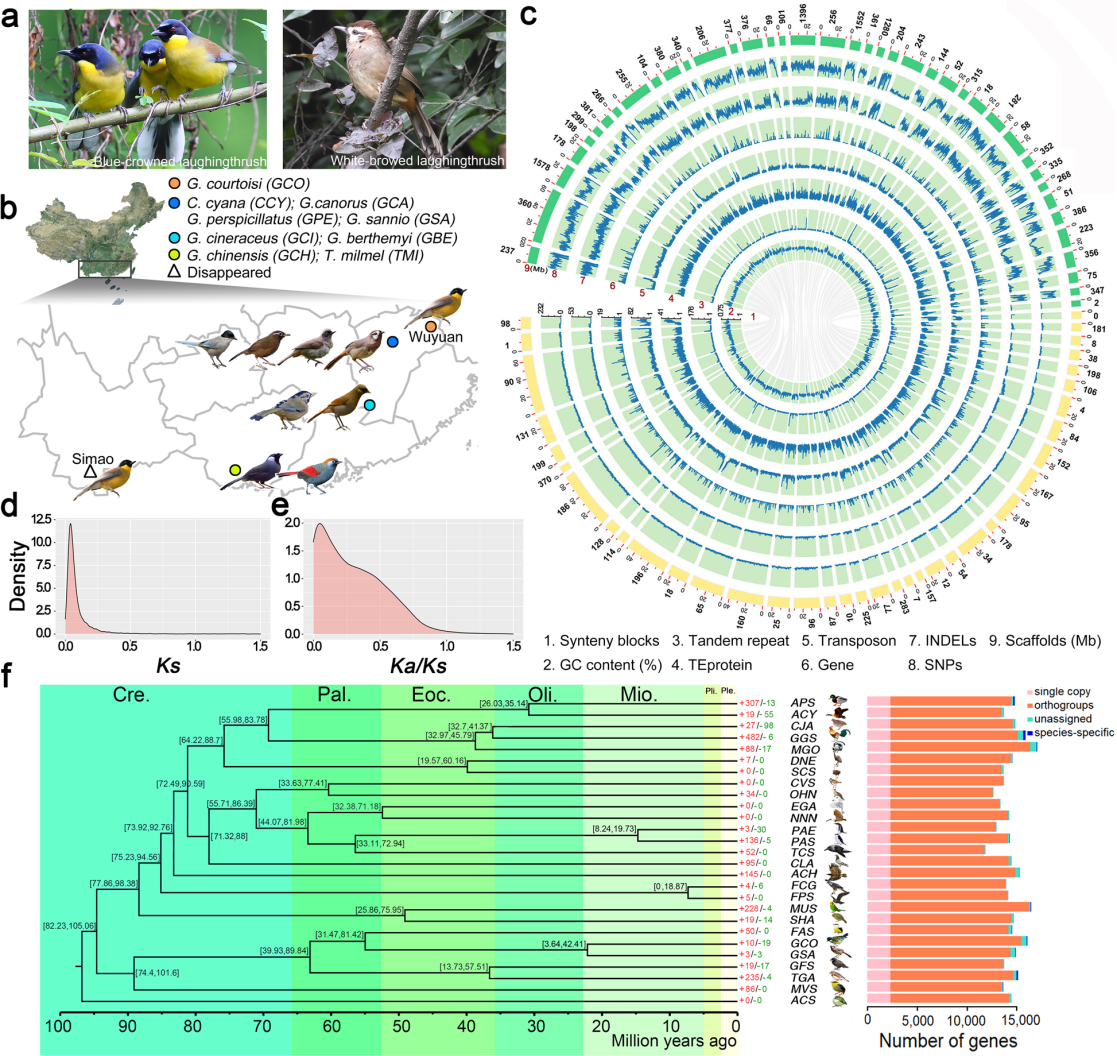
Comparative genomic analysis of the genome assemblies. **a** Image of *GCO* (left) and *GSA* (right). **b** Distribution of *GCO* in China. The magenta point represents current habitat of *GCO* in the Wuyuan area**.** The grayish blue indicates extinct *GCO* in Simiao area. **c** Genomic landscape of the *GCO* and *GSA*. The dark red numbers indicate the following: (1) Synteny blocks between the two species. (2) GC content (%). (3)–(8) Represent density of tandem repeats, TEprotein, Transposon, gene, INDELs, and SNPs within a 10-kb sliding window. (9) Assembled scaffolds, green for *GCO* and light yellow for *GSA.* The numbers outside the circle indicate the scaffold number of the assembled genome. The unit of (2) is the percentage, the scale of (3)–(8) is the frequency, and the unit (9) is Mb. The density distribution of *Ks* (**d**) and *Ka/Ks* (**e**) values. **f** A phylogentic tree was built on the 2391 shared single-copy gene families. *Acanthisitta chloris* (*ACS*) was the outgroup. The abbreviations of species correspond to Additional file 1: Table S13. The numerical value beside each node shows the estimated divergence time. The red and green numbers represent significantly expanded and contracted gene families, respectively (left). The classified homologous genes (right). Cre., Cretaceous; Pal., Paleocene; Eoc., Eocene; Oli., Oligocene; Mio., Miocene; Pli., Pliocene; Ple., Pleistocene

A mitochondrial-based phylogenetic relationship study revealed a close genetic relationship between *GCO* and white-browed laughingthrush (*Garrulax sannio*, *GSA*) [4]. *GCO* is similar in body size and reproductive habits to *GSA* but has a different appearance*.* The top of *GCO*’s head is dark blue with black sides and chin. During the breeding season, *GCO* is bright yellow from the throat to the abdomen, while *GSA* has brown feathers (Fig. 1a). *GCO* is a collective nesting and cooperative breeding bird living in old-growth trees and bushes near villages. As a cooperative breeding laughingthrush, *GCO* offspring have a communal breeding behavior in which offspring act as helpers. This means that the offspring are breeding helpers that stay in the family group and assist their parents in caring for raising the next generation of nestlings [4]. This breeding behavior can be compared to how older human children might help their parents take care of younger children.

For several hundred years, *GCO* was distributed in Simao, Yunnan Province, and Wuyuan, Jiangxi Province (Fig. 1b). The populations of *GCO* in both areas were apparently eliminated in the late nineteenth century, but some wild individuals in Wuyuan were rediscovered approximately 100 years later, which verified that *GCO* had not gone extinct in the wild [5–8]. In 2016, He et al. found ~323 wild living *GCO* in Wuyuan including ~200 fertile individuals. However, no wild individuals were found in Simao, indicating that *GCO* is extinct in Yunnan Province [9–11]. According to the observations of breeding behavior during artificial captivity and the literature records, *GCO* has lower reproduction ability than that of other laughingthrush [9]. Although the reproductive habits of *GCO* and *GSA* are similar, the survival rate of *GCO* nestlings is much lower than that of *GSA*. Considering the urgency of *GCO* protection, the National Nature Reserve was established around Wuyuan in 2016, and this measure has played an important role in recovery of the wild *GCO* population. In addition, an ex situ conservation of *GCO* has been initiated in the Nanchang Zoo.

Although *GCO* has received widespread attention, few studies have conducted a comprehensive analysis of their reproduction, environmental adaptation, and genomic features. Previous studies concluded that *GCO* has low genetic diversity [12], but this may not reflect the present status of *GCO* due to lack of a genomic sequence and limited sample size and variants. Given the current status of *GCO*, Li et al. have advocated for the rescue of China’s *GCO* [13]. However, the lack of a high-resolution reference genome of the *Garrulax* species, as well as difficult-to-obtain samples, has hindered research and species protection. As conservation genetic tools, detailed information on the genome assembly of *GCO* and other *Garrulax* species is desirable.

The assembly of reference genomes combined with whole-genome resequencing has effectively improved the protection of other endangered animals, such as the population size recovery of the crested ibis (*Nipponia Nippon*) [14, 15]. Exploring the genomes of different subspecies increases our understanding of the characteristics of endangered animals. For example, comparative genomic analysis revealed two different subspecies of giant pandas (*Ailuropoda melanoleuca*) and identified the loss of regulatory elements in the *DACH2* and *SYT6* genes that may be responsible for the reduced fertility of the giant panda [16]. Furthermore, the accumulation of genetic load in small populations may increase the risk of extinction via “mutational meltdown” [17]. Recent analyses of endangered species based on genomic data have found that the genetic load of small population with high inbreeding accelerates the accumulation of deleterious mutations [18–20]. The genome data of endangered species is a more accurate method to quantify genetic threats, such as decreased genetic diversity, increased inbreeding levels, and accumulated deleterious mutations [21, 22]. Since *GSA* and *GCO* share similar reproduction modes but differ in their endangered status and distribution, we assembled reference genomes of *GCO* and *GSA* and performed whole-genome resequencing of 66 birds to (1) investigate the genome characteristics, phylogenetic relationships, and ecological niche of laughingthrush; (2) determine the genetic diversity of laughingthrush; (3) understand the reasons for the CR status of *GCO*; and (4) provide a genetic basis and science-based advice for conservation of the *GCO*.

## Results

### De novo assemblies, validation, and annotation

We generated assemblies of the CR *GCO* and the least concern (LC) *GSA* using a multifaceted sequencing and assembling workflow, including PacBio reads, Illumina reads and 10× Genomics reads (Additional file 1: Method S1, Tables S1–S7 for details). The final assembled genomes totaled 1.22 Gb and 1.13 Gb for *GCO* and *GSA*, respectively, containing 1708 and 786 scaffolds with a contig/scaffold N50 size of 5.50/11.26 Mb and 8.31/22.71 Mb (Table 1 and Additional file 1: Tables S1–S4). These showed a similar genome size and large scaffold N50 compared with most avian genomes [23, 24]. Benchmarking Universal Single-Copy Orthologs (BUSCO) [25] covered 96.5% and 97.5% of 2586 conserved proteins, while the Core Eukaryotic Genes Mapping Approach (CEGMA) confirmed 210 and 214 of 248 complete core eukaryotic genes for *GCO* and *GSA*, respectively (Fig. 1c; Additional file 1: Table S5). These data indicate high completeness of the genomes of the two species. Repetitive sequences in *GCO* and *GSA* account for 18.74% and 16.55% of their genomes, respectively (Additional file 1: Figs. S1, S2 and Tables S6, S7). These values are smaller than the California condor [20] and chicken (galGal6/GRCg6a), and may reflect a slightly expanded assembled genome of the former (Fig. 1c). We predicted 20,819 and 18,367 protein-coding genes for *GCO* and *GSA* (Fig. 1c; Table 1 and Additional file 1: Table S8) with high confidence based on homology protein mapping and ab initio predictions. The distribution of gene features is consistent with six other homologous species, and the gene numbers averaged higher (23.6% and 13.4%) than these species (Additional file 1: Table S9). More than 90.9% of genes in the two genomes were functionally annotated and 74.6% of them were collectively annotated in SwissProt (http://www.uniprot.org/), KEGG (http://www.genome.jp/kegg/), and InterPro (https://www.ebi.ac.uk/interpro/) (Additional file 1: Fig. S3 and Table S10). Annotation of noncoding RNA of the two species revealed similar copy numbers for each genome (Additional file 1: Table S11). The two assemblies will provide the necessary basic conditions for subsequent research and protection for *Garrulax* species (Additional file 1: Method S1 and S2 for details).

**Table 1:** Summary of assembled genomes

Statistic	Blue-crowned laughingthrush (***Garrulax courtoisi***, ***GCO***)	White-browed laughingthrush (***Garrulax sannio***, ***GSA***)
Genome (Mb)	1120.72	1185.73
Total depth (×)	320.11	296.52
Total data (Gb)	359.43	351.6
Contig number	3080	1584
Contig N50 (bp)	5,499,984	8,312,805
Scaffold number	1708	786
Scaffold N50 (bp)	11,258,674	22,710,109
GC content (%)	43.25	42.59
Map rate (%)	98.24	97.35
Homology SNP (%)	0.0008	0.0003
CEGMA (%)	84.68	86.29
BUSCO (%)	96.50	97.50
Repeat elements (Mb)	228.68	187.39
Gene number	20,819	18,367

### Comparative genomic analysis

We identified 14,496 homologous genes accounting for 69.63% and 78.92% of the annotated genes of *GCO* and *GSA*, respectively, suggesting a high degree of conserved synteny between these two species. Unexpectedly, the variants of *GCO* were significantly more numerous than those of *GSA* (Fig. 1c). Some inversions and transpositions were also observed between long scaffolds in the two genomes (Additional file 1: Fig. S4). Non-synonymous mutation/synonymous mutation (*Ka/Ks*) analysis between *GCO* and *GSA* showed that 111 genes were positively selected (*Ka/Ks*>1, Fig. 1d, e) in *GCO*, of which 85 annotated genes (Additional file 1: Table S12) were mainly involved in the transcriptional regulation of blood circulation (*ASPH*, *C3AR1*, *CAV3*, *P2RY2*, *PTGER2*, *HCN4*, and *SMTNL1*) and adipose differentiation (*FABP3*, *PTGER2*, *PPARGC1A*, *APOC3*, and *CAV3*; Additional file 1: Fig. S5). We identified orthologous genes among *GCO*, *GSA*, and 25 other avian species covering ten bird orders (Additional file 1: Table S13). A total of 2319 1:1:1 single-copy orthologous genes and 4541 N:N:N orthologous genes were identified among them (Fig. 1f). We obtained 38 unique orthologous gene families, including 81 genes, of which *HXK2*, *MyH6*, *MyH7*, and *HKDC1* are involved in the ATP metabolic process. In *GSA*, there are 18 unique orthologous gene families, including 60 genes, of which, *PAK3*, *SCN2A*, and *NAE1* are involved in the neuron apoptotic process. The lineages to which the two species belong were estimated and found to have diverged from the collared flycatcher (*Ficedula albicollis*) ~31.47–81.42 Mya. *GCO* experienced the largest gene family expansion with 1698 expanded and 1114 contracted gene families, while *GSA* has 540 expanded and 890 contracted gene families (Additional file 1: Table S14). There are 29 genes with significant gain and loss in *GCO*, but only six in *GSA* (Fig. 1f).

### Population structure of Garrulax species

**Fig. 2:**
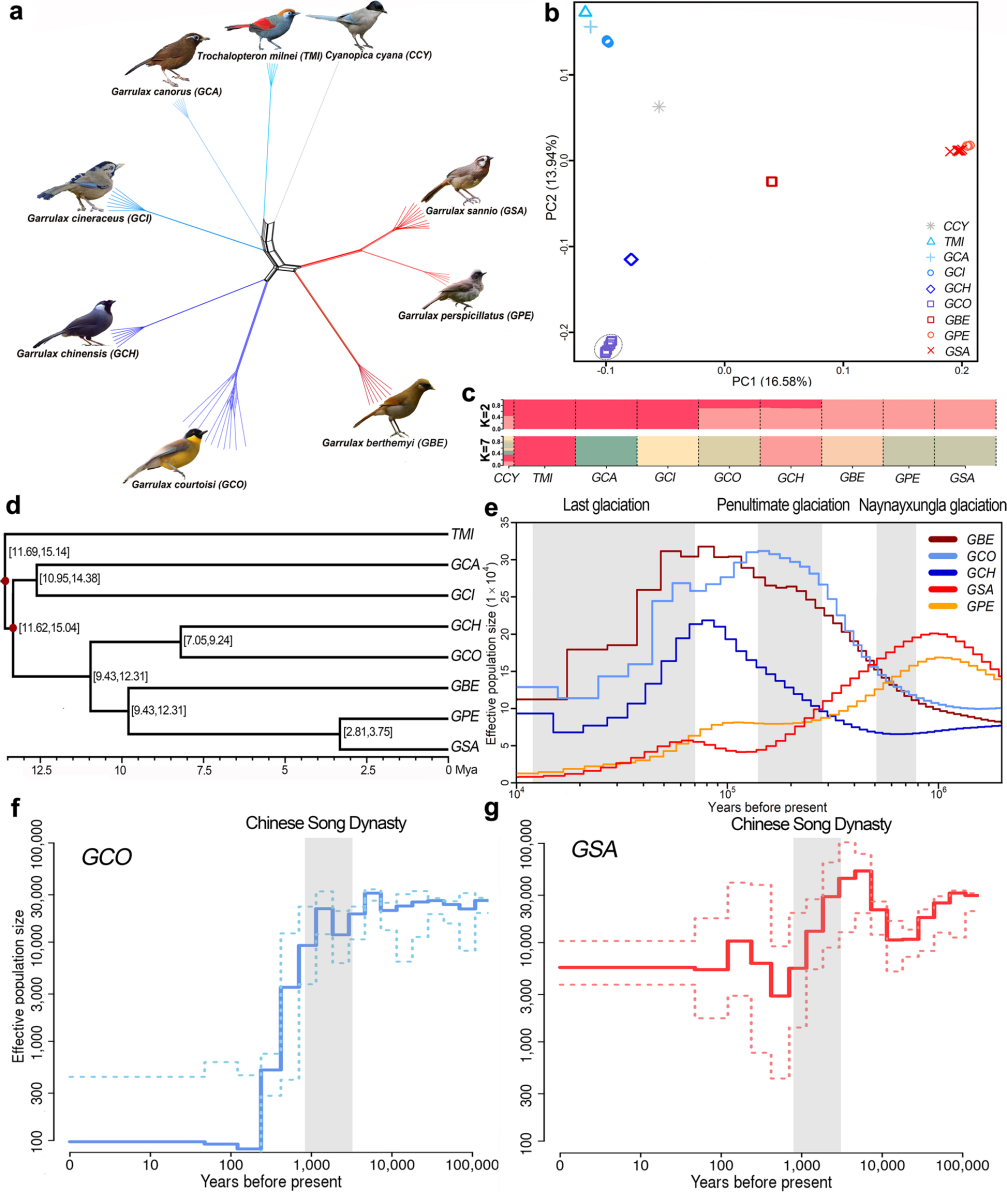
Phylogenetic relationship population inference. **a** Neighbor-joining (NJ) phylogenetic tree of the nine species with 58 individuals. **b** Principal component analysis of 58 individuals with the first and second principal components being visualized. Color codes for different species are as in **a**. **c** Ancestry of *Garrulax* species. K represents assumptive ancestral lineage. Each vertical line represents one species, and each color represents one ancestral lineage. The length of each colored segment in each vertical indicates proportion of individual genome inferred from ancestral populations. Species are separated by black dotted lines. Abbreviations of species are given in Additional file 1: Table S15. **d** Estimation of the divergence time among eight *Garrulax* species. Values in square brackets indicate the divergence time ranges. Dark red nodes represent time calibration points. **e** Demographic inference based on the Pairwise Sequentially Markovian Coalescent (PSMC) model. The five-colored lines depict the estimated effective population size of the five species dating to roughly 10,000 years ago. The gray background colors indicate glacial periods. **f, g** showed effective population size in the last 10,000 years for *GCO* and *GSA* inferred via PopSizeABC. A 90% confidence interval is indicated by dotted lines. The gray shaded area represents the Song Dynasty period from 960 to 1279 AD

We sequenced 66 birds including eight *Garrulax* species and a *Cyanopica cyanus* with an average coverage depth of ~21.41× (16.62–27.89×; Fig. 1b and Tables S15, S16). After excluding potential related individuals (Additional file 1: Fig. S6 and Table S17), 58 birds were clearly located at nine independent branches, which reflected distinct genetic differentiation among them (Fig. 2a; Additional file 1: Figs. S7, S8). In comparison, *GCO* has the closest genetic relationship with the black-throated laughingthrush (*Garrulax chinensis*, *GCH*), while *GSA* is close to the masked laughingthrush (*Garrulax perspicillatus*, *GPE*). This was simultaneously revealed by principal component analysis (PCA), maximum-likelihood (ML) phylogenies, and the ancestor consanguinity component (Fig. 2b; Additional file 1: Fig. S9). The red-tailed laughingthrush (*Trochalopteron milnei*, *TMI*), Chinese Hwamei (*Garrulax canorus*, *GCA*), and mustached laughingthrush (*Garrulax cineraceus*, *GCI*) are composed of the same ancestral lineages. The rusty laughingthrush (*Garrulax berthemyi*, *GBE*), *GPE*, and *GSA* comprised another lineage, while *GCO* and *GCH* showed a mixed lineage (Fig. 2c). Unexpectedly, when *K* = 7 (optimal *K* value; the minimum CV-error; Fig. 2c; Additional file 1: Figs. S9, S10), there was almost no separation of the ancestral lineage between *GSA* and *GPE*. The phylogenetic relationship of the eight *Garrulax* species based on single-nucleotide polymorphisms (SNP) further showed the relatively close genetic relationship associated with a more recent divergence time (~2.81–3.75 Mya; Fig. 2d; Additional file 1: Fig. S11).

### Gene flow among Garrulax species

We considered the large amount of similarities and overlaps in the food and habitats of *Garrulax* species, which may reflect potential gene flow among them. We confirmed the existence of some migration events among *Garrulax* using Treemix when the migration events ranged from one to five. Interestingly, *GCO* and *GSA*/*GPE* showed highest migration weight, which indicates the existence of strong gene flow among them. The D-statistics also detected evidence of gene flow between *GCO* and *GSA/GPE*, and between *GCO* and *GCA*/*GBE* (Additional file 1: Fig. S12, Table S18a, b). In addition, *GCI* showed weak gene flow with *GPE*, *GCO*, and *GSA*. We also verified gene flow among other *Garrulax* species, especially between *GCH* and *GSA*/*GBE* (Additional file 1: Table S18c). These results reflect a common phenomenon: various degrees of gene flow exist among closely related species in some genera, such as the *Bos* genus [26].

### Demographic history in Garrulax species

Admixture and ABBA-BABA analysis showed that *GCH*, *GCO*, *GBE*, *GPE*, and *GSA* have a close genetic relationship and frequent gene flow. Therefore, we estimated the historical effective population size of these five species over the past 10,000–1 Mya. We found two different ancient demographic fluctuation patterns, *GSA* and *GPE* showed a continuous decline during penultimate glacial and the last glacial period, while *GCO*, *GCH*, and *GBE* showed another similar fluctuation pattern, in which the effective population size increased slowly during the penultimate glacial period and declined rapidly during the last glacial period (Fig. 2e). We found that the effective population size of *GCO* was higher than that of other *Garrulax* species until 10,000 years ago. The *GCO* population has decreased dramatically in the last 1000 years (Fig. 2f). Notably, the demographic pattern showed that *GCO* was almost extinct during the past ~200 years, which is consistent with past survey results [9], and this result was also supported by smc++ analysis (Additional file 1: Fig. S13). However, the *GSA* population also experienced a sharp decline, and then a rapid increase, over the last 1000 years (Fig. 2g).

In contrast to its current endangered status, *GCO* had a large effective population size before 10,000 years ago that was higher than other *Garrulax* species (Fig. 2e, Additional file 1: Fig. S14). However, both *GCO* and *GSA* fluctuated significantly during the most recent 10,000 years (Fig. 2f, g), and the two species sharply declined during the Chinese Song Dynasty (960–1279 AD). Subsequently, *GSA* gradually recovered to previous levels, whereas *GCO* maintained a small population size. This situation may be caused by low fecundity, a longer generation interval, or frequent human activities within the habitat of *GCO*. Monitoring data showed similar reproductive performance between *GCO* and *GSA*, including similar clutch size (2–4 vs. 3–4 eggs) and incubation period (12–14 vs. 15–17 days). However, the ratio of successful nesting appeared to be significantly different between *GCO* and *GSA*. *GSA* and *GCO* averaged 73.3% and 25% a year, respectively. In some years, we did not observe successful maturation of offspring in the *GCO* population throughout the year, which most likely resulted from frequent human disturbance [9, 27, 28]. The survival rate of juvenile *GCO* is low [27], but they exhibit cooperative breeding behavior that helps increase the survival rate of nestlings.

### Abundant genetic diversity in endangered blue-crowned laughingthrush

**Fig. 3:**
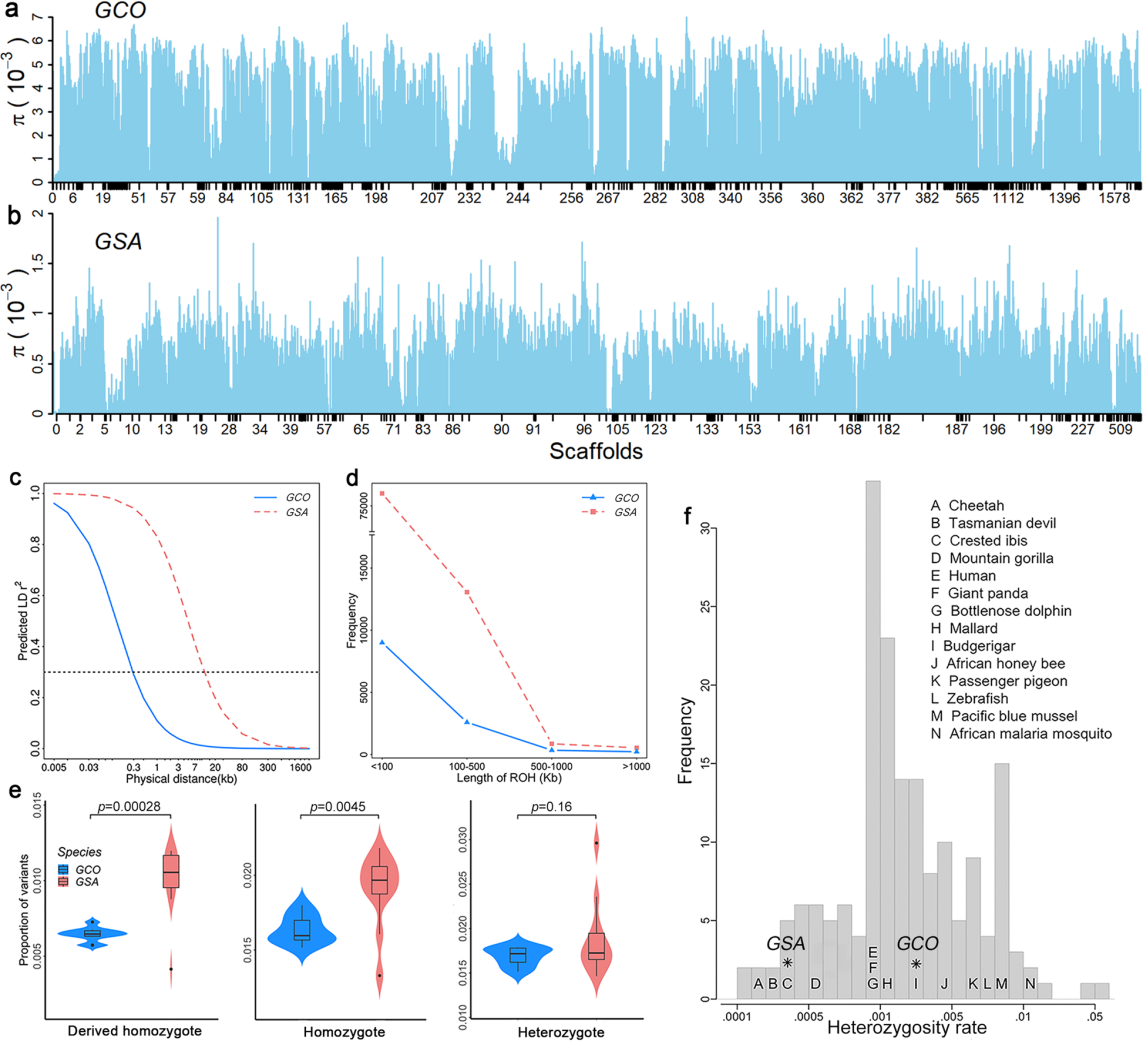
Genetic diversity analysis. Nucleotide diversity estimated based on the respective reference genomes of *GCO* (**a**) and *GSA* (**b**). **c** The LD decay of *GCO* and *GSA*. The black dotted line indicates the mean length of genome segments when *r*^2^=0.3. **d** The ROH of *GCO* and *GSA*. The *x*-axis denotes four lengths of ROH segments: <100 kb, 100–500 kb, 500–1000 kb, and >1000 kb. The *y*-axis denotes the number of different ROH segments in their categories. **e** The proportion of deleterious mutations in *GCO* and *GSA*. **f** Histogram distribution of published genome-wide estimates of 186 species (167 animals, 15 plants, 3 fungi, and 1 protist taxa), with the position of *GCO* and *GSA* heterozygosity rates indicated by asterisks

Because of their small population size, threatened and endangered species often suffer from a lack of genetic diversity, potentially leading to inbreeding depression and reduced adaptability [29]. Previous studies have found significant differences in the genetic diversity between *GCO* and *GSA* [14, 30]. To confirm whether the genetic diversity of *GSA* is higher than that of *GCO*, we calculated nucleotide diversity (*π*) of the two species using SNPs detected from their respective reference genomes. Unexpectedly, *GCO* showed a significantly higher *π* than that of *GSA* at population (3.79×10^−5^ vs. 0.16×10^−5^) and individual (2.7×10^−3^ vs. 0.3×10^−3^; *t*-test *P* = 5.67×10^−6^) levels, respectively (Fig. 3a, b; Additional file 1: Figs. S15–S17). We also found long runs of homozygosity (LROH, *π* < 1.0×10^−4^) in the genomes of the two species, and the homozygous regions of *GSA* seem to be longer than those of *GCO* (Additional file 1: Figs. S16 and S17, *P* = 9.93×10^−7^). *GCO* showed shorter linkage disequilibrium (LD) extension (Fig. 3c) and run of homozygosity (ROH) lengths in different fragments (Fig. 3d), which further indicated a higher degree of inbreeding in *GSA* than in *GCO*. We also detected the genetic diversity among eight *Garrulax* species using the SNPs based on *GSA* reference. *GCO* had the richest genetic diversity, while the other seven *Garrulax* species showed very low genetic diversity (Additional file 1: Table S19). In comparison to other *Garrulax* species, *GCO* had the smallest inbreeding coefficient (−0.22 vs. −0.08; *P =* 8.5×10^−3^; Additional file 1: Fig. S18), highest *π* (1.56×10^−4^ vs. 4.78×10^−5^; *P* = 3.7×10^−5^; Additional file 1: Figs. S19–S21), shortest LD extension, and fewest ROH fragments (Additional file 1: Fig. S22) among the eight *Garrulax* species. However, *GSA* had the highest inbreeding levels (Additional file 1: Fig. S23).

Small populations are more likely to accumulate strong deleterious variants [29]. We predicted the degree of variants, and *GCO* showed a significantly lower proportion of homozygous strong deleterious variants than *GSA* (*P* < 0.004), whereas there was no difference in heterozygous variants (Fig. 3e). In contrast, *GCO* had a significantly higher proportion of slightly deleterious variants than *GSA* (*P* < 9.2×10^−4^; Additional file 1: Fig. S24 and Table S20). To further explore the endangered status of the two species among other biological species, we compared *GCO* and *GSA* to 186 species globally (Additional file 2: Table S21), including mammals, birds, marine organisms, invertebrates, and plants confronted with different degrees of threat. We found that the nucleotide diversity of *GCO* is higher than most LC species, while *GSA* is close to the CR crested ibis (*Nipponia nippon*), which is contrary to normal perception (Fig. 3f). These data suggest that the genomic status of *GCO* does not correspond to its endangered survival status.

### Obvious selective sweeps occurred in the endangered blue-crowned laughingthrush

**Fig. 4:**
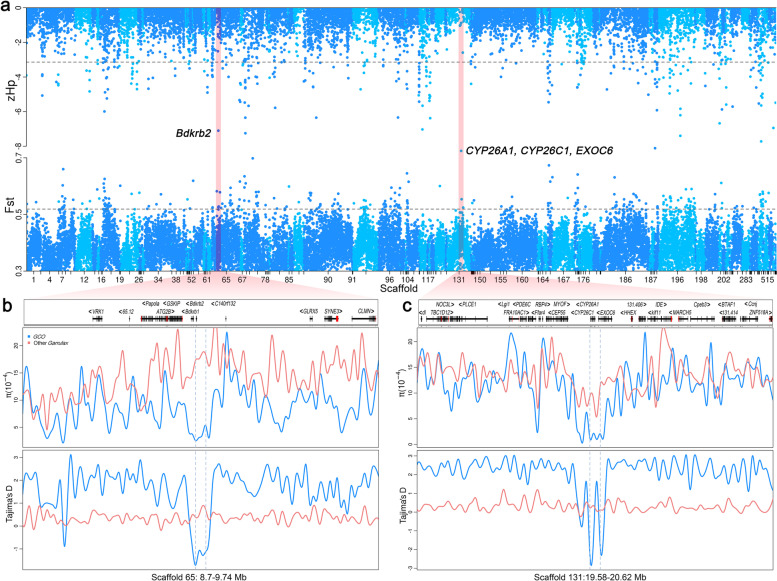
Selective sweeps of *GCO*. **a** The Manhattan plots of Z-transformed average pooled heterozygosity in *GCO* (zHp), and average fixation index (*F*_ST_) between *GCO* and seven other *Garrulax* species. The shadows identified by *F*_ST_ and zHp represent the regions with the extremum of the two statistics. Nucleotide diversity and Tajima’s *D* values with non-overlapping 10-kb sliding windows on Scaffold 65: 8.7–9.74 Mb (**b**) and Scaffold 131: 19.58–20.62 Mb (**c**)

In consideration of the critically endangered status of *GCO* and its cooperative breeding behavior, we hypothesized that the *GCO* genome may retain footprints reflecting these characteristics. Hence, we conducted *F*_ST_ analysis between *GCO* and other laughingthrush species. After long-term natural selection, homozygous regions related to the above characteristics may remain in the *GCO* genome. Therefore, we carried out zHp analysis within the *GCO* population. Overlap windows of zHp and *F*_ST_ were used to explore the putative selective sweeps in *GCO* (Fig. 4 and Additional file 1: Fig. S25). Taking experiential top 1% (*F*_ST_ = 0.52 and zHp = −3.14) as selective sweeps thresholds, we identified 42 overlapping windows, of which, two windows including regions of 9.2–9.24 Mb on Scaffold 65 (*F*_ST_ = 0.53, zHp = −7.10) and 20.08–20.12 Mb on Scaffold 131 (*F*_ST_ = 0.55, zHp = −8.27) had the lowest zHp values. A total of 22 overlapping genes were identified from the 42 windows (Fig. 4a; Additional file 1: Table S22), of which, two windows including *Bdkrb2* and *CYP26A1* with the lowest zHp and highest *F*_ST_ were selected because they had lower *π* and Tajima’s *D* values with an obviously selective sweep in *GCO* (Fig. 4b, c).

### Sex confirmation and marker-assisted breeding based on the short tandem repeat profiling of blue-crowned laughingthrush

Using genomic loci as trace to reduce inbreeding in endangered species is often used in the protection and rescue of endangered animals, as well as for sex identification [31]. In contrast to sexually dimorphic birds, the sexes of *GCO* are difficult to separate. We used a depth-based strategy to identify scaffolds representing the sex chromosomes of *GCO* and *GSA*. In *GCO*, 15 and 10 scaffolds were identified as Z- and W-related chromosomes (Additional file 1: Table S23). For *GSA*, 24 and five scaffolds were regarded as Z- and W-related chromosomes, respectively (Additional file 1: Table S24). We then checked the sex of all *GCO* and *GSA* individuals and found four females and ten males in *GCO*, and four females and seven males in *GSA*, which was identical to sample records (Additional file 2: Tables S23, S24).

Due to the abundant number of short tandem repeats (STR) and a high degree of variants among individuals, we detected a genome-wide STRs for *GCO*. We identified 233,287 STRs in the whole-genome of *GCO*, which the minor STR alleles exhibited a 17.35-bp difference from their major alleles. We then focused on the 1021 STRs with characteristics of a 4-bp repeat unit, allele types greater than four, and non-missing in 14 *GCO* individuals. Among these, 218 STRs with abundant polymorphism information content (PIC) were located in the 32-annotated-gene region (Additional file 1: Table S25) and can be used as identification of kinship in *GCO*. Besides, 1688 of 3941 STRs on the W-related scaffolds were identified with characteristics of homozygote in females and deletion in males, 43 of them were surrounded by 12 genes, which can be used as sex identification markers (Additional file 1: Table S26).

## Discussion

The assembled genomes of *GCO* in CR state and *GSA* in LC state reported here provide insights into the evolutionary history of *Garrulax* species and a basis for future genetic and genome-wide studies, not only in the laughingthrush but also in other songbirds. These genomes are typical for avian species: total length, synteny, repeat burden, and GC content (Fig. 1c; Table 1). They are similar to those of previously sequenced bird genomes [32], even those of distant relatives [33]. Here, we noticed that the variants (SNPs and INDELs) in the *GCO* genome are much higher than those of *GSA* (Fig. 1c), which suggests an abundant demographic history of *GCO* before they became endangered. A high level of historical abundance was also found in the California condor [20]. This conclusion is consistent with demographic inferences based on several methods for eight *Garrulax* species, which showed that *GCO* maintained the highest effective population size before the last glaciation and kept a dramatically small number of wild populations after the Chinese Song Dynasty (960–1279 AD). Coincidentally, China experienced a period containing several cold intervals during the Song Dynasty [34, 35], which may have decreased avian population sizes because of reduced food supplies or poor adaptation to the cold conditions. The bottleneck events occurred almost simultaneously in the *Garrulax* species and remind us that there may have been parallel evolution among laughingthrush species due to their similar body size, biology, and overlapping habitats. Parallel evolution is common in birds, such as ptilopody in chickens and pigeons [36]. In addition to their similar dietary habits, *GCO* and *GSA* also show similar reproductive habits, like cooperative breeding. The economy and culture flourished during the Chinese Song Dynasty, which stimulated the prevalence of pet aviculture. One aviculture expression, called “Liu Niao,” translates to the behavior of people admiring pet birds in cages. Evidence for this is provided in the form of delicate birdcages and realistic bird paintings [37, 38], as well as in the excavation of cultural relics and research findings on the characteristics and trade of the thrush [39, 40]. In addition, with a colder climate and humans migrating southward, the habitats of *GCO* were likely affected, and some suitable habitats were probably destroyed [41]. Although laughingthrush were continuously captured and kept as pets [40], their population size recovered after suffering from a short-term bottleneck, which may be attributed to rising temperatures and strong reproductive performance, such as *GCA* and *GSA* [42]. However, since *GCO* persistently maintains a small population size, another reason may be human overhunting due to their desirable ornamental appearance compared to other laughingthrush. More than 400 *GCO* were poached from 1987 to 1992 and trafficked to various countries [43]. Trapping may be one of the major reasons for the disappearance of *GCO* in the Simao area due to their sensitivity to the environment and human activities. To prevent the extinction of *GCO* in Wuyuan, protecting them and their breeding habitats requires the immediate and coordinated action of the government and the public [13]. Fortunately, the Wuyuan National Nature Reserve has been established as a protected area to provide a better habitat for *GCO* and other CR species.

Small populations are more likely to experience genetic drift, accumulation of deleterious mutations, and reduced genetic diversity. They are especially vulnerable to extinction due to deterministic and stochastic threats [29]. For a species that was nearly extinct in the wild, inconsistent with its CR status, the *GCO* has unexpectedly higher genome-wide diversity than other laughingthrush. The geographical distance between Simao, a previous habitat of *GCO*, and the existing habitat of Wuyuan (more than 2300 km away), the migratory ability of *GCO* [4, 9, 27], and the results of rich genetic diversity suggest that *GCO* was once widely distributed in Southern China, and there may have been substantial gene exchange among different groups. Both a sustained bottleneck and a strong, yet brief, bottleneck can lead to the reduction of genetic diversity [44, 45]. After experiencing a long-term bottleneck, *GCO* still has substantial genetic diversity compared to the LC *Garrulax* species, which suggests that *GCO* had a large population size before it became endangered. In addition, the demographic inference results showed that *GCO* was abundant about 1000 years ago, which also supports the conclusion that *GCO* was once widely distributed in Southern China. In contrast, other laughingthrush birds lack migratory ability [42] (https://species.sciencereading.cn/). This phenomenon means a lack of genetic exchange among different groups that may lead to a decline in genetic diversity, even in laughingthrush birds widely distributed in China. These facts may help explain why the genetic diversity of *GCO* is significantly higher than that of other laughingthrushes, and also suggests that the effective population size of *GCO* was large until 1000 years ago. Decreased selection intensity and increased genetic drift can be caused by several generations of inbreeding in a small population, and result in a lower *π*, higher LD decay, longer ROHs, and more deleterious mutations [46]. However, our results support the opposite conclusion, which is similar to results on the California condor (*Gymnogyps californianus*) [20] and the island fox (*Urocyon littoralis*) [30]. The endangered *GCO* showed the smallest inbreeding coefficient, highest *π*, shortest LD extension, and fewest ROH fragments among eight *Garrulax* species. Compared to most other LC species, *GCO* have a higher heterozygosity rate. Such anomalies may be attributed to the communal breeding behavior in which *GCO* offspring act as helpers [4], which effectively avoids inbreeding. According to the field tracking observations and breeding experience with captive wildlife in Nanchang and Hong Kong, the lifespan of *GCO* is about 20–30 years, while other *Garrulax* species, such as *GCA* and *GSA*, have an average lifespan of 8–15 years. The longer generation times of *GCO* might slow the loss of genetic diversity. The length of time needed to reach maturity and the cooperative pattern of reproduction are important questions that would benefit from additional research.

Consistent with the genetics of kākāpō and island fox [30, 47], we found *GCO* accumulates more slightly deleterious variants and fewer strongly deleterious variants compared to *GSA*. Species in the CR state for a long time, such as *GCO* and island fox, that permanently maintain small population sizes tend to accumulate fewer strongly deleterious variants, and this reduces the risk of inbreeding depression [30]. For these species, longer generation times and reduced inbreeding will persistently eliminate strongly deleterious mutations. Our research showed that the critically endangered *GCO* currently fits this survival model.

Since *GCO* has the characteristics of being extremely endangered with a long lifespan, cooperative reproduction, and poor environmental adaptability, it is possible that these characteristics may leave genetic footprints on the *GCO* genome. Human tourism and hunting activities have an impact on *GCO*’s nesting and reproduction, and cause behaviors such as nest abandonment [48]. Multiple genes were selected in *GCO* after long-term natural selection, which are well-characterized in humans but not in birds. For example, previous studies showed that the *Bdkrb2* gene is related to human anxiety [49], which suggests an association between *Bdkrb2* and the sensitivity of *GCO*. According to field observations, *GCO* is very alert and sensitive and maintains a relatively long distance from humans, even when living in old-growth trees and bushes near villages that often have high levels of human activity. It is difficult to view *GCO* at close range or to observe them at any distance. As a famous caged *Garrulax* [40] species, *GCO* has a beautiful birdsong and bright feathers which has made it a desirable target for human capture, especially during the Song Dynasty. Consequently, humans capturing them and natural selection may have long subjected *GCO* to anxiety behavior. We detected a strong selected gene, *CYO26A1*, which is related to reproductive performance. The activity of *CYP26A1* is important for maintaining pregnancy, and it may affect embryo implantation by regulating the differentiation and maturation of uterine dendritic cells. Thus, inhibiting the expression of *CYP26A1* will lead to pregnancy failure [50]. To ensure the birth and survival of nestlings, the competitive behavior among adult birds during cooperative breeding is significantly lower than that during the non-breeding period [4], which may be related to the *CYP26A1* gene in *GCO*. These genes are understood in humans, but it is unclear what the functions of these genes are in birds; thus, studies on model species such as zebra finches may reveal more about the roles of these genes.

Genotyping is a common way of identifying the sex of non-dimorphic birds. We identified scaffolds representing sex chromosomes and selected 43 sex-linked STRs to identify the sex of the *GCO* samples. These will be used to reconstruct the pedigree relationships of wild *GCO*. Then, we will establish individual identities to avoid losing genetic diversity and assist in increasing the *GCO* population size. This strategy has been successful in increasing the population size of crested ibis [14, 15]. However, unlike large size birds (such as crested ibis), it is difficult to apply to *GCO* without injuring the birds. The most effective protection measure at present is to establish a national nature reserve for *GCO* habitat and reduce human tourism activities. In addition, the narrow geographical distribution of *GCO* indicates its extreme sensitivity to, and dependency on, preferred breeding habitats. Wuyuan is advocating bird-watching and all-for-one tourism (in which tourism is taken as the dominant industry to driving and promoting the local economy), which provides both opportunities and challenges for the protection of *GCO*. It is important to consider the natural habitats, the lifestyles of local people, and the cultural environment of Wuyuan as a whole for protecting the CR blue-crowed laughingthrush.

## Conclusions

In summary, we provided two high-quality genomes of *GCO* and *GSA* and constructed their phylogenetic relationship, which will facilitate the researches about their conservation and diversity. Indeed, we revealed the genetic relationship between eight *Garrulax* species based on genome-wide sequencing data. The demographic histories showed the critically endangered *GCO* experienced a serious bottleneck event in the recent 1000 years. We considered that the sharply decreased effective population size of *GCO* may be caused by the lack of food, human activities, and poor environment adaptability. In addition, *GCO* exhibits a genetic heritage of population abundance, suggesting that fewer deleterious mutations can be stably inherited in small populations without causing lethal damage to the survival. Cooperative breeding behavior and a long reproduction period may play a vital role in this process. We identified 43 STRs that will aid in pedigree construction and avoid inbreeding depression of *GCO*. Our research provides a genome-level perspective for the genetic conservation of critically endangered *GCO*.

## Methods

### Samples

All procedures used for this study that involved birds complied with guidelines for the care and utility of experimental animals established by the Ministry of Agriculture of China and the Jiangxi Forestry Bureau (permission number: 2006-398). The ethics committee of Jiangxi Science & Technology Normal University approved this study. The samples used in this study were collected from different areas, among which *GCO* was a captive population raised in the Nanchang Zoo with permission from relevant authorities. The 14 *GCO* individuals were captured at different locations in Wuyuan during 2014–2016 for the purpose of ex situ conservation. We collected blood samples from the wing veins of 66 birds, including 14 *GCO*, six *GCA*, six *GCH*, five *GPE*, 11 *GSA*, eight *GBE*, seven *GCI*, eight *TMI*, and one outgroup of azure-winged magpie (*Cyanopica cyana*, *CCY*). Genomic DNA was extracted using a Whole Blood DNA Extraction Kit (Thermo Fisher Scientific, Waltham, MA, USA). DNA integrity was verified with agarose gel electrophoresis. Two adult females of *GCO* and *GSA* were selected for de novo assembly. The liver and muscle tissues were collected from individuals suffering accidental death and mixed to extract RNA using Invitrogen TRIzol (Thermo Fisher Scientific) for subsequent gene functions annotation.

### Genome assemblies

De novo assembly for *GCO* and *GSA* used third-generation single-molecule real-time sequencing (PacBio SMRT), second-generation sequencing platform (Illumina NovaSeq 6000) [51], and 10× Genomics (https://www.10xgenomics.com/) Linked-reads technology (total sequencing depths were approximately 320× and 297×). To estimate the genome size of the two genomes, Illumina short reads were recruited to determine the K-mer distribution. According to the K-mer-based estimation theory [52], the size of the haploid genome of *GCO* was estimated to be 1120.72 Mb and the haploid genome of *GSA* was estimated to be 1185.73 Mb. The qualified genomic DNA was fragmented by 26 G Needle, and PacBio SMRTbell (https://www.pacb.com/technology/hifi-sequencing/sequel-system/) libraries (20 kb insert) were prepared for single-molecule real-time sequencing with sequencing depths more than 95× and raw data larger than 113 Gb. Based on the continuous long read (CLR) generated by the PacBio platform, wtdbg2 [53] was used to directly perform de novo assembly. The draft genome scaffolds were produced after alignment, assembly, and polishing. Then, the linked-reads (99.10×) generated by the 10× GemCode platform were assembled with the consensus sequences to acquire a super-scaffold version of the two genomes. The assembled version was polished iteratively two to three times to improve the single-base correction rate using Pilon v1.23 [54] based on short reads (134.10× and 113.17 Gb) generated by the Illumina platform (Additional file 1: Method S1).

### Validation of genome assemblies

To evaluate the accuracy and integrity of genome assemblies, we used BWA v 0.7.17 [55] to re-align the Illumina short reads to the two assembled *Garrulax* genomes and counted the mapping rate, coverage, and sequencing depth. The mapping rate and coverage rate of two genomes were > 97.3%, indicating that the high-quality short reads and the assembled genomes had good consistency. In addition, SNP sets of two *GCO* and *GSA* were obtained based on the mapped reads used by SAMtools v1.10 [56]. The homozygous SNP ratios were less than 0.0008%, which reflected the accuracy of genome assemblies. Completeness of the two genomes were assessed based on 248 core genes in the Core Eukaryotic Genes Mapping Approach (CEGMA) program [57] and 2586 conserved genes in the BUSCO program [58] with default parameters. CEGMA assessment combined with tblastn v2.2.26 [59], genewise v2.4.1 [60], and geneid v1.4.5 software (https://genome.crg.es/software/geneid/) to evaluate the completeness of assembled genomes. BUSCO assessment based on tblastn, Augustus v3.3.3 [61], and hmmer [62] to assess the integrity of assembled genomes. Results showed that over 84% of 248 core genes and over 97% of 2586 avian orthologous genes were found in the *Garrulax* genomes, also reflecting its high quality. In addition, the Program to Assemble Spliced Alignments (PASA) tool was used to assemble transcripts based on RNA-seq reads that were aligned to the two *Garrulax* genome assemblies.

### Repeat elements

Repeat elements occupy a major proportion of the nuclear DNA in most eukaryotic genomes. Repeat sequence annotation methods were divided into two types: homologous sequence alignment and de novo prediction (Additional file 1: Method S2). We first customized a de novo repeat library of the genome using RepeatModeler, which can automatically execute two de novo repeat finding programs. The remaining consensus sequences were used as the library in RepeatMasker [63] to identify and cluster repetitive elements. The Tandem Repeats Finder (TRF) package v4.09 [64] was used to identify tandem repeat sequences in the *Garrulax* genomes.

### Gene prediction and functional annotation

After the masking of repeats, we used a combination of homology-based prediction, de novo prediction, and RNA-Seq-assisted prediction to predict structural annotation of the *Garrulax* genomes (Additional file 1: Method S2). De novo prediction was performed using Augustus, GlimmerHMM v3.0.4 [65], and SNAP v2013.11.29 [66]. For homologous annotation, the *Garrulax* genome sequences were compared to the protein-coding sequences of homologous species (*Struthio camelus*, *Nipponia nippon*, *Anas platyrhynchos*, *Gallus gallus*, *Meleagris gallopavo*, and *Anser cygnoides*) by BLAST [67] and genewise [60] to predict the gene structure. The transcript isoforms of *Garrulax* were mapped to the genome and then assembled by PASA v2.4.1 [68]. EVidenceModeler v1.1.1 (EVM; http://evidencemodeler.sourceforge.net/) was used to integrate these gene models from the above methods. The predicted protein sequences were compared with several public databases (SwissProt, NR, Pfam, KEGG, and InterPro) to further detect the function of the protein-coding genes in *Garrulax*.

### Comparative genome analysis

The genome synteny between *GCO* and *GSA* was evaluated by the One step MCScanX method from TBtools and visualized through the Advanced Circos method [69]. *Ks* values between the two species were calculated using Simple *Ka/Ks* Calculator method in TBtools. Protein and CDS sequences of the other 25 species were downloaded from NCBI (Additional file 1: Table S13). The 25 species selected cover the main families of birds to show the taxonomic position of *Garrulax* species in birds. OrthoFinder v2.4.1 [70] was used to analyze single-copy homologous gene families of the 27 bird species after extracting the longest transcripts and removing the transcripts if their length was less than 150 bp. The command -m MFP -bb 1000 -bnni -redo from IQ-tree v2.0.6 [71] was used to construct a species phylogenetic tree based on single-copy homologous gene families after aligning by MUSCLE v3.8.1551 [72] and BMGE [73] software. The divergence time of the 27 bird species was estimated by mcmctree program implemented in PAML v4.9 [74]. Four calibration times obtained from the TimeTree (http://www.timetree.org/) website were set for dating analysis: 26~35 million years ago (Mya) for *Anser cygnoides* and *Anas platyrhynchos*, 33.2~42.3 Mya for *Coturnix japonica* and *Gallus gallus*, 7~19.2 Mya for *Pygoscelis adeliae* and *Pygoscelis antarcticus*, 72~90 Mya for *Egretta garzetta* and *Columba livia*. The contraction and expansion of gene families were estimated using CAFE v4.2.1 [75].

### Genome resequencing

Quality testing included (i) analysis of DNA purity and integrity by agarose gel electrophoresis, (ii) Nanodrop detection of the purity of DNA (OD260/280 ratio), and (iii) qubit quantification of the DNA concentration ratio. The qualified DNA samples of 66 birds were sequenced via Illumina NovaSeq platform with an average coverage depth of ~21.41×. Clean data were obtained after the following processing from raw data: (i) removing adapter paired-end reads, (ii) removing the paired-end read if the N content length exceeds 10% in one single-end read, (iii) deleting the paired-end read if the low-quality (*Q*≤5) base number exceeds 50% in one single-end read. After processing, base quality Q20 and Q30 were 96.82% and 92.03% respectively with an average of 29.56 Gb per sample. The cleaned fastq files were used to detect variants.

### Variant detection

We used the higher-quality genome assembly of *GSA* as a reference to detect variants of 66 birds following the steps: (i) constructing reference genome index by BWA; (ii) aligning the cleaned fastq files of 66 individuals using BWA; (iii) sorting the aligned bam files using SAMtools; (iv) RealignerTargetCreator and IndelRealigner functions in GATK v4.0.2.1 (https://gatk.broadinstitute.org/hc/en-us) were used to mark PCR duplication and re-align; (v) HaplotypeCaller in GATK method was used to detect variants. A total of 126,883,783 variants were acquired after filtration with the criteria of QD < 2.0, FS > 20.0, MQ < 40.0, SOR > 3.0, MQRankSum < −2.0 and ReadPosRankSum < −5.0; (vi) 79,518,440 variations were detected by Platypus v0.8.1 [76]; (vii) 47,617,703 variants from (v) and (vi) intersection were used as the source to detect variants again utilizing Platypus. A total of 13,651,674 variants including 11,105,087 SNPs and 2,546,587 InDels were obtained after hard filtration again using the above criterion. The assembled *GCO* genome was used as a reference to detect variants of 14 *GCO* under the same strategy and 9,398,622 variants were detected after filtering.

### Identification of scaffolds from chromosomes Z and W

We used SAMtools to count the depth of each individual and to identify sex-related scaffolds that belong to chromosomes Z and W by comparing the read depth for each scaffold in ten males and four females. We then downloaded most high-quality avian sex chromosomes from the NCBI database (Additional file 1: Table S27) and then combined these into a synthetic reference genome. The sex-related scaffolds of *GCO* and *GSA* were consistently split into non-overlapping short reads with 300 bp length and aligned to the synthetic reference genome using BWA. SAMtools was then used to extract reads of Mapping Quality > 30 for screening and classification. We discarded reads that aligned on different chromosomes or multi-reads aligned on the same chromosome and kept all results with the same mapping values or one result with the peak score. The scaffolds with a BWA mapping rate > 20% were considered sex scaffolds. The sex-related scaffolds of *GCO* and *GSA* were aligned to the synthetic genome using minimap2 [77]. Finally, the sex-related scaffolds were verified according to the proportion of aligned reads or the results of minmap2. We identified 15 scaffolds in *GCO*, which belonged to chromosome Z with a total length of 86.74 Mb, and 10 scaffolds, which belonged to chromosome W with a total length of 28.67 Mb (Additional file 2: Table S23). For *GSA*, there were 24 scaffolds that belonged to chromosome Z with a total length of 72.59 Mb, and five scaffolds belonged to chromosome W with a total length of 18.61 Mb (Additional file 2: Table S24).

### Genetic structure and divergent time estimation analysis among laughingthrush species

A phylogenetic tree was constructed to explore relationships among the nine species. We extracted SNPs from predicted CDS regions and removed SNPs with LD (*r*^2^) > 0.5. A new dataset of 78,947 SNPs was generated and used for analysis. We set an ultrafast bootstrap approximation (UFBoot = 2000) to assess branch supports [78] and found an optimization model (TVM + F + ASC + G4) using the ModelFinder method [79]. The maximum-likelihood (ML) tree between individuals was estimated through IQ-tree software, and we visualized the tree using Figtree v1.4.4 (http://tree.bio.ed.ac.uk/software/figtree/). The statistics PI_HAT and DST were calculated by the command --genome from PLINK v1.9 [80]. Of which, PI_HAT means shared proportion of identity-by-descent (IBD) and was calculated by formula PI_HAT = P_IBD=2_ + 0.5*P_IBD=1_ and DST was calculated by DST = (IBS2 + 0.5*IBS1)/(IBS0 + IBS1 + IBS2). In this equation, IBS means identity-by-state, IBS0, IBS1, and IBS2 represent the number of IBS 0, 1, and 2 non-missing variants respectively. According to ML-tree and estimated genetic relationship among the 66 individuals. Several individuals appeared to be related (Additional file 1: Table S17 and Fig. S6). The individuals with lower sequencing quality among these related individuals were removed and a total of 58 individuals remained and used to reconstruct ML-tree. The IBS distance matrix among individuals was estimated by command --pairwise-distance from PLINK, visualized by pheatmap package in R language, and we constructed a neighbor joining (NJ) tree using SplitsTree v4.16.1 [81].

We randomly selected five individuals from each *Garrulax* species to establish an accurate taxonomy and divergence time of eight *Garrulax* species. To reduce computation time, 43,475 SNPs in predicted CDS regions with LD (*r*^2^) < 0.5 were reserved. Then, divergence times among *Garrulax* species were estimated by SNAPP software from BEAST v2.6.4 [82]. The input XML file was obtained using the snapp_prep.rb script (https://github.com/mmatschiner/snapp_prep) with a chain length of 1,000,000 MCMC iterations. We randomly selected one individual from the eight *Garrulax* species and constructed a NJ tree as the species tree, and we specified the species NJ tree as a starting tree and placed two calibration points of *TMI* and *GCA* (mean: 14 Mya, SD: 0.995), *GCA* and *GCI* (mean: 11.45 Mya, SD: 1.915) according to TimeTree. Tracer v1.7.1 [83] software was used to check the chain convergence by examining log likelihood plots and ensuring that ESS values were > 200. According to the results of SNAPP, the TreeAnnotator software of BEAST was used to estimate the divergence times among *Garrulax* species and visualized by Figtree. The PCA among the 58 individuals was performed using EIG v6.1.4 [84], and we visualized the first 1–4 principal components (PC) using R. For each *Garrulax* species, six individuals were randomly selected and we extracted 1,043,686 SNPs to estimate the ancestor population number *K* from 2–9 using ADMIXTURE v1.3.0 [85].

### Population inference analysis

For five species with relatively close genetic distance (*GCO*, *GBE*, *GCH*, *GSA*, and *GPE*), we respectively selected one individual with the greatest sequencing quality for each species. The historical population size of these five species before 10–1000 thousand years ago was estimated using parameters of -N25 -t15 -r5 -p "4+25*2+4+6" from PSMC v0.6.5 [86] with an estimated neutral mutation rate *μ* (mutations per base per generation) of 4.44 × 10^–9^ and 3 years for one generation according to our observations from Nanchang Zoo, as well as from field observations [27]. The mutation rate (*μ*) was estimated using the genome-wide nucleotide divergence (3.24%) and divergence time (10.96 million years) between the *GCO* and the *GSA*. We calculated *μ* = (0.0324 × 3)/(2 × 10.96 × 10^6^) = 4.44 × 10^−9^ mutations per generation for the *GCO*, and we used this rate as the spontaneous mutation rate of the laughingthrush. The divergence time between *GCO* and *GSA* was estimated by BEAST, as mentioned above. We then estimated population size for a high-quality *GCO* with 100 bootstraps and all ten *GCO* individuals using PSMC with the same parameters. To reconstruct recent demographics from the last 100,000 years to the present, the popsizeABC software, based on an approximate Bayesian computation approach [87] was used to calculate the effective population size of each species with parameters of mac = 0 (minor allele count threshold for AFS and IBS statistics computation), mac_ld = 4 (minor allele count threshold for LD statistics computation), L = 4000000 (size of each segment in bp), nb_rep = 2500 (number of simulated datasets), nb_seg = 50 (number of independent segments in each dataset), and other parameters set as the default. To further explore the population inference of the *Garrulax* species, we used smc++ v1.5.3 [88] to estimate the effective population size in the past 100,000 years.

### Gene flow analysis

Migration events were estimated using TreeMix v.1.13 [85] with *TMI* as root, 5000 SNPs in each block and bootstrap 1000. The gene flow of the D-statistic among species was calculated by Admixtools v7.0.1 [89], and the *TMI* was set as the outgroup. We set population 1 (P1), population 2 (P2), and population 3 (P3) according to phylogenetic relationship. A *D* value > 0 means that P1 and P3 share more alleles than P2 and P3 share, while a *D* value < 0 means that P2 and P3 share more alleles than P1 and P3 share, which reflects introgression among them.

### Estimation of genetic diversity and inbreeding for each laughingthrush species

Based on the SNPs obtained from the *GCO* and *GSA* genomes, we used VCFtools v0.1.16 [90] to estimate nucleotide diversity (*π*) with non-overlapping 40-kb sliding windows. A total of 28,671 and 25,399 windows were identified and 29,950 and 27,098 windows after polishing in *GCO* and *GSA* respectively. We then estimated *π* for each individual of *GCO* and *GSA* using the same strategy as above. The LD and ROH of the two species (*N*_*GCO*_ = 10, *N*_*GSA*_ = 11) were estimated through command --r2 --ld-window-kb 500 --ld-window-r2 0 and --homozyg-snp 10 --homozyg-kb 40 -homozyg-window-missing 2 in PLINK. Based on SNPs called from the *GSA* genome, we calculated the proportion of the genome in ROH for each laughingthrush, F_ROH_, as the summed length of ROH per individual divided by the total length of *GSA* scaffolds (1.132 Mb). We then calculated the ROH, LD decay, coefficient of inbreeding, and *π* for each laughingthrush via PLINK and VCFtools. The heterozygosity rates for each individual in *GCO* and *GSA* were calculated. Heterozygosity is defined here as the number of heterozygous genotypes across their respective genomes (the denominator means the length of genomes) (Additional file 1: Fig. S20, Table S20).

### Deleterious mutation analysis

Frameshift, stop gain, stop loss, and splicing mutations are considered to be strong deleterious variants, while missense and synonymous mutations are slight deleterious variants. We analyzed the deleterious mutations in the two species following steps: (i) constructing genome database of *GCO* and *GSA* through ANNOVAR [91] and converting gff3 to gtf format through gffread v0.12.4 software [92]; (ii) converting gtf to genePred format by gtftoGenepred; (iii) transcripts in fasta format obtained from retrieve_seq_from_fasta.pl algorithm in ANNOVAR; (iv) using table_annovar.pl in ANNOVAR algorithm to annotate variations of the two species. We then counted the number of each mutation type and visualized them by in-house R scripts.

### Genome-wide selection analysis

*F*_ST_ between ten *GCO* and 47 other *Garrulax* individuals was performed with sliding non-overlapping 40-kb windows across the genome. For each window with the number of SNPs more than 10, we also calculated zHp through formula [93]:

$${H}_p=2\sum {n}_{NAJ}\sum {n}_{MIN}/{\left(\sum {n}_{NAJ}+\sum {n}_{MIN}\right)}^2$$

$$z\mathrm{Hp}=\frac{x-\mu }{\sigma }$$

where *n*_*NAJ*_ and *n*_*MIN*_ represent the frequency of primary and secondary alleles of each SNP respectively, *μ* and *σ* represent mean and standard deviation of *H*_*p*_. We calculated Tajima’s *D* and *π* values with non-overlapping 10-kb sliding windows via VCFtools on regions of extending 500 kb on both sides of the two windows.

### Identification of sex-related scaffolds and genome-wide STRs

To distinguish the sex of *GCO* more easily, we identified the sex-related scaffolds for further research based on the sequencing depth through depth function of SAMtools. To detect STRs, we defined STRs using Tandem Repeat Finder [94] by parameters of Match = 2, Mismatch = 7, Delta = 7, PM = 80, PI = 10, Minscore = 30, and MaxPeriod = 6. Then the whole-genome STRs of *GCO* were detected by GangSTR [95].

## Supplementary Information

**Additional file 1: Fig. S1.** The genome annotation pipeline of the *GCO* and *GSA*. **Fig. S2.** Distribution of the divergence rate of each type of TE in the *GCO* and *GSA* genome. **Fig. S3.** Venn diagram of gene function annotation results of different protein database. **Fig. S4.** Syntenic blocks shared between the *GCO* and *GSA* scaffolds. Grey lines connect matched gene pairs. **Fig. S5.** Enrichment analysis of positive selection genes. **Fig. S6.** The ML tree was constructed based on 66 individuals. **Fig. S7.** The heatmap of identity-by-state distance from 58 individuals. **Fig. S8.** The ML tree of the 58 individuals. **Fig. S9.** ADMIXTURE analysis with ancestral lineages *K* from 2 to 9. **Fig. S10.** CV-error of ADMIXTURE analysis. **Fig. S11.** The phylogenetic relationships among *Garrulax* species constructed by SNAPP software. **Fig. S12.** Treemix phylogeny of *Garrulax* species with *TMI* as root. **Fig. S13.** The population inference estimated by smc++. **Fig. S14.** Population inference in *GCO*. **Fig. S15.** Nucleotide polymorphism (π) of each individual from *GCO* and *GSA*. **Fig. S16.** Nucleotide polymorphism (π) of ten *GCO* individuals. The grey shadow means homozygosis gap region. **Fig. S17.** Nucleotide polymorphism (π) of 11 *GSA* individuals. **Fig. S18.** Distribution of inbreeding coefficient (*F*) in eight *Garrulax* species. **Fig. S19.** Distribution of π value for the eight *Garrulax* species. **Fig. S20.** The scope of π value for eight *Garrulax* species. **Fig. S21.** The scope of heterozygosity rate value for eight *Garrulax* species. **Fig. S22.** LD pattern (left) and ROH number (right) for each *Garrulax* species based on *GSA* genome. **Fig. S23.** The scope of F_ROH_ for eight *Garrulax* species. **Fig. S24.** Comparison missense and synonymous mutations between *GCO* and *GSA*. **Fig. S25.** Distributions of heterozygosity Hp and zHp. **Table S1.** Summary of the sequencing data for the *de novo* genomes. **Table S2.** Assembly statistics of the *GCO* and *GSA* genomes. **Table S3.** Base contents of the assembled genomes. **Table S4.** Read coverage of genome assemblies. **Table S5.** The completeness of the two assembled genomes was assessed by CEGMA and BUSCO. **Table S6.** Prediction of repeat elements in the two assembled genomes. **Table S7.** Categories of repeat elements. **Table S8.** Prediction of gene structure in two genomes. **Table S9.** Gene structure of genomes of *Garrulax* species and other avians. **Table S10.** Functional annotation of the predicted protein-coding genes in the *Garrulax* genome assemblies. **Table S11.** Statistics of noncoding RNAs of the *GCO* and *GSA* genomes. **Table S12.** Positive selection genes (PSGs) identified in *GCO* and *GSA*. **Table S13.** The information of 25 species download from NCBI database. **Table S14.** Expanded and contracted gene families identified by CAFE. **Table S15.** Sequencing data quality of 66 individuals. **Table S16.** Detailed sampling information of 66 sequenced individuals. **Table S17.** Pair individuals with closely relationship of 66 specimens. **Table S18.** Evidence of gene flow between *Garrulax* species. **Table S19.** Genetic diversity of eight *Garrulax* species based on a reference genome of white-browed laughingthrush*.* **Table S20.** Deleterious variants of blue-crowned laughingthrush and white-browed laughingthrush. **Table S22.** The overlapping windows identified by top 1% *F*_ST_ and zHp value. **Table S25.** The 218 STRs makersof blue-crowned laughingthrush. **Table S26.** The 43 STRs were identified on W-related scaffolds to be used as sex confirmation. **Table S27.** The Z and W chromosomes of 31 avian.

**Additional file 2: Table S21.** The heterozygosity rate of *GCO*, *GSA*, and the other 186 species. **Table S23.** Scaffolds belong to chromosome Z and W and the depth information of *GCO*. **Table S24.** Scaffolds belong to chromosome Z and W and the depth information of *GSA*.

## Data Availability

Genomic data of blue-crowned laughingthrush and white-browed laughingthrush were uploaded to the National Genomics Data Center (NGDC) under accession number PRJCA005228 [96]. The raw sequencing data of 66 birds is available from NGDC under accession number PRJCA005263 [97]. Analytical pipelines and code are available on the Zenodo (doi: 10.5281/zenodo.6570622) [98].

## Abbreviations

*GCO*: *Garrulax courtoisi*
CR: Critically endangered
IUCN: International Union for Conservation of Nature
*GSA*: *Garrulax sannio*
LC: Least concern
CEGMA: Core Eukaryotic Genes Mapping Approach
PCA: Principal component analysis
ML: Maximum-likelihood
*GCA*: *Garrulax canorus*
*GCI*: *Garrulax cineraceus*
*GBE*: *Garrulax berthemyi*
SNP: Single-nucleotide polymorphisms
LD: Linkage disequilibrium
ROH: Run of homozygosity
STR: Short tandem repeats
PIC: Polymorphism information content
CCY: Cyanopica cyana
CEGMA: Core Eukaryotic Genes Mapping Approach
TRF: Tandem Repeats Finder
NJ: Neighbor joining
PC: Principal components
