# Supplementary material for: Genomic signatures and evolutionary history of the endangered blue-crowned laughingthrush and other Garrulax species
Source: BMC Biol. 2022 Aug 24;20:188. doi: 10.1186/s12915-022-01390-4 (PMC9400264; doi:10.1186/s12915-022-01390-4)
Supplement: Supplementary file 1 — Additional file 1: Fig. S1. The genome annotation pipeline of the GCO and GSA. Fig. S2. Distribution of the divergence rate of each type of TE in the GCO and GSA genome. Fig. S3. Venn diagram of gene function annotation results of different protein database. Fig. S4. Syntenic blocks shared between the GCO and GSA scaffolds. Grey lines connect matched gene pairs. Fig. S5. Enrichment analysis of positive selection genes. Fig. S6. The ML tree was constructed based on 66 individuals. Fig. S7. The heatmap of identity-by-state distance from 58 individuals. Fig. S8. The ML tree of the 58 individuals. Fig. S9. ADMIXTURE analysis with ancestral lineages K from 2 to 9. Fig. S10. CV-error of ADMIXTURE analysis. Fig. S11. The phylogenetic relationships among Garrulax species constructed by SNAPP software. Fig. S12. Treemix phylogeny of Garrulax species with TMI as root. Fig. S13. The population inference estimated by smc++. Fig. S14. Population inference in GCO. Fig. S15. Nucleotide polymorphism (π) of each individual from GCO and GSA. Fig. S16. Nucleotide polymorphism (π) of ten GCO individuals. The grey shadow means homozygosis gap region. Fig. S17. Nucleotide polymorphism (π) of 11 GSA individuals. Fig. S18. Distribution of inbreeding coefficient (F) in eight Garrulax species. Fig. S19. Distribution of π value for the eight Garrulax species. Fig. S20. The scope of π value for eight Garrulax species. Fig. S21. The scope of heterozygosity rate value for eight Garrulax species. Fig. S22. LD pattern (left) and ROH number (right) for each Garrulax species based on GSA genome. Fig. S23. The scope of FROH for eight Garrulax species. Fig. S24. Comparison missense and synonymous mutations between GCO and GSA. Fig. S25. Distributions of heterozygosity Hp and zHp. Table S1. Summary of the sequencing data for the de novo genomes. Table S2. Assembly statistics of the GCO and GSA genomes. Table S3. Base contents of the assembled genomes. Table S4. Read coverage of genome assemblies. Table [file 12915_2022_1390_MOESM1_ESM.docx]

# Genomic signatures and evolutionary history of the endangered blue-crowned laughingthrush and other *Garrulax* species

Hao Chen^1†^, Min Huang^2†^, Daoqiang Liu^3†^, Hongbo Tang^1^, Sumei Zheng^2^, Jing Ouyang^1^, Hui Zhang^2^, Luping Wang^1^, Keyi Luo^1^, Yuren Gao^1^, Yongfei Wu^1^, Yan Wu^1^, Yanpeng Xiong^1^, Yuxuan Huang^1^, Rui Xiong^1^, Jun Ren^2^, Jianhua Huang^1*^, Xueming Yan^1*^

^1^ College of Life Science, Jiangxi Science & Technology Normal University, Nanchang, Jiangxi province, China

^2^ College of Animal Science, South China Agricultural University, Guangzhou, Guangdong

^3^ Nanchang zoo, Nanchang, Jiangxi province, China

^†^ These authors contributed equally to this work.

^*^ Corresponding author. Email: huangjh1113@sina.com, xuemingyan@hotmail.com.

Content

[Genomic signatures and evolutionary history of the endangered blue-crowned laughingthrush and other *Garrulax* species 1](#_Toc107475151)

[Method S1. Genome assembly 2](#_Toc107475152)

[Stage 1: Initial PacBio assembly 3](#_Toc107475153)

[Stage 2: 10X genomics assisted the assembly 4](#_Toc107475154)

[Stage 3: Polishing 4](#_Toc107475155)

[Stage 4: Validation of genomes assembly 5](#_Toc107475156)

[Method S2. Gene annotation 6](#_Toc107475157)

[Stage 1: Repeat annotation 6](#_Toc107475158)

[Stage 2: Gene structure annotation 7](#_Toc107475159)

[Stage 3: Gene function annotation 8](#_Toc107475160)

[Stage 4: non-coding RNA annotation 8](#_Toc107475161)

[Supplementary figures 9](#_Toc107475162)

[Supplementary tables 28](#_Toc107475163)

[References 59](#_Toc107475164)

# Method S1. Genome assembly

*De novo* assemblies for *GCO* and *GSA* used the combination of third-generation single-molecule real-time sequencing (PacBio SMRT), second-generation sequencing platform (Illumina NovaSeq 6000), and 10× Genomics Linked-reads technology. The detailed stages of assembling *GCO* and *GSA* genomes were as follows:

## Stage 1: Initial PacBio assembly

In this study, the third-generation sequencer (PacBio Sequel I) from Pacific Biosciences was used to perform single-molecule real-time sequencing on *GCO* and *GSA*. The qualified genomic DNA was fragmented by 26 G Needle, and then PacBio SMRTbell libraries (20 Kb insert) were prepared for single-molecule real-time sequencing. SMRT bell template preparation involved DNA concentration, damage repair, end repair, ligation of hairpin adapters, and template purification. 113.43 Gb (101.21$\times$) and 115.92 Gb (97.76$\times$) long-read sequencing data for *GCO* and *GSA* were obtained, respectively. Then wtdbg2 software [1] was used to directly assemble the long-read obtained from third-generation sequencing of Pacific Biosciences. The basic process of software assembly is summarized as follows:

1) Alignment: Kmer-Bin-Mapping (KBM) algorithm was used to cut the sequenced long reads into several bins with 256-bp as a unit without overlapping. The *K*-mers in the fragment were then classified into a bin, and a *K*-mer has at least 4$\times$ 256-bp. If most *K*-mers in a bin occur at a higher frequency, the bin will be filtered out as a high repeat bin. Subsequently, based on the dynamic programming algorithm, we search for the linear relationship between the bin pairs that match each other between different sequences.

2) Assembly: Based on the Fuzzy-Bruijn Graph (FBG) algorithm, the reads are interrupted into multiple 1024-bp *K*-mers as vertices in FBG graph, and then these points were connected to form FBG graph according to the reading path, and contigs were obtained by simplifying FBG graph.

3) Polishing: To improve the accuracy of the results, the Quiver software (https://www.pacb.com/tag/quiver/) in the PacBio Genomic Consensus software package is used to correct the results of the third-generation data assembly.

## Stage 2: 10X genomics assisted the assembly

An automated microfluidic system allows the combination of the functionalized gel beads and high molecular weight DNA (HMW gDNA) together with oil to form a gel bead in emulsion (GEM). Each GEM contains ~10 molecules of HMW gDNA and primers with unique barcodes and P5 sequencing adapters. After PCR amplification, P7 sequencing adapters are added for Illumina sequencing. Then we generated a total of 111.9 Gb (99.9$\times$) and 122.52 Gb (103.3$\times$) linked-reads for *GCO* and *GSA*, respectively.

Mapping the generated linked-reads to the consensus sequence obtained in stage 1 to further connect and assemble the consensus sequence according to the actual distance. The linkage was removed if it was supported by an unreliable weight of paired-end relationships. And then, the super-scaffolds of *GCO* and *GSA* were generated.

## Stage 3: Polishing

Total genomic DNA of *GCO* and *GSA* was extracted from peripheral blood. Two paired-end libraries for genomic sequencing were constructed with 350 bp insert size, and sequencing was carried out on the Illumina NovaSeq 6000 platform according to the standardized process of manufacturer’s instructions. Finally, we produced 134.1 Gb (119$\times$) and 113.17 Gb (95.4$\times$) sequencing data in *GCO* and *GSA*, respectively. The low-quality reads were filtered satisfying the following conditions: (i) reads with ≥10% unidentified nucleotides (N); (ii) reads with adapters and (iii) reads with >20% bases having Phred quality <5. Then, Illumina paired-end reads were used to polish the assembled super-scaffold and the specific process was as follows:

1) The reads generated by Illumina sequencing were aligned to the super-scaffold by BWA software [2], and the bam files were obtained and sorted.

2) Subsequently, combined with the mapping results, the assembly version was polished iteratively 2 to 3 times to further fill in the “N” and to improve the single-base correct rate using pilon v1.23 software [3].

Finally, the size of the haploid genomes of *GCO* and *GSA* were estimated to be 1,120.72 Mb and 1,185.73 Mb, respectively.

## Stage 4: Validation of genomes assembly

We re-aligned the Illumina short reads to the two assembled *Garrulax* genomes and counted the mapping rate, coverage and homozygous SNP ratios to evaluate the accuracy of genome assemblies. The integrity of two genomes was assessed based on 248 core genes in Core Eukaryotic Genes Mapping Approach (CEGMA)[4] and 2,586 Benchmarking universal single-copy orthologs (BUSCO) from the Vertebrata (odb9) database [5]. CEGMA assessment combined with tblastn v2.2.26 [6], genewise v2.4.1 [7], and geneid v1.4.5 software (https://genome.crg.es/software/geneid/). BUSCO assessment based on tblastn, Augustus v3.3.3 [8], and hmmer [9] to assess the integrity of assembled genomes.

# Method S2. Gene annotation

## Stage 1: Repeat annotation

Repeat elements occupy a major proportion of the nuclear DNA in most eukaryotic genomes. A combined strategy based on homology alignment and *de novo* search to identify the whole genome repeats of *GCO* and *GSA* was applied in our repeat annotation pipeline. Tandem Repeat was extracted using TRF v4.09 (http://tandem.bu.edu/trf/trf.html) by *ab initio* prediction. The homolog prediction commonly used Repbase (http://www.girinst.org/repbase) database employing RepeatMasker (http://www.repeatmasker.org/) software to develop *de novo* transposable element library and its in-house scripts (RepeatProteinMask) with default parameters to conduct WU-BLASTX searches against the transposable element protein database. And *ab initio* prediction built *de novo* repetitive elements database by LTR_FINDER v1.0.7 (http://tlife.fudan.edu.cn/ltr_finder/), RepeatScout v1.0.5 (http://www.repeatmasker.org/), RepeatModeler v1.0.3 (http://www.repeatmasker.org/RepeatModeler.html) with default parameters, then all repeat sequences with lengths > 100 bp and gap ‘N’ less than 5% constituted the raw transposable element (TE) library. A custom library (a combination of Repbase and our *de novo* TE library which was processed by uclust (http://www.drive5.com/usearch/) to yield a non-redundant library) was supplied to RepeatMasker for DNA-level repeat identification. Overlapping transposable elements belonging to the same type of repeats were integrated.

## Stage 2: Gene structure annotation

After repeats’ masking, we used a combination of homology-based prediction, *de novo* prediction, and full length RNA-Seq assisted prediction to predict structural annotation for the two *de novo* genomes. For gene prediction based on *ab initio*, Augustus v3.3.3 [8], GlimmerHMM v3.0.4 [10] and SNAP v2013.11.29 [11], Geneid v1.4 and Genescan v1.0 were used in our automated prediction pipeline of gene structure for *GCO* and *GSA*. Afterward, we downloaded the coding sequences of the homologous species (African ostrich (*Struthio camelus*), crested ibis (*Nipponia nippon*), Peking duck (*Anas platyrhynchos*), red junglefowl (*Gallus gallu*), turkey (*Meleagris gallopavo*) and Zhedong white goose (*Anser cygnoides*) from Ensembl and NCBI, and aligned them to *GCO* and *GSA* genomes using tblastn with an e-value cutoff by 1e-5, and then the matching proteins were aligned to the homologous genome sequences for accurate spliced alignments with GeneWise v2.4.1 software which was used to predict gene structure contained in each protein region. To optimize the genome annotation, the RNA-Seq reads from different tissues (liver and muscle) were aligned to *GCO* and *GSA* genomes using TopHat v2.0.11 [12] with default parameters to identify exons region and splice positions. The alignment results were then used as input for Cufflinks v2.2.1 [13] with default parameters for genome-based transcript assembly. The non-redundant reference gene sets were generated by merging genes predicted by EvidenceModeler (EVM) v1.1.1 (http://evidencemodeler.sourceforge.net/), using Program to Assemble Spliced Alignment (PASA, <http://pasa.sourceforge.net/>) terminal exon support and including masked transposable elements as input into gene prediction.

## Stage 3: Gene function annotation

After acquiring gene structure information, we predicted function of the proteins by transferring annotations from the closest BLAST hit (e-value <10^-5^) in the Swissprot database and BLAST hit (e-value <10^-5^) in the NR database. We also mapped gene set to a KEGG pathway and identified the best match for each gene. Protein domains were annotated by searching InterPro (https://www.hindawi.com/) and Pfam (http://pfam.xfam.org/) databases, using InterProScan [14] and Hmmer v3.1 (http://hmmer.org/) respectively. Gene Ontology (GO) terms for each gene were obtained from the corresponding InterPro or Pfam entry. The pathways, in which the gene might be involved, were assigned by blast against the KEGG database (https://www.genome.jp/kegg/), with an e-value cutoff of 1e-5.

## Stage 4: non-coding RNA annotation

The structural characteristics of tRNA genes were identified by tRNAscan-SE v1.4[15] software (http://lowelab.ucsc.edu/tRNAscan-SE/). The rRNA fragments were predicted by aligning to the rRNA sequences database using blastn at e-value of 1e-10. Other ncRNAs, including miRNAs, snRNAs were identified by searching against the Rfam database (<http://rfam.xfam.org/>) with default parameters using the INFERNAL [16] software (<http://infernal.janelia.org/>).

# Supplementary figures

**
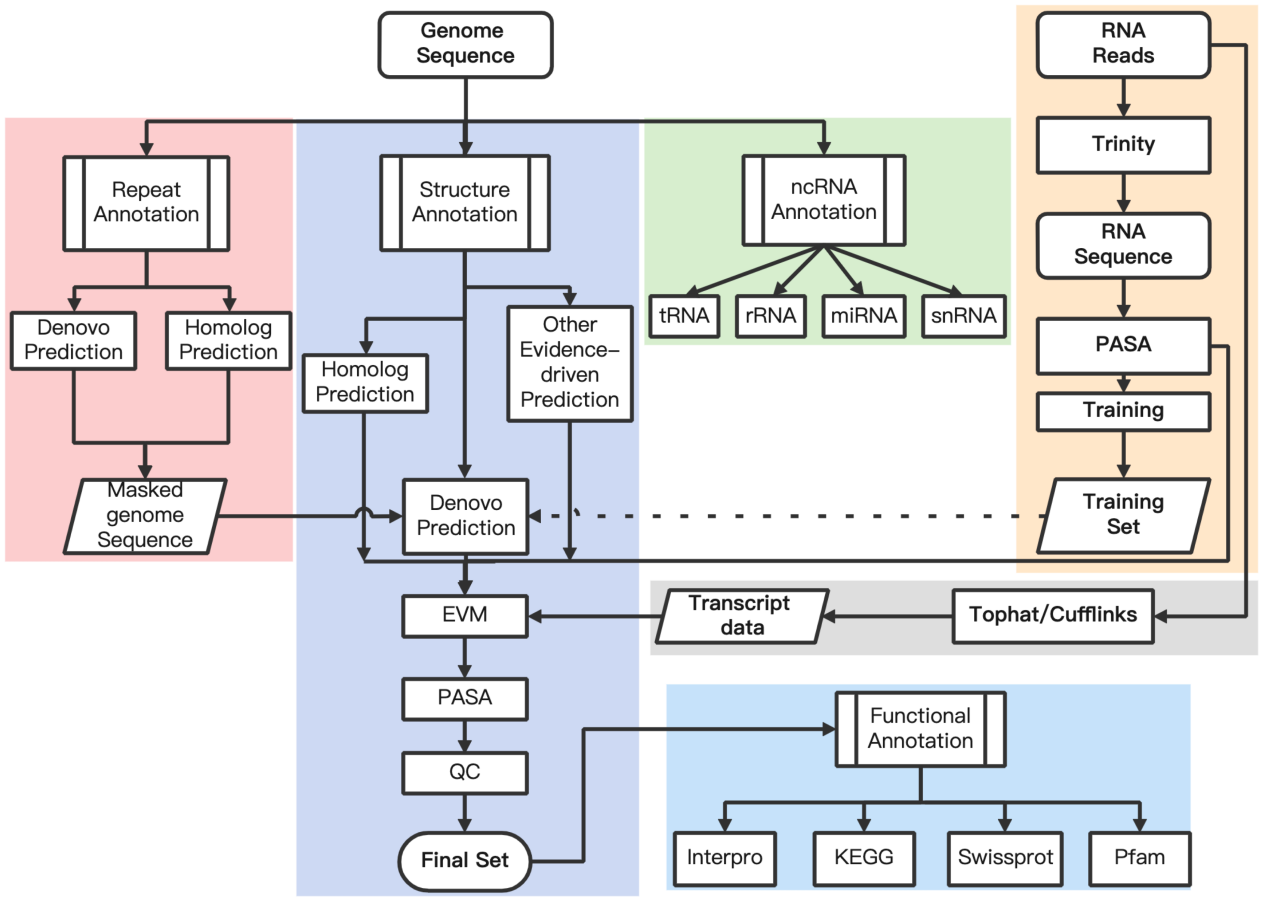
**

**Fig. S1. The genome annotation pipeline of the *GCO* and *GSA*.**

**
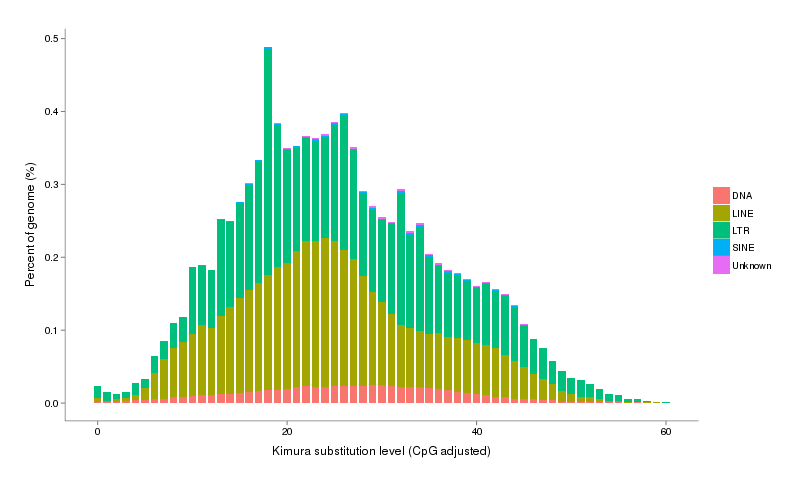
**

**Fig. S2a. Distribution of the divergence rate of each type of TE in the *GCO* genome.** The divergence rate was calculated between the identified TE elements in the genome and the consensus sequence in Repbase.

**
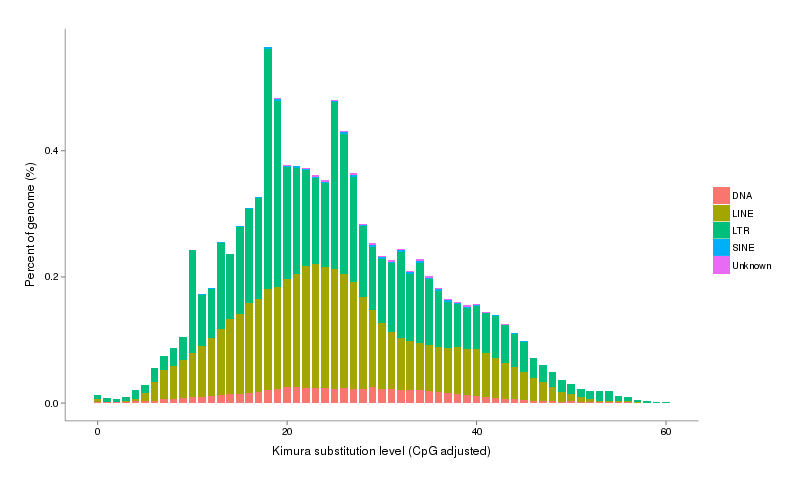
**

**Fig. S2b. Distribution of the divergence rate of each type of TE in the *GSA* genome.** The divergence rate was calculated between the identified TE elements in the genome and the consensus sequence in Repbase.

**
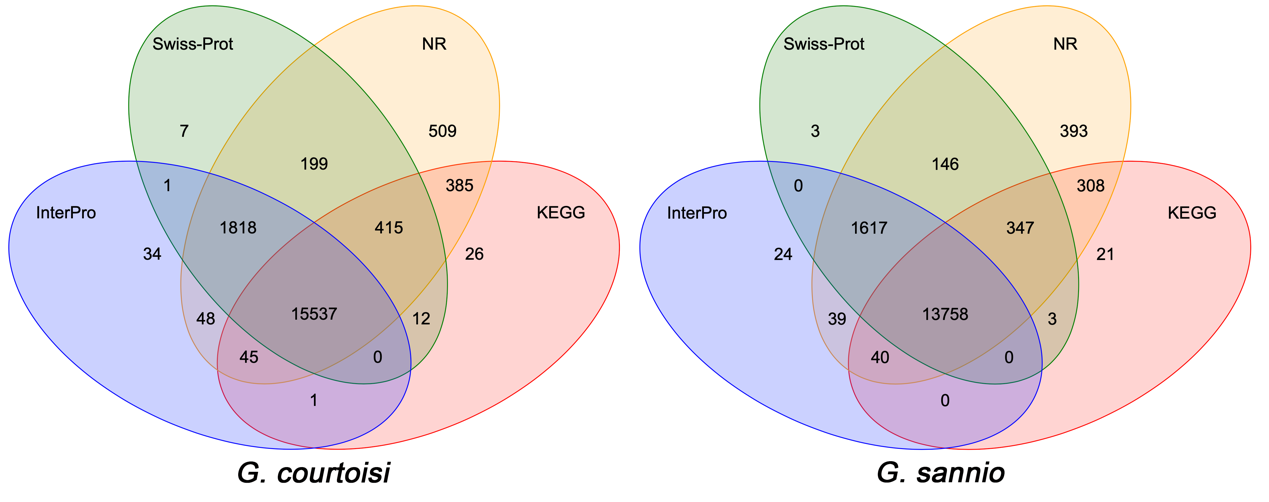
**

**Fig. S3. Venn diagram of gene function annotation results of different protein database.**


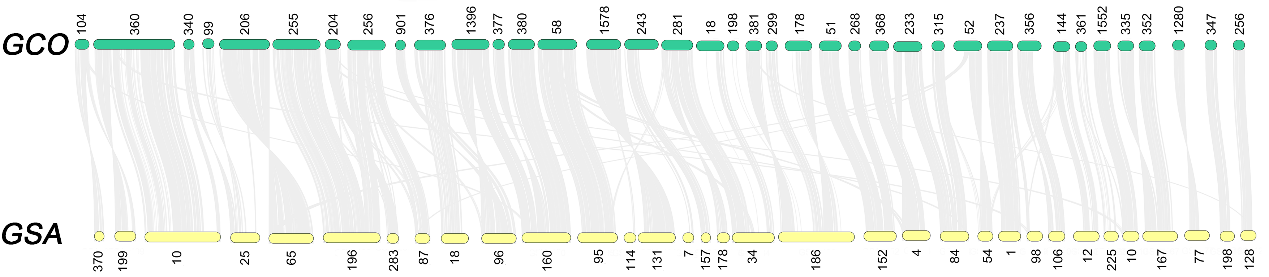


**Fig. S4. Syntenic blocks shared between the GCO and GSA scaffolds. Grey lines connect matched gene pairs.**

**
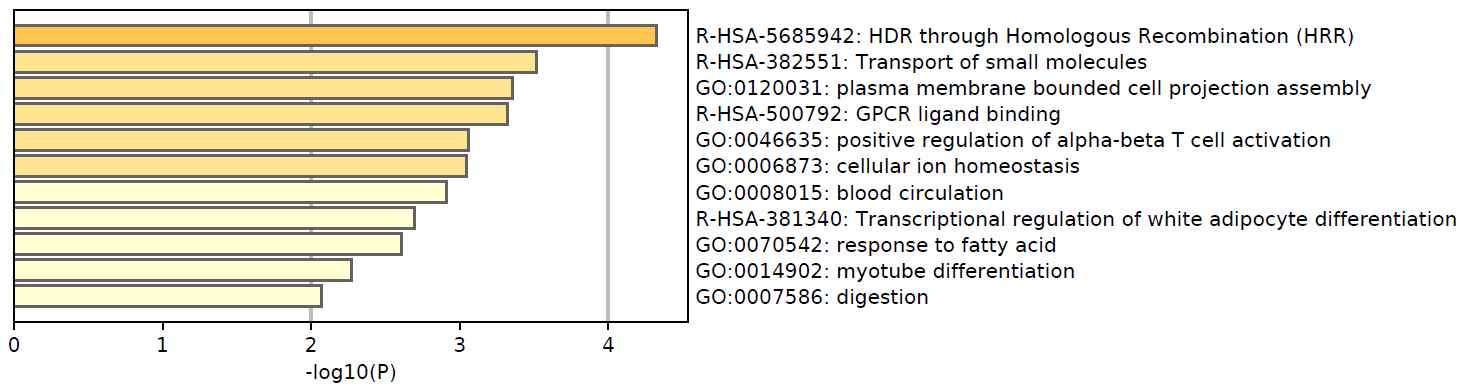
**

**Fig. S5. Enrichment analysis of positive selection genes.**

**
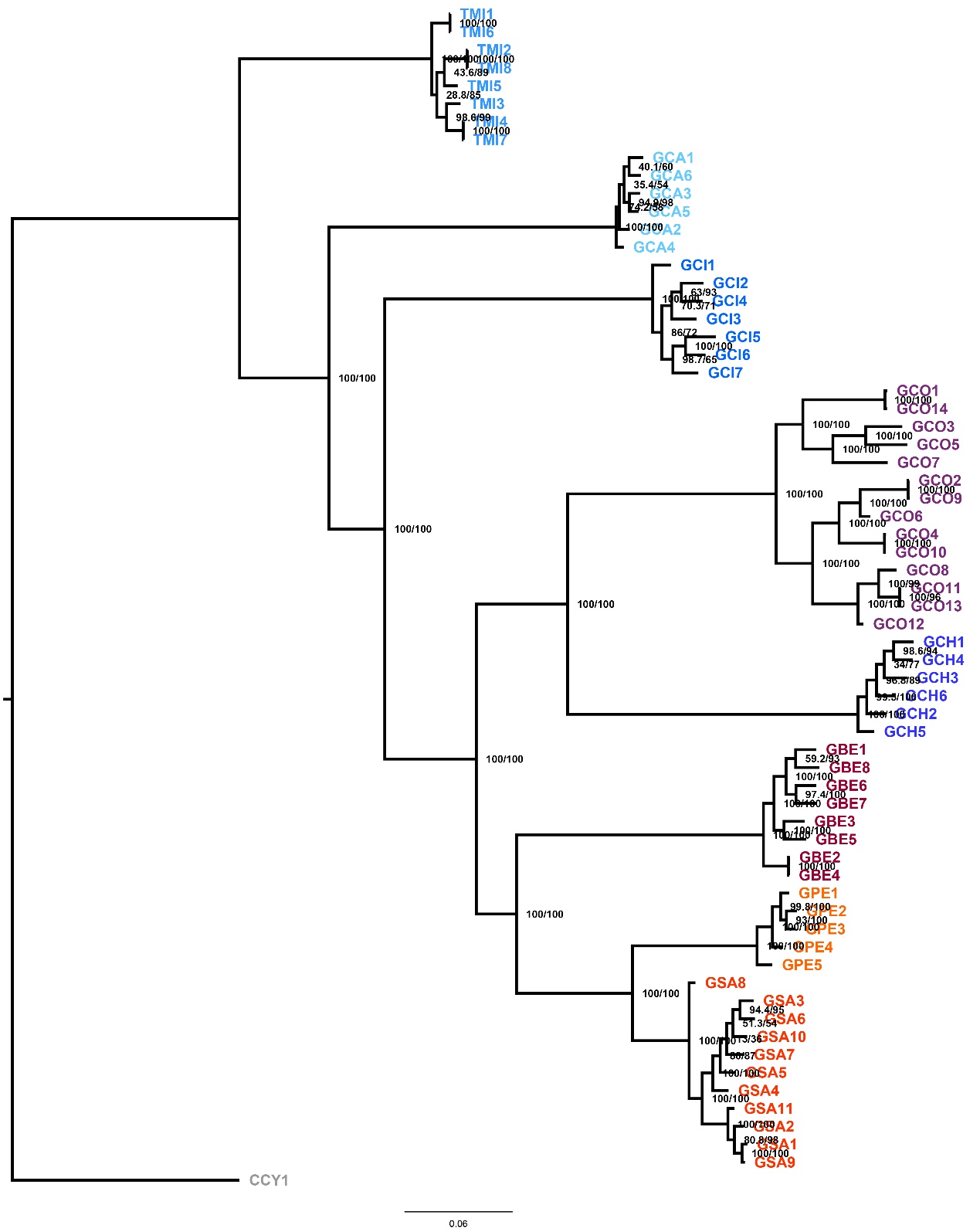
**

**Fig. S6. The ML tree was constructed based on 66 individuals.**

**
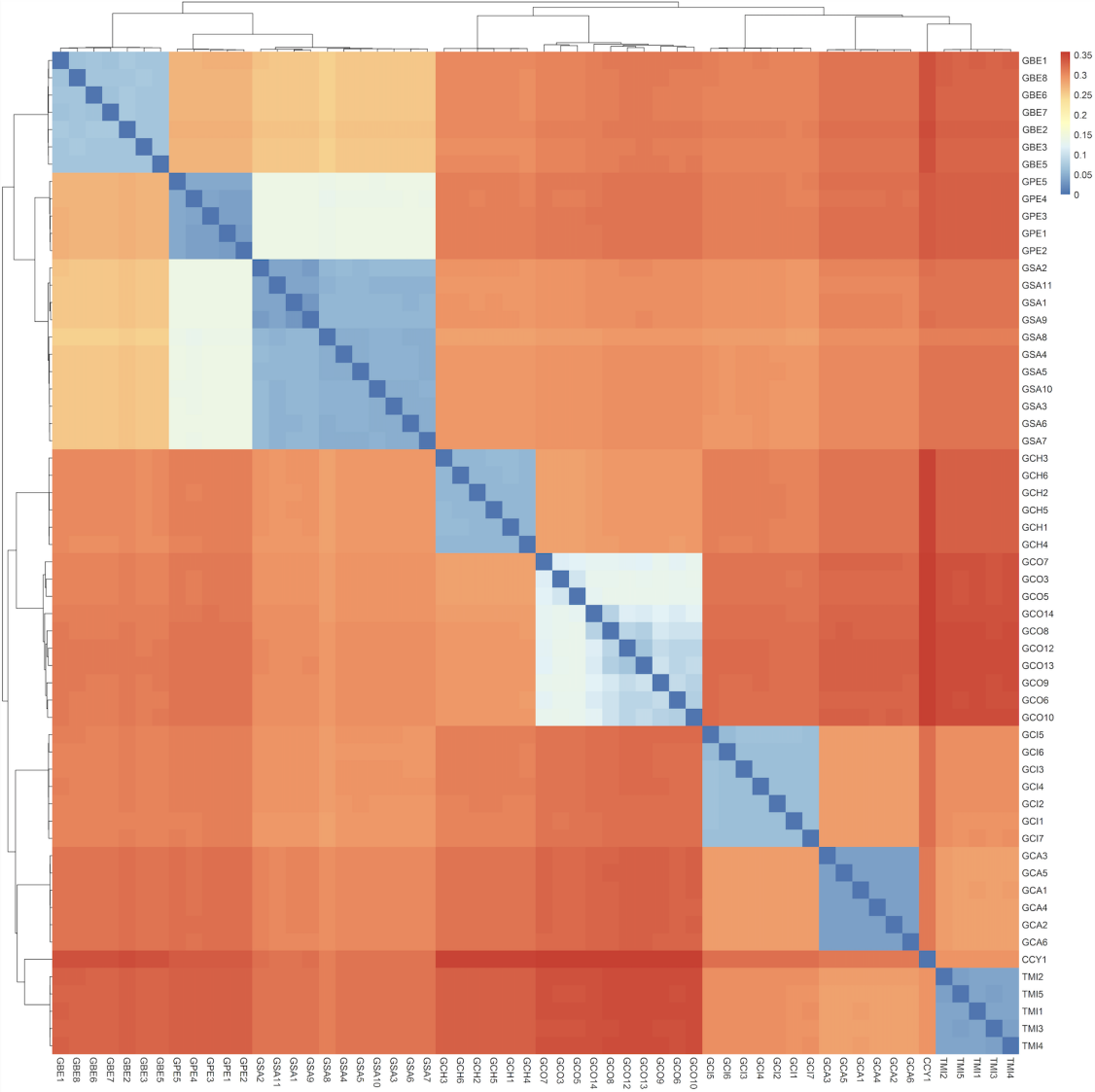
**

**Fig. S7. The heatmap of identity-by-state distance from 58 individuals.**

**
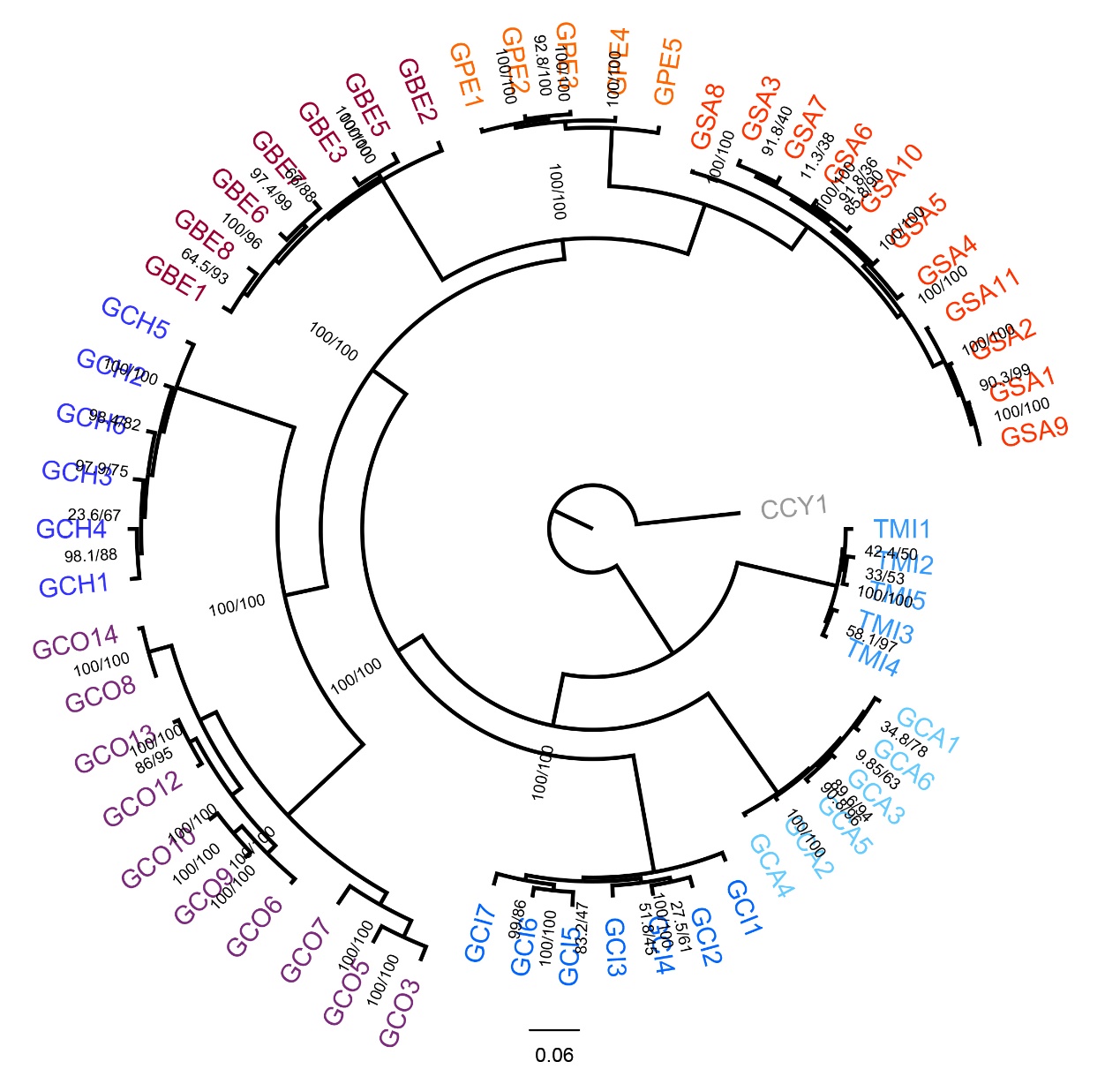
**

**Fig. S8. The ML tree of the 58 individuals.**

**
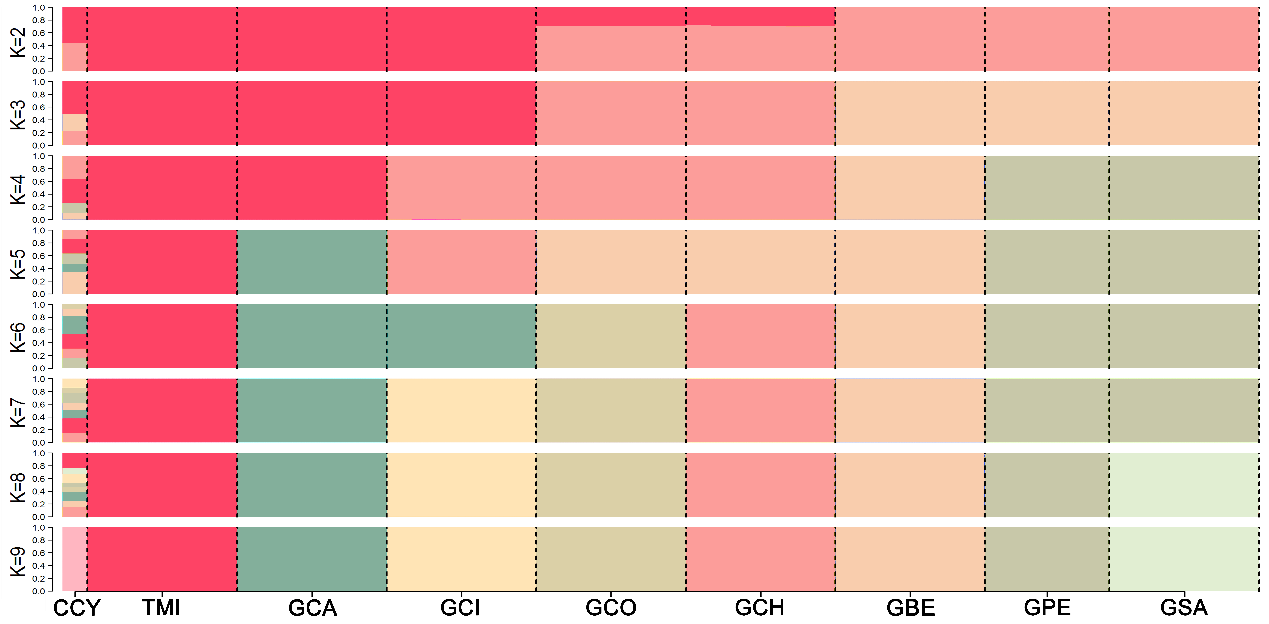
**

**Fig. S9. ADMIXTURE analysis with ancestral lineages *K* from 2 to 9.**

**
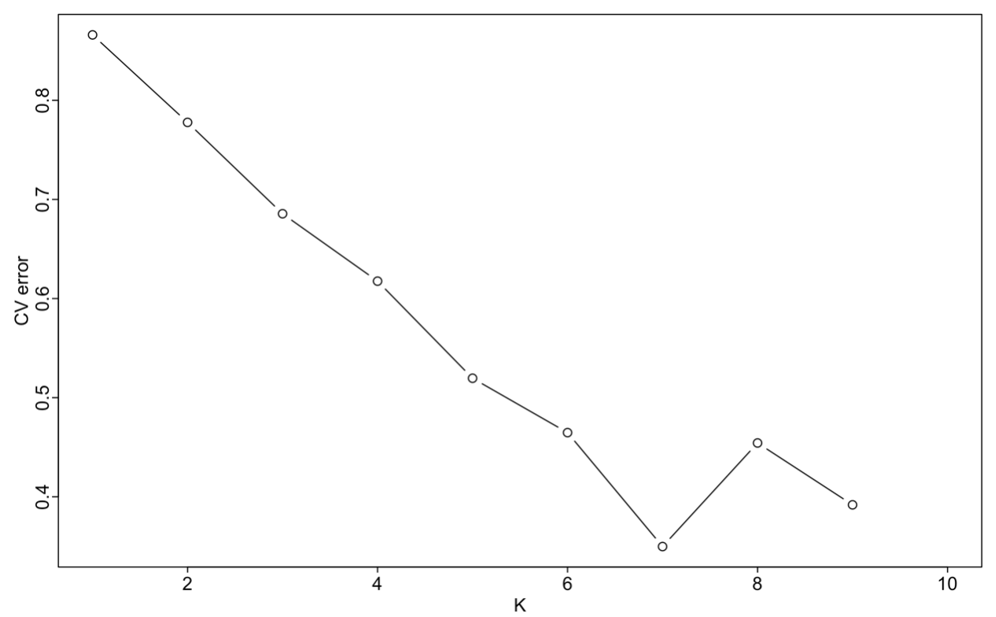
**

**Fig. S10. CV-error of ADMIXTURE analysis.**


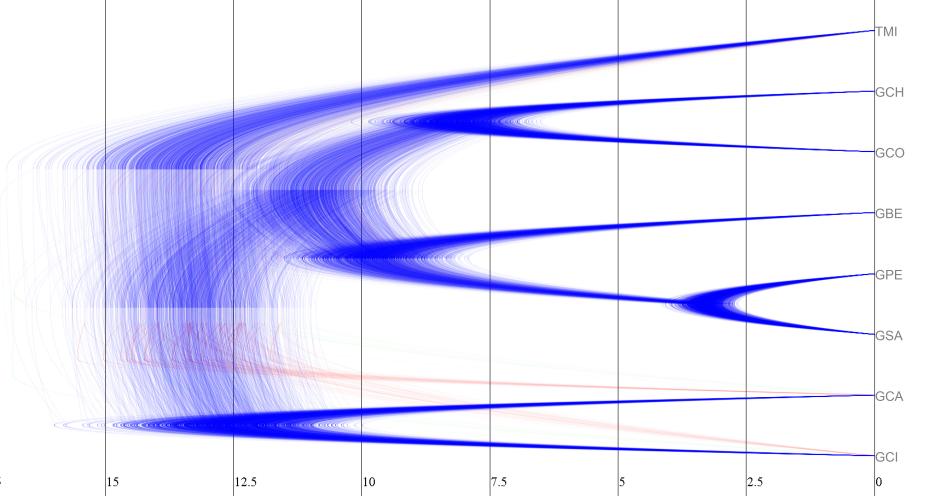


**Fig. S11. The phylogenetic relationships among *Garrulax* species constructed by SNAPP software.**

**
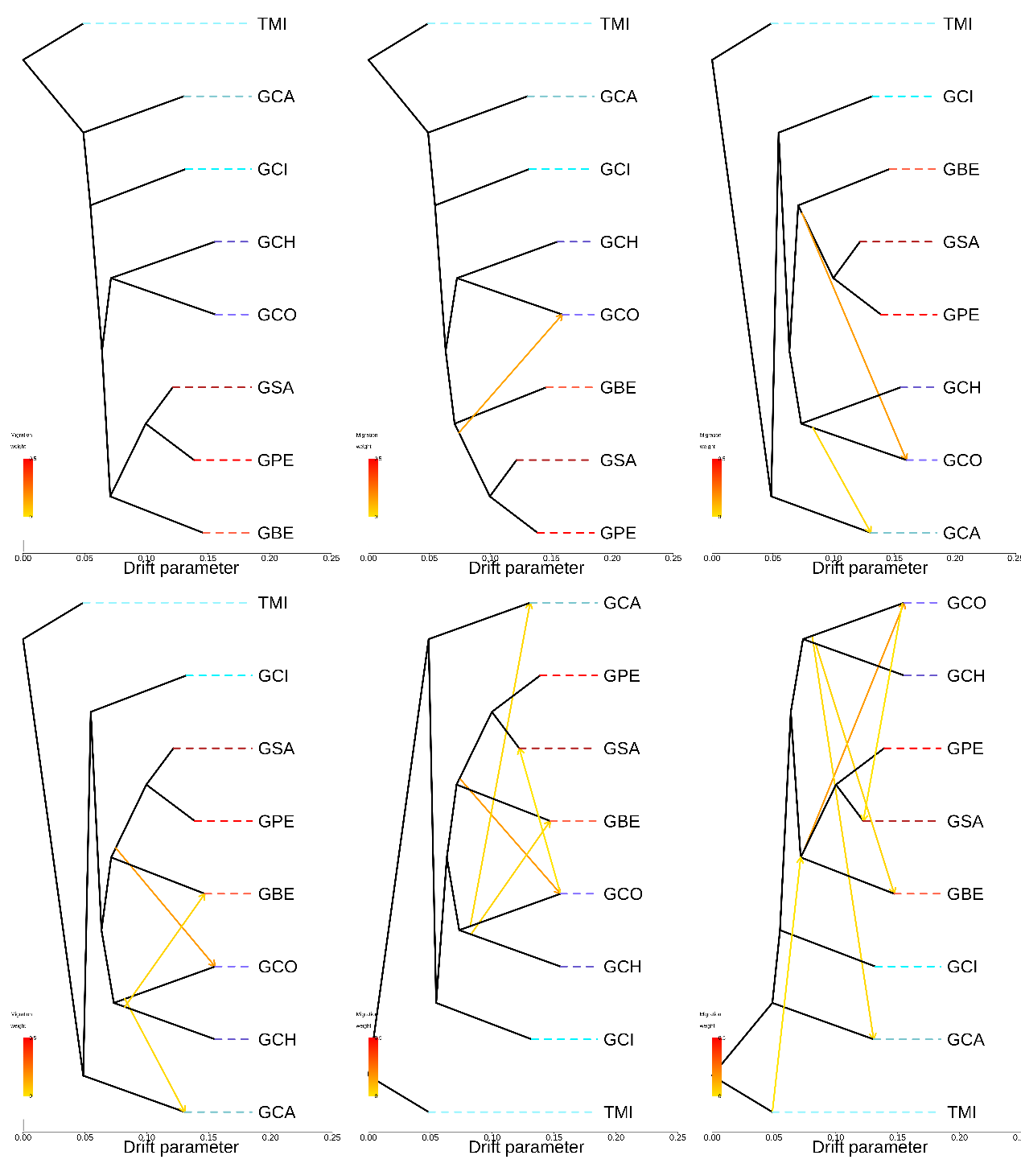
**

**Fig. S12. Treemix** **phylogeny of *Garrulax* species with *TMI* as root.** Straight lines with arrow represent migration events, and their color shades represent weights.


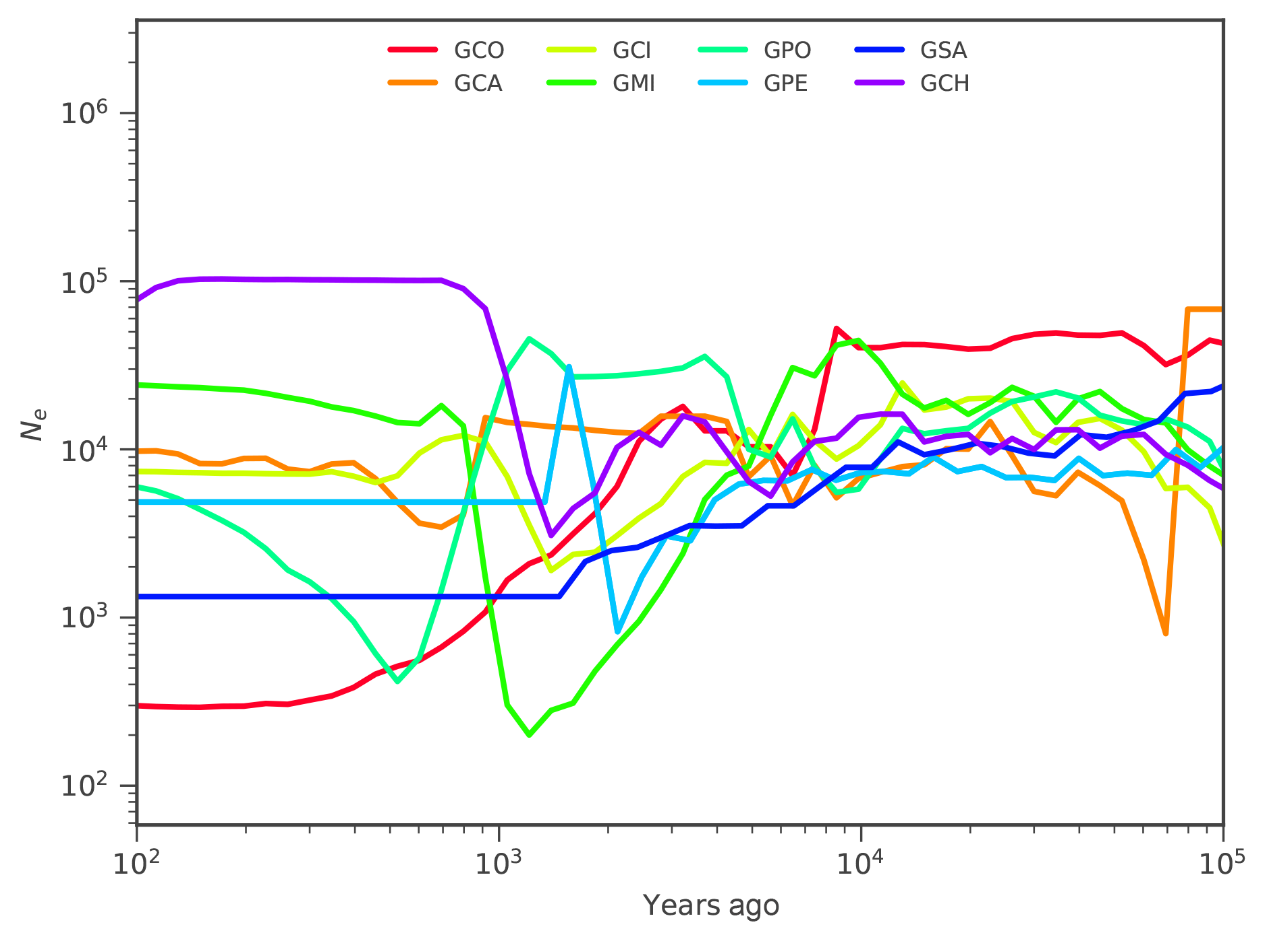


**Fig. S13. The population inference estimated by smc++ .** The x coordinate represents years, and the y coordinate represents effective population size.

**
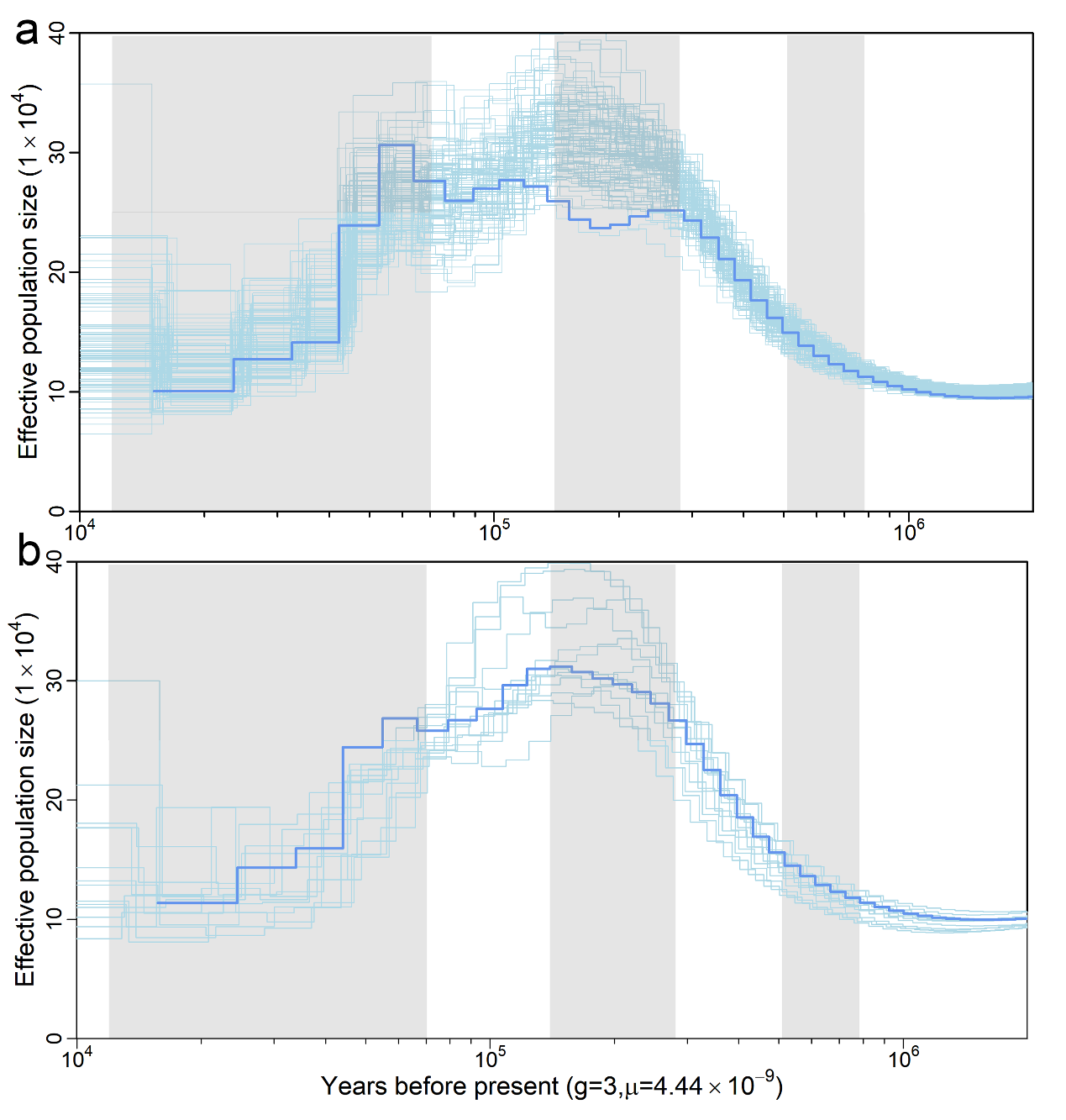
**

**Fig. S14. Population inference in *GCO*.** (a) Estimated population size form one *GCO* with the best sequencing quality using 100 bootstraps based on PSMC. (b) The recent historical population size for ten unrelated individuals in *GCO*. The grey shadows represent glacial time, please refer to Fig. 1e.

**
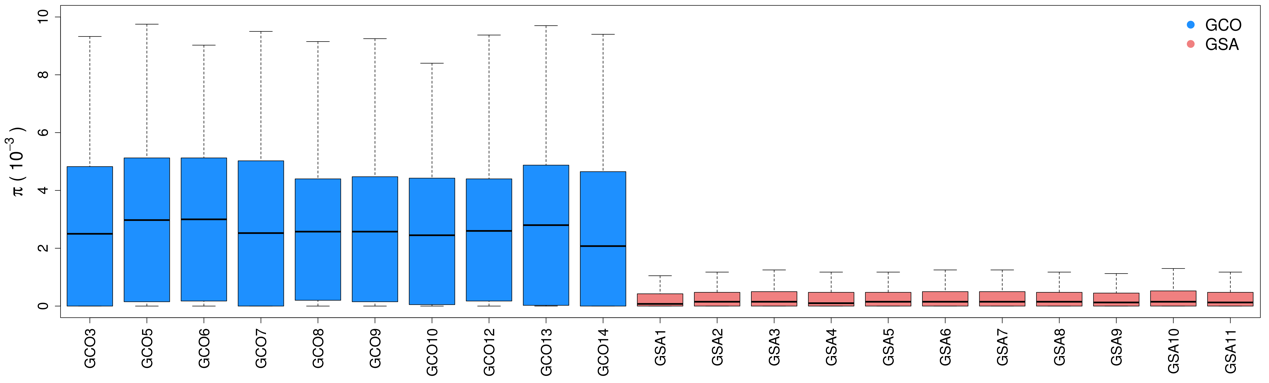
**

**Fig. S15. Nucleotide polymorphism (**$\boldsymbol{\pi}$**) of each individual from *GCO* and *GSA*.**

**
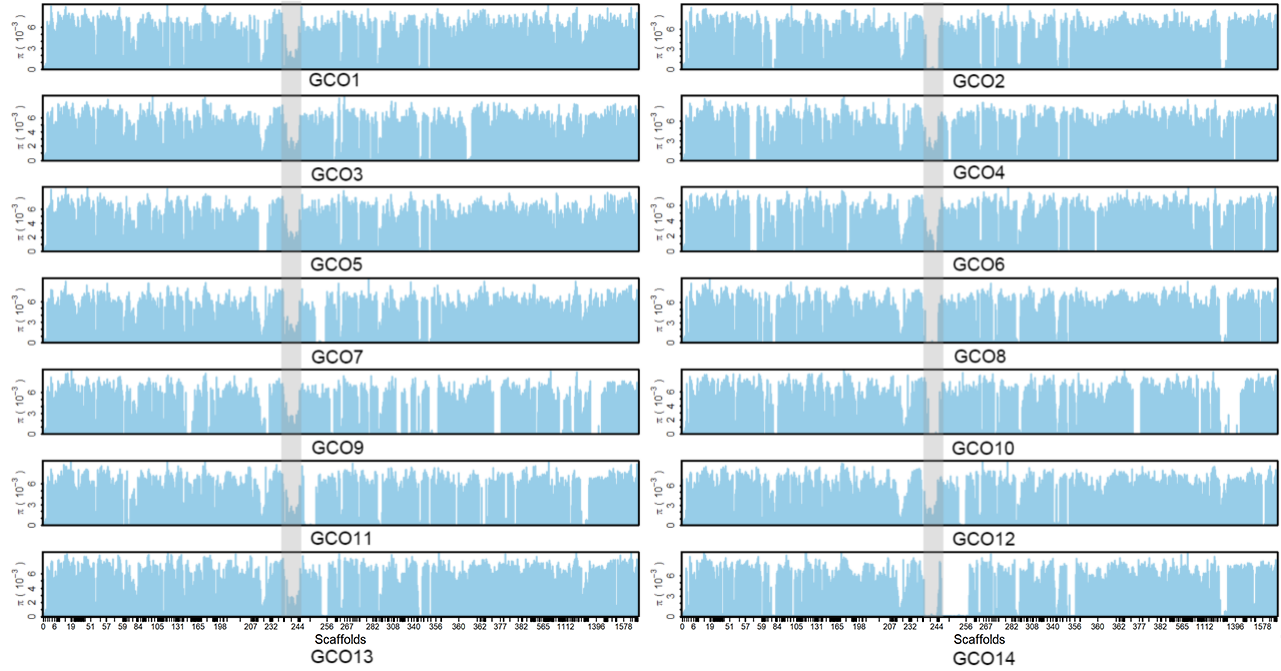
**

**Fig. S16. Nucleotide polymorphism (**$\boldsymbol{\pi}$**) of ten *GCO* individuals. The grey shadow means homozygosis gap region.** The shaded areas are regions with low heterozygosity comparing with the whole genome in *GCO*.

**
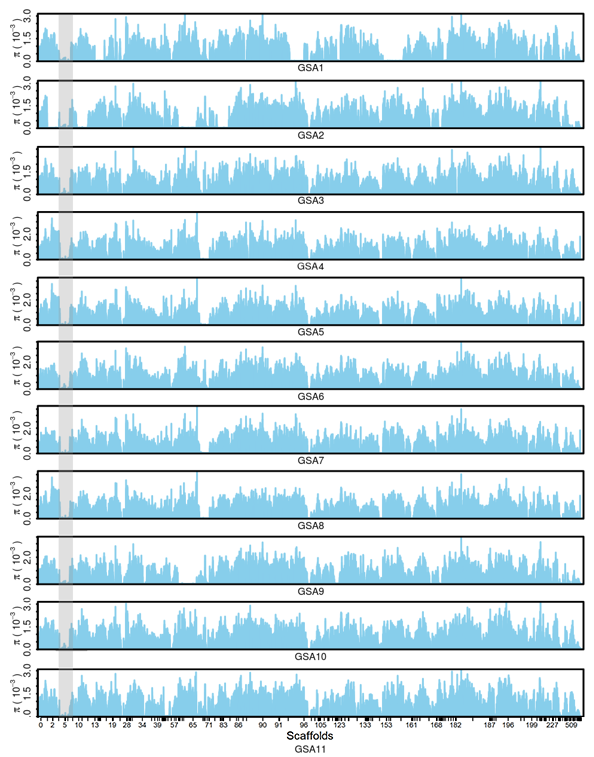
**

**Fig. S17. Nucleotide polymorphism (**$\boldsymbol{\pi}$**) of 11 *GSA* individuals.** The shaded areas are regions with low heterozygosity comparing with the whole genome in *GSA*

**
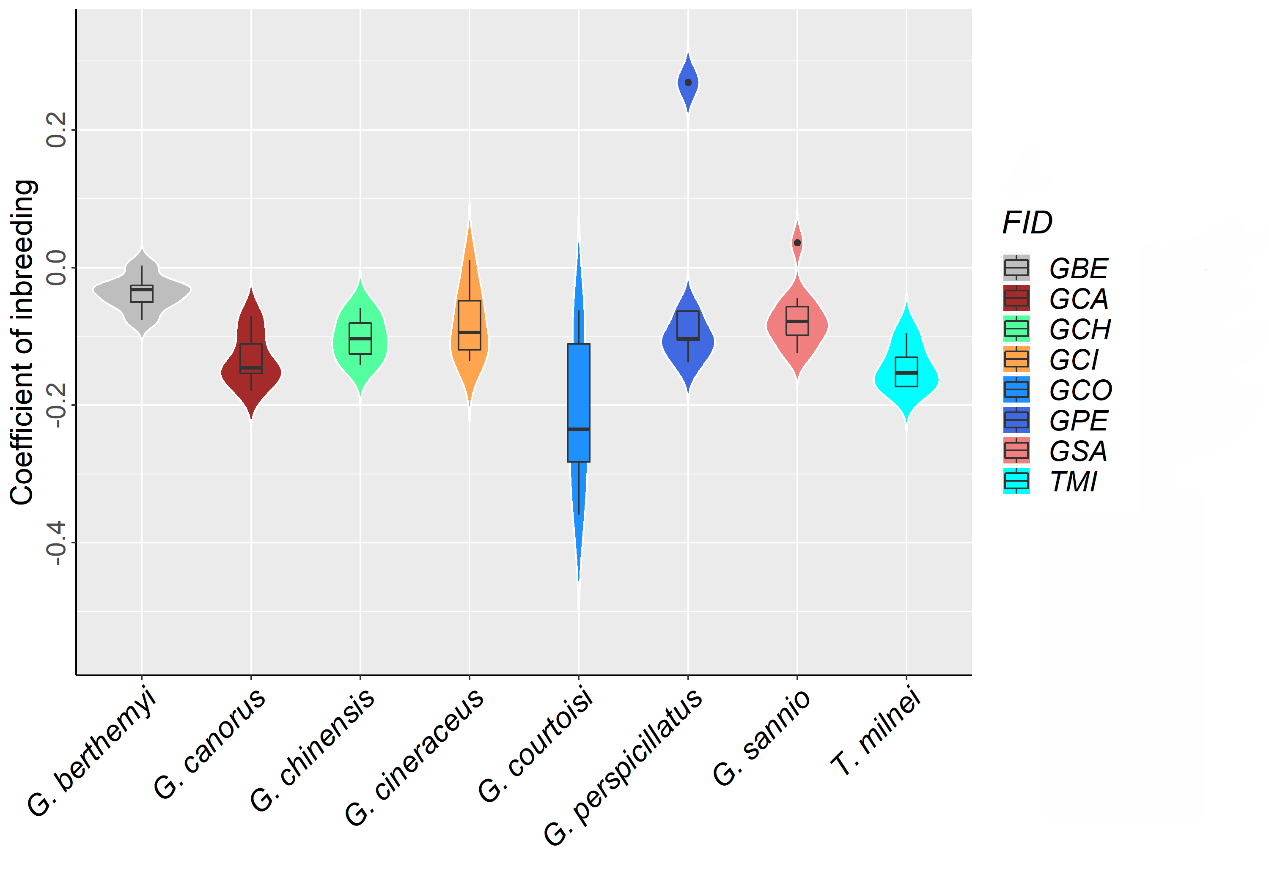
**

**Fig. S18. Distribution of inbreeding coefficient (*F*) in eight *Garrulax* species.**

**
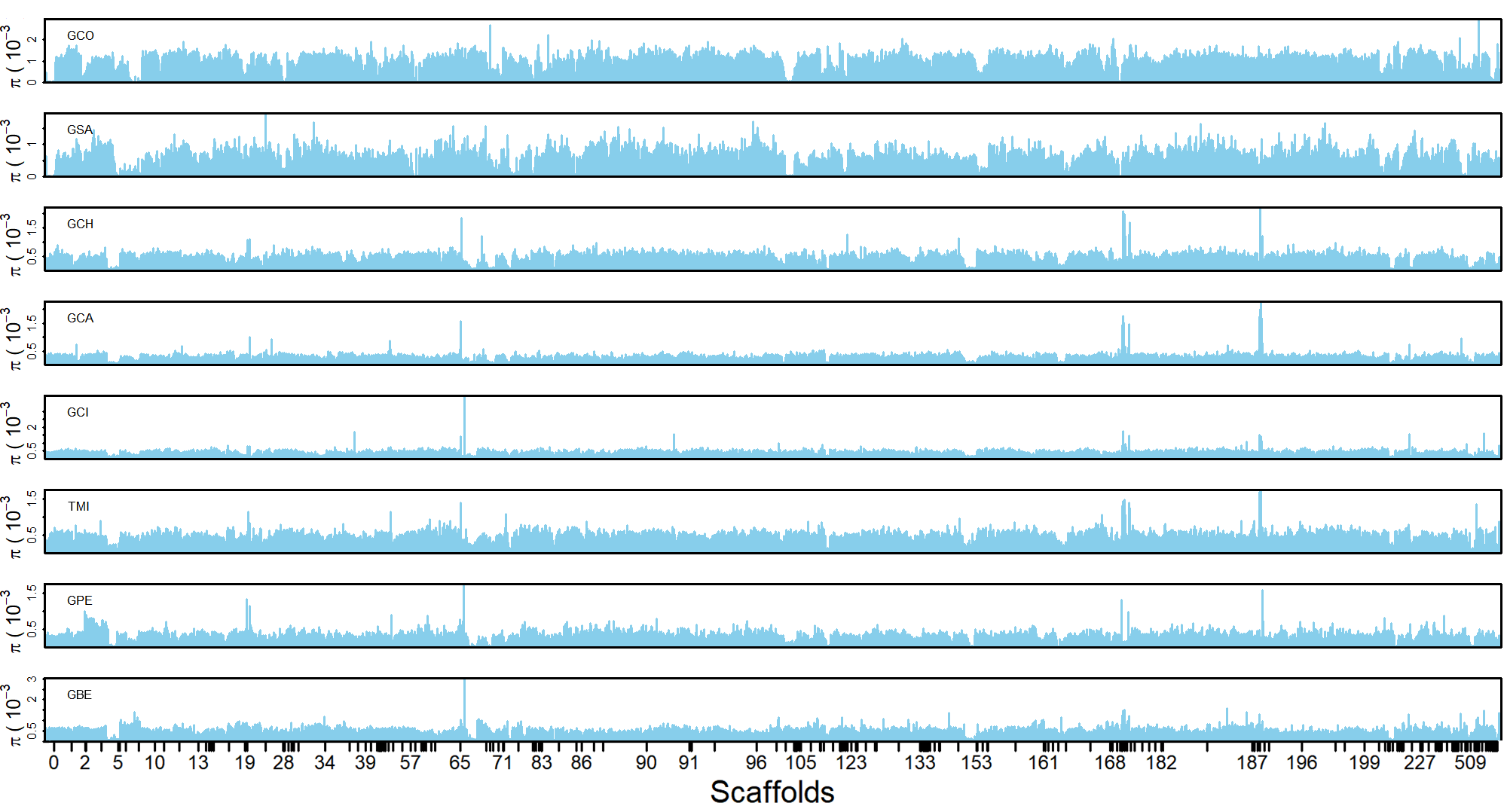
**

**Fig. S19. Distribution of** π **value** **for the eight *Garrulax* species.**

**
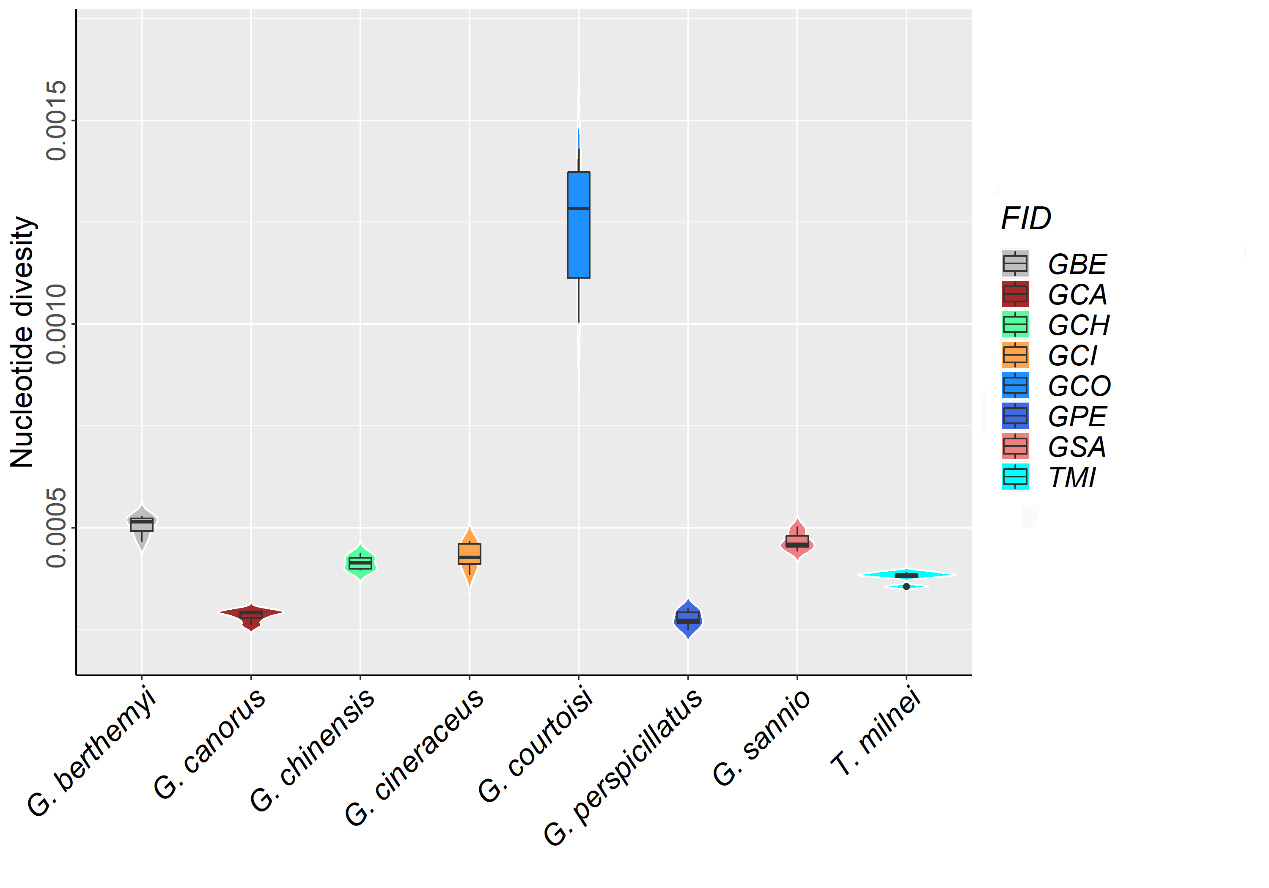
**

**Fig. S20. The scope of** π **value** **for eight *Garrulax* species.**

**
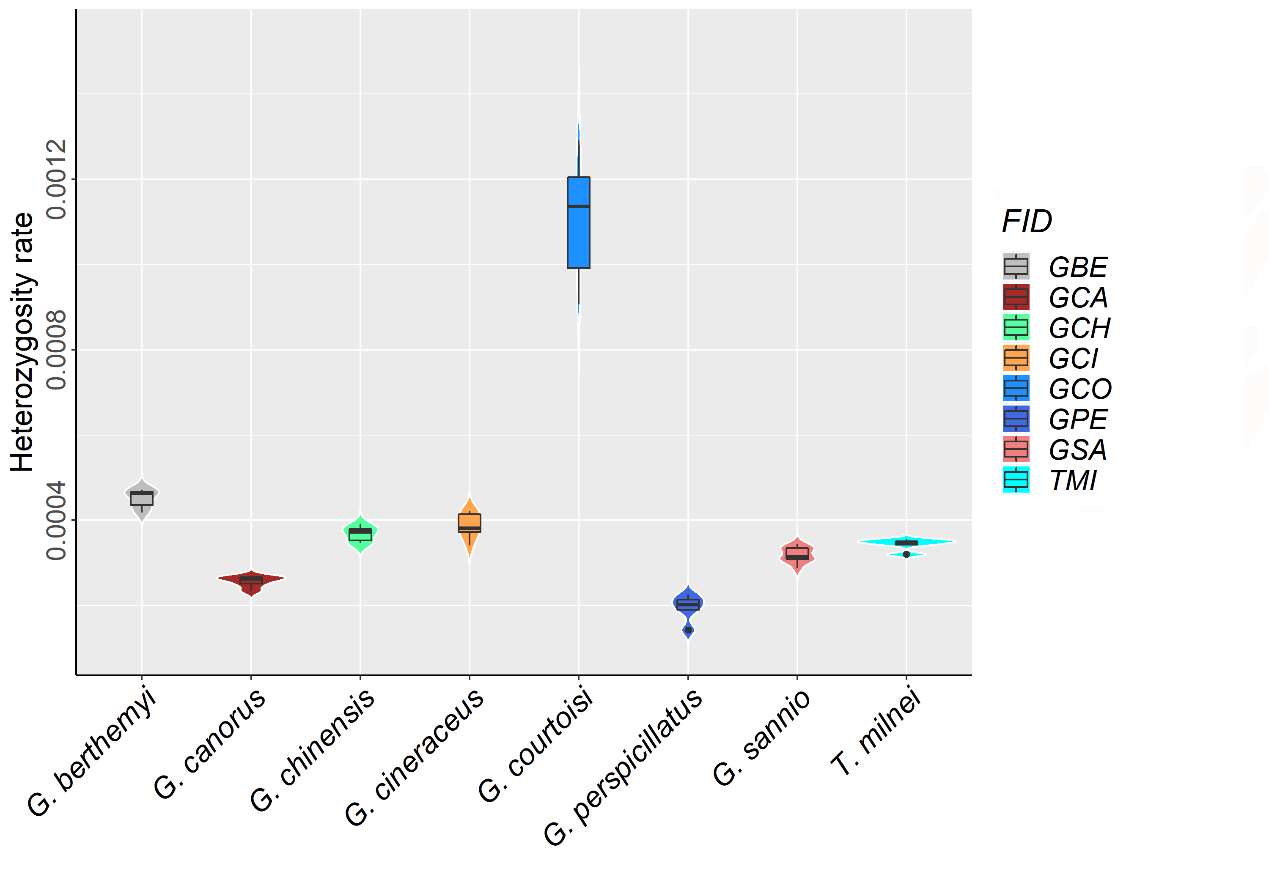
**

**Fig. S21. The scope of heterozygosity rate** **value** **for eight *Garrulax* species.**

**
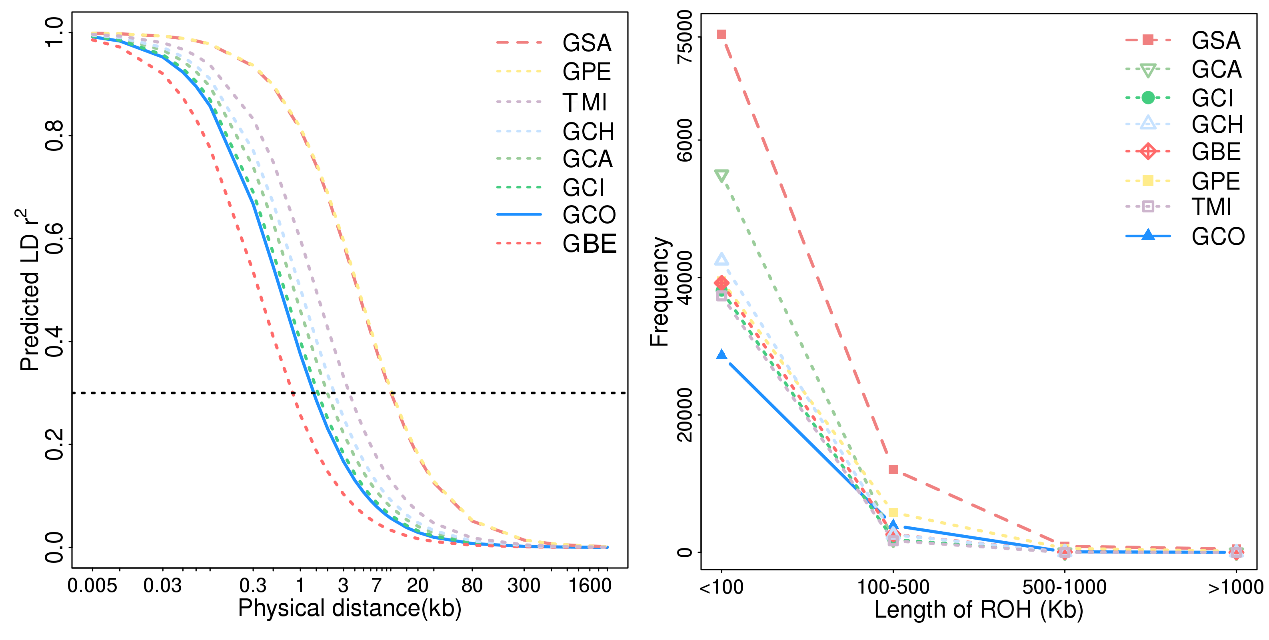
**

**Fig. S22. LD pattern (left) and ROH number (right) for each *Garrulax* species based on *GSA* genome.**

**
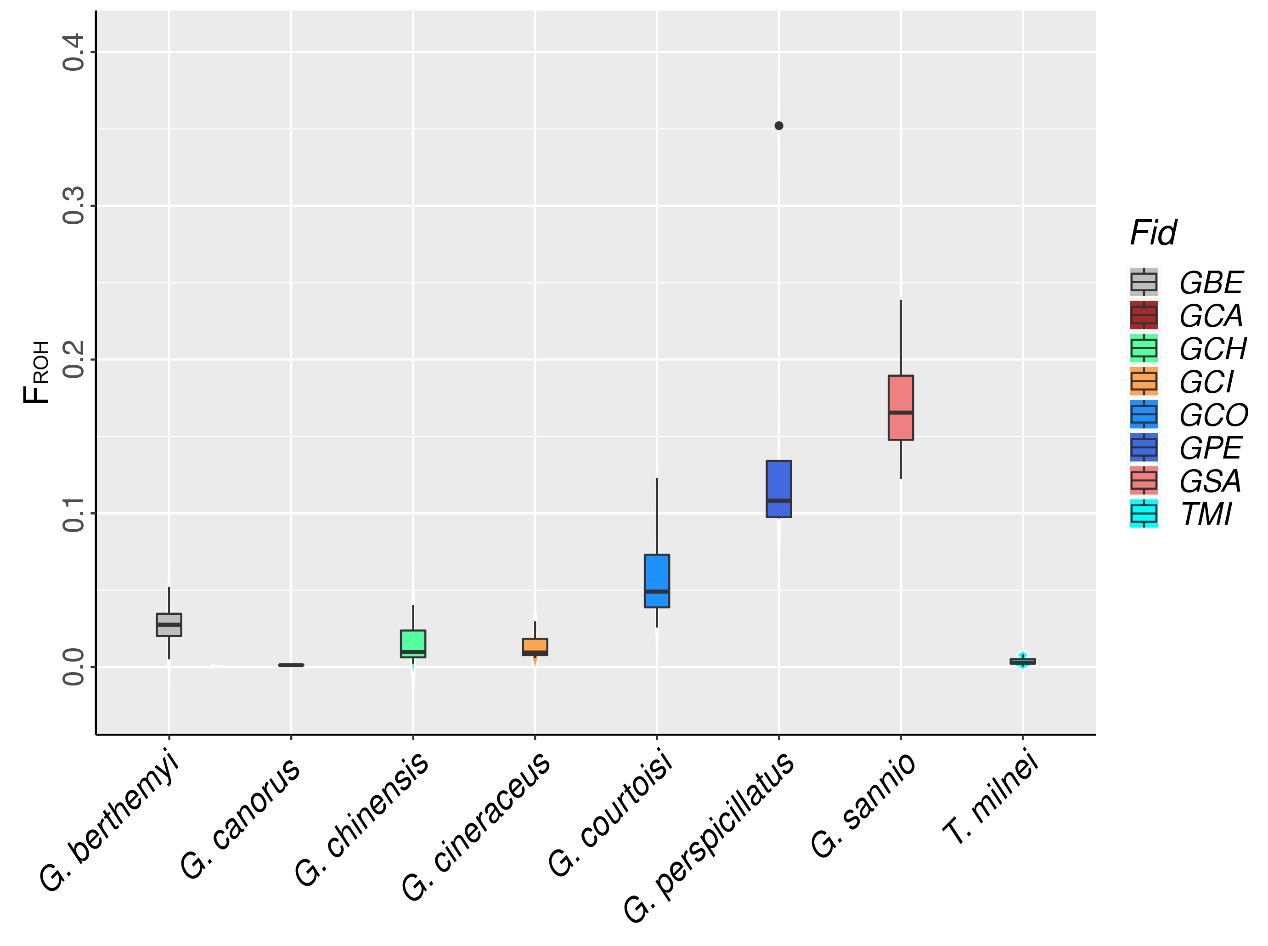
**

**Fig. S23. The scope of F_ROH_** **for eight *Garrulax* species.**

**
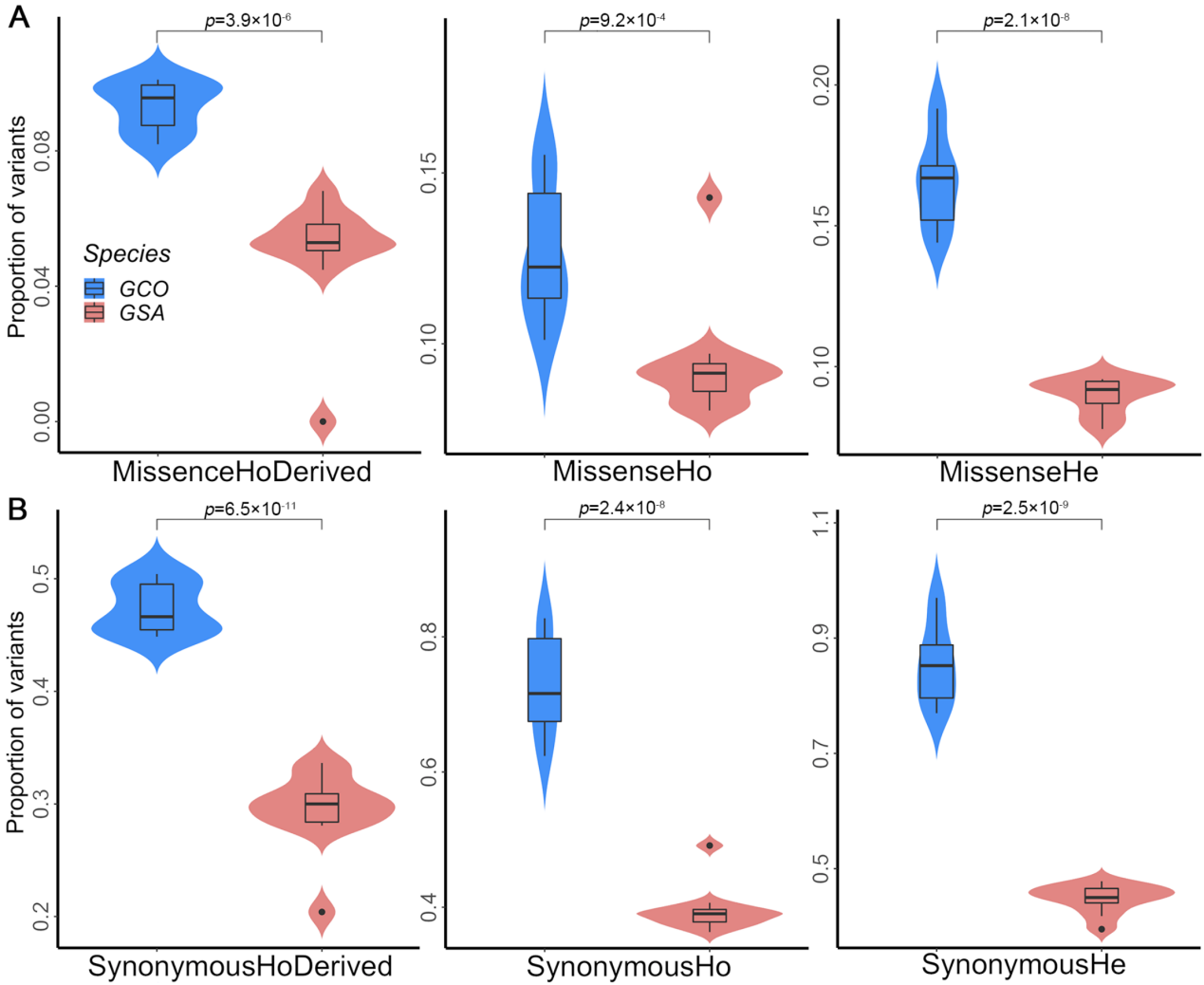
**

**Fig. S24. Comparison missense and synonymous mutations between *GCO* and *GSA*.** The MissenceHoDerived, MissenceHo, and MissenceHe in (A) represent missence mutations of derived homozygote, homozygote, and heterozygote, and respectively. The SynonmousHoDerived, SynonmousHo, and SynonmousHe in (B) represent synonmous mutations of derived homozygote, homozygote, and heterozygote, respectively.

**
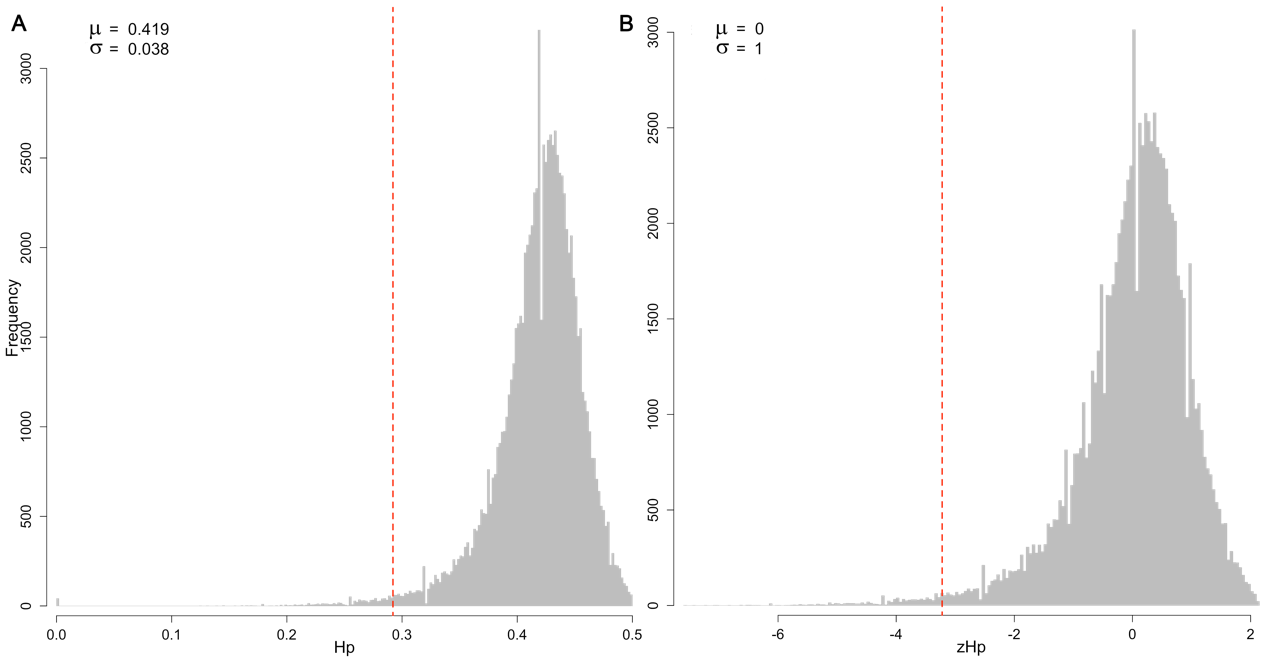
**

**Fig. S25. Distributions of heterozygosity Hp (A) and zHp (B).** Red dotted line means threshold value of top 1% windows.

# Supplementary tables

**Table S1. Summary of the sequencing data for the *de novo* genomes.**

| **Species** | **Platform** | **Insert size** | **Total Data (Gb)** | **Read Length (bp)** | **Sequence Depth (×)** |
| --- | --- | --- | --- | --- | --- |
| **Blue-crowned laughingthrush**  **(*Garrulax courtoisi*, *GCO*)** | Illumina reads | 350 | 134.10 | 150 | 119.00 |
|  | Pacbio reads | - | 113.43 | - | 101.21 |
|  | 10X Genomics | - | 111.90 | - | 99.90 |
|  | Total | - | 359.43 | - | 320.11 |
| **White-browed laughingthrush**  **(*Garrulax sannio*, *GSA*)** | Illumina reads | 350 | 113.17 | 150 | 95.44 |
|  | Pacbio reads | - | 115.92 | - | 97.76 |
|  | 10X Genomics | - | 122.51 | - | 103.32 |
|  | Total | - | 351.60 | - | 296.52 |

**Table S2. Assembly statistics of** **the *GCO* and *GSA* genomes.**

|  | | **Length** | | **Number** | |
| --- | --- | --- | --- | --- | --- |
|  |  | **Contig (bp)** | **Scaffold(bp)** | **Contig** | **Scaffold** |
| **Blue-crowned laughingthrush**  **(*Garrulax*** ***courtoisi*, *GCO*)** | Total | 1,211,308,556 | 1,220,110,490 | 3,080 | 1,708 |
|  | Max | 30,205,013 | 65,042,621 | - | - |
|  | Number>=2000 | - | - | 3,005 | 1,633 |
|  | N50 | 5,499,984 | 11,258,674 | 57 | 27 |
|  | N60 | 3,314,408 | 8,017,530 | 84 | 40 |
|  | N70 | 1,759,696 | 5,292,091 | 135 | 58 |
|  | N80 | 878,283 | 2,513,693 | 230 | 92 |
|  | N90 | 209,555 | 965,872 | 519 | 171 |
| **White-browed laughingthrush**  **(*Garrulax sannio*, *GSA*)** | Total | 1,126,975,557 | 1,132,130,294 | 1,584 | 786 |
|  | Max | 31,778,539 | 67,657,964 | - | - |
|  | Number>=2000 | - | - | 1,541 | 743 |
|  | N50 | 8,312,805 | 22,710,109 | 40 | 16 |
|  | N60 | 5,755,158 | 13,699,823 | 57 | 22 |
|  | N70 | 3,822,692 | 10,489,366 | 80 | 31 |
|  | N80 | 1,816,928 | 6,201,850 | 123 | 45 |
|  | N90 | 652,838 | 2,999,286 | 223 | 69 |

**Table S3. Base contents of the assembled genomes.**

|  | **Genotype** | **Number (bp)** | **% of genome** |
| --- | --- | --- | --- |
| **Blue-crowned laughingthrush**  **(*Garrulax courtoisi*, *GCO*)** | A | 345,266,821 | 28.3 |
|  | T | 345,171,322 | 28.29 |
|  | G | 260,480,304 | 21.35 |
|  | C | 260,390,109 | 21.34 |
|  | N | 8,801,934 | 0.72 |
|  | Total (bp) | 1,220,110,490 | 100 |
|  | GC | 520,870,413 | 43 |
| **White-browed laughingthrush**  **(*Garrulax sannio*, *GSA*)** | A | 323,377,280 | 29 |
|  | T | 323,657,868 | 29 |
|  | G | 239,881,866 | 21 |
|  | C | 240,058,543 | 21 |
|  | N | 5,154,737 | 0 |
|  | Total (bp) | 1,132,130,294 | 100 |
|  | GC | 479,940,409 | 43 |

**Table S4. Read coverage of genome assemblies.**

|  | **Blue-crowned laughingthrush** | **White-browed laughingthrush** |
| --- | --- | --- |
| Average sequencing depth (×)^1^ | 89.17 | 158 |
| Mapping rate (%)^2^ | 98.24 | 97.35 |
| Coverage (%)^3^ | 99.36 | 99.71 |
| Coverage at least 4× (%)^4^ | 99.05 | 99.65 |
| Coverage at least 10× (%) | 98.51 | 99.56 |
| Coverage at least 20× (%) | 97.27 | 99.42 |
| Heterozygosis SNP | 5,122,774 (0.43%) | 1,269,656 (0.12%) |
| Homology SNP^5^ | 9,934 (0.0008%) | 3,059 (0.0003%) |

^1^ average depth of each base pair on the genome.

^2^ rates of short reads aligned to the assembled genomes.

^3^ the proportion of genome covered by reads.

^4^ the proportion of genome covered by 4× reads.

^5^ the number of homozygous SNPs, which reflects the accuracy of genome assembly. The smaller the ratio, the higher the accuracy.

**Table S5. The completeness of the two assembled genomes was assessed by CEGMA and BUSCO.**

| **Species** | CEGMA | | | | | BUSCO | | |
| --- | --- | --- | --- | --- | --- | --- | --- | --- |
|  | Complete^1^ | | Complete + Partial^2^ | | | Complete^5^ | Fragmented^6^ | Missing^7^ |
|  | Prots^3^ | %completeness^4^ | | Prots | %completeness |  |  |  |
| **Blue-crowned laughingthrush** | 149 | 60.08 | | 210 | 84.68 | 96.50% | 2.00% | 1.50% |
| **White-browed laughingthrush** | 151 | 60.89 | | 214 | 86.29 | 97.50% | 1.30% | 1.20% |

^1^ the number and proportion of core genes that assembled through 248 Core Eukaryotic Genes.

^2^ complete and partial core gene.

^3^ the number of assembled core gene.

^4^ the proportion of assembled core gene in 248 Core Eukaryotic Genes.

^5^ complete Single-Copy BUSCOs.

^6^ fragmented BUSCOs.

^7^ missing BUSCOs.

**Table S6. Prediction of repeat elements in the two assembled genomes.**

| **Type of repeat sequences** | **Blue-crowned laughingthrush** | | **White-browed laughingthrush** | |
| --- | --- | --- | --- | --- |
|  | **Repeat Size (bp)** | **% of genome** | **Repeat Size (bp)** | **% of genome** |
| TRF | 48,634,495 | 3.99 | 34,084,816 | 3.01 |
| RepeatMasker | 198,853,745 | 16.3 | 164,042,927 | 14.49 |
| RepeatProteinMask | 50,188,383 | 4.11 | 47,349,611 | 4.18 |
| Total | 228,680,105 | 18.74 | 187,386,234 | 16.55 |

**Table S7. Categories of repeat elements.**

**Table S7a. Categories of repeat elements in *GCO* genome.**

| **TE type** | **Denovo+Repbase** | | **TE Proteins** | | | **Combined TEs** | |
| --- | --- | --- | --- | --- | --- | --- | --- |
|  | **Length(bp)** | **% in Genome** | **Length(bp)** | **% in Genome** | | **Length(bp)** | **% in Genome** |
| DNA | 4,475,060 | 0.37 | 172,824 | | 0.01 | 4,629,335 | 0.38 |
| LINE | 83,665,006 | 6.86 | 24,206,381 | | 1.98 | 86,582,404 | 7.1 |
| SINE | 336,400 | 0.03 | 0 | | 0 | 336,400 | 0.03 |
| LTR | 101,326,788 | 8.3 | 25,831,923 | | 2.12 | 102,579,045 | 8.41 |
| Unknown | 13,595,348 | 1.11 | 0 | | 0 | 13,595,348 | 1.11 |
| Total | 198,853,745 | 16.3 | 50,188,383 | | 4.11 | 201,510,418 | 16.52 |

Note: **Denovo+Repbase** is a library predicted by RepeatModeler, RepeatScout and LTR_FINDER software combined with RepBase nucleotide library, using Uclust software to integrate according to the 80-80-80 principle, and then using RepeatMasker software to identify TEs; **TE Proteins** are based on RepBase protein library, using RepeatProteinMask software to identify TEs; **Combined TEs** is the result of integrating the above two methods and removing redundancy. **Unknown** indicates that the repeat sequence cannot be classified by RepeatMasker.

**Table S7b. Categories of repeat elements in *GSA* genome.**

| **TE type** | **Denovo+Repbase** | | **TE Proteins** | | | **Combined TEs** | |
| --- | --- | --- | --- | --- | --- | --- | --- |
|  | **Length(bp)** | **% in Genome** | **Length(bp)** | **% in Genome** | | **Length(bp)** | **% in Genome** |
| **DNA** | 1,967,296 | 0.17 | 155,583 | | 0.01 | 2,107,878 | 0.19 |
| **LINE** | 67,479,449 | 5.96 | 22,717,420 | | 2.01 | 71,188,297 | 6.29 |
| **SINE** | 163,886 | 0.01 | 0 | | 0 | 163,886 | 0.01 |
| **LTR** | 87,324,650 | 7.71 | 24,491,714 | | 2.16 | 88,633,183 | 7.83 |
| **Unknown** | 11,499,327 | 1.02 | 0 | | 0 | 11,499,327 | 1.02 |
| **Total** | 164,042,927 | 14.49 | 47,349,611 | | 4.18 | 167,342,119 | 14.78 |

Note: **Denovo+Repbase** is a library predicted by RepeatModeler, RepeatScout and LTR_FINDER software combined with RepBase nucleotide library, using Uclust software to integrate according to the 80-80-80 principle, and then using RepeatMasker software to identify TEs; **TE Proteins** are based on RepBase protein library, using RepeatProteinMask software to identify TEs; **Combined TEs** is the result of integrating the above two methods and removing redundancy. **Unknown** indicates that the repeat sequence cannot be classified by RepeatMasker.

**Table S8. Prediction of gene structure in two genomes.**

**Table S8a. Prediction of gene structure *GCO* genome.**

| **Gene set** | | **Number** | **Average gene length (bp)** | **Average CDS length (bp)** | **Average exons per gene** | **Average exon length (bp)** | **Average intron length (bp)** |
| --- | --- | --- | --- | --- | --- | --- | --- |
| De novo | Augustus | 22,225 | 14,408.15 | 1,251.34 | 6.85 | 182.68 | 2,249.14 |
|  | GlimmerHMM | 206,906 | 4,993.00 | 442.44 | 2.5 | 176.82 | 3,029.10 |
|  | SNAP | 80,976 | 20,435.79 | 681.44 | 4.62 | 147.48 | 5,455.94 |
|  | Geneid | 28,081 | 28,280.13 | 1,286.95 | 6.87 | 187.43 | 4,601.39 |
|  | Genscan | 36,217 | 24,423.74 | 1,358.91 | 8.05 | 168.76 | 3,270.57 |
| Homolog | African ostrich | 21,576 | 14,017.96 | 1,263.53 | 6.6 | 191.49 | 2,278.17 |
|  | Crested ibis | 25,574 | 12,432.49 | 1,093.87 | 6.07 | 180.15 | 2,235.54 |
|  | Peking duck | 20,644 | 15,606.24 | 1,380.28 | 7.02 | 196.5 | 2,361.37 |
|  | Red junglefowl | 25,523 | 13,125.72 | 1,201.26 | 6.15 | 195.3 | 2,315.03 |
|  | Turkey | 28,495 | 9,823.63 | 1,026.10 | 5.37 | 191.18 | 2,014.41 |
|  | Zhedong white goose | 30,982 | 10,053.57 | 1,026.51 | 5.16 | 198.83 | 2,168.57 |
| RNA-seq | PASA | 955 | 13,568.84 | 1,073.25 | 7.41 | 144.91 | 1,950.52 |
| EVM | | 25,576 | 13,745.15 | 1,178.06 | 6.7 | 175.81 | 2,204.45 |
| PASA-update | | 25,527 | 14,033.73 | 1,182.46 | 6.73 | 175.68 | 2,242.51 |
| Final set | | 20,819 | 16,666.61 | 1,352.71 | 7.86 | 172.06 | 2,231.79 |

**Table S8b. Prediction of gene structure in *GSA* genome.**

| **Gene set** | | **Number** | **Average gene length (bp)** | **Average CDS length (bp)** | **Average exons per gene** | **Average exon length (bp)** | **Average intron length (bp)** |
| --- | --- | --- | --- | --- | --- | --- | --- |
| De novo | Augustus | 19,432 | 14,778.39 | 1,331.70 | 7.39 | 180.14 | 2,103.43 |
|  | GlimmerHMM | 172,820 | 5,667.67 | 473.26 | 2.8 | 169.18 | 2,890.02 |
|  | SNAP | 63,399 | 22,613.25 | 697.2 | 4.79 | 145.7 | 5,789.83 |
|  | Geneid | 25,603 | 29,274.32 | 1,331.64 | 7.04 | 189.17 | 4,626.87 |
|  | Genscan | 33,589 | 24,960.82 | 1,383.55 | 8.14 | 169.93 | 3,301.32 |
| Homolog | African ostrich | 19,946 | 15,255.48 | 1,336.63 | 6.98 | 191.39 | 2,326.05 |
|  | Crested ibis | 25,008 | 12,660.59 | 1,120.07 | 6.14 | 182.29 | 2,243.30 |
|  | Peking duck | 18,613 | 17,277.47 | 1,491.15 | 7.56 | 197.14 | 2,405.00 |
|  | Red junglefowl | 24,151 | 13,788.63 | 1,234.16 | 6.37 | 193.67 | 2,336.77 |
|  | Turkey | 27,183 | 10,273.62 | 1,081.08 | 5.5 | 196.51 | 2,042.20 |
|  | Zhedong white goose | 30,399 | 10,294.18 | 1,061.24 | 5.22 | 203.18 | 2,186.23 |
| RNA-seq | PASA | 1,471 | 15,694.07 | 1,252.77 | 8.82 | 142.02 | 1,846.43 |
| EVM | | 23,158 | 14,723.40 | 1,240.72 | 7.03 | 176.55 | 2,236.76 |
| PASA-update | | 23,084 | 15,196.12 | 1,248.74 | 7.08 | 176.3 | 2,292.86 |
| Final set | | 18,367 | 18,508.14 | 1,466.69 | 8.48 | 172.9 | 2,277.44 |

**Table S9. Gene structure of genomes of *Garrulax* species and other avians.**

| **Species** | **Number** | **Average gene length (bp)** | **Average CDS length (bp)** | **Average exon length (bp)** | **Average intron length (bp)** | **Average exons per genes** |
| --- | --- | --- | --- | --- | --- | --- |
| Blue-crowned laughingthrush | 20,819 | 16,666.61 | 1,352.71 | 7.86 | 172.06 | 2,231.79 |
| White-browed laughingthrush | 18,367 | 18,508.14 | 1,466.69 | 8.48 | 172.9 | 2,277.44 |
| Zedong white goose | 14,696 | 30,179.53 | 1,763.51 | 10.85 | 162.58 | 2,885.76 |
| Red junglefowl | 17,472 | 25,716.94 | 1,733.83 | 10.18 | 170.34 | 2,612.91 |
| Peking duck | 15,735 | 29,343.95 | 1,775.40 | 10.62 | 167.19 | 2,865.99 |
| Turkey | 17,968 | 18,802.05 | 1,493.86 | 9.02 | 165.64 | 2,158.48 |
| African ostrich | 14,577 | 32,363.09 | 1,756.13 | 10.84 | 161.98 | 3,110.00 |
| Crested ibis | 15,018 | 30,359.98 | 1,766.29 | 10.95 | 161.35 | 2,874.59 |

**Table S10. Functional annotation of the predicted protein-coding genes in the *Garrulax* genome assemblies**

|  | **Blue-crowned laughingthrush** | | **White-browed laughingthrush** | |
| --- | --- | --- | --- | --- |
|  | **Number** | **Percent (%)** | **Number** | **Percent (%)** |
| Total | 20,819 | - | 18,367 | - |
| Swissprot | 17,989 | 86.4 | 15,874 | 86.4 |
| NR | 18,956 | 91.1 | 16,648 | 90.6 |
| KEGG | 16,421 | 78.9 | 14,477 | 78.8 |
| InterPro | 17,484 | 84 | 15,478 | 84.3 |
| GO | 12,513 | 60.1 | 11,380 | 62 |
| Pfam | 15,227 | 73.1 | 13,831 | 75.3 |
| Annotated | 19,037 | 91.4 | 16,699 | 90.9 |
| Unannotated | 1,782 | 8.6 | 1,668 | 9.1 |

**Table S11. Statistics of noncoding RNAs of the *GCO* and *GSA* genomes.**

| **Type** | | **Blue-crowned laughingthrush** | | **White-browed laughingthrush** | |
| --- | --- | --- | --- | --- | --- |
|  |  | **Copy number** | **Total length (bp)** | **Copy number** | **Total length (bp)** |
| miRNA | | 341 | 29,417 | 322 | 27,598 |
| tRNA | | 369 | 27,594 | 344 | 25,751 |
| rRNA | rRNA | 133 | 21,437 | 220 | 82,574 |
|  | 18S | 1 | 48 | 52 | 32,129 |
|  | 28S | 42 | 10,964 | 147 | 47,413 |
|  | 5.8S | 2 | 302 | 18 | 2,782 |
|  | 5S | 88 | 10,123 | 3 | 250 |
| snRNA | snRNA | 261 | 31,429 | 268 | 31,019 |
|  | CD-box | 123 | 11,532 | 132 | 11,815 |
|  | HACA-box | 78 | 11,271 | 76 | 10,705 |
|  | splicing | 42 | 5,516 | 43 | 5,596 |

**Table S12. Positive selection genes (PSGs) identified in *GCO* and *GSA*.**

| **Blue-crowned laughingthrush** | **White-browed laughingthrush** | **Gene** |
| --- | --- | --- |
| evm.model.scaffold_1552.20 | evm.model.scaffold_10.27 | *Nqo2* |
| evm.model.scaffold_1552.42 | evm.model.scaffold_10.50 | *LY86* |
| evm.model.scaffold_1552.149 | evm.model.scaffold_10.151 | *CDKAL1* |
| evm.model.scaffold_356.276 | evm.model.scaffold_106.402 | *CCL13* |
| evm.model.scaffold_221.31 | evm.model.scaffold_114.115 | *Pold4* |
| evm.model.scaffold_51.455 | evm.model.scaffold_117.10 | *Hcn4* |
| evm.model.scaffold_122.3 | evm.model.scaffold_12.24 | *PODXL* |
| evm.model.scaffold_265.41 | evm.model.scaffold_120.27 | *ST8SIA4* |
| evm.model.scaffold_152.51 | evm.model.scaffold_126.10 | *FSIP2* |
| evm.model.scaffold_1603.3 | evm.model.scaffold_13.5 | *RMI2* |
| evm.model.scaffold_207.109 | evm.model.scaffold_135.13 | *IL22RA1* |
| evm.model.scaffold_46.26 | evm.model.scaffold_14.59 | *Ptger2* |
| evm.model.scaffold_1443.9 | evm.model.scaffold_144.7 | *XPO7* |
| evm.model.scaffold_0.32 | evm.model.scaffold_148.101 | *UBXN10* |
| evm.model.scaffold_92.23 | evm.model.scaffold_148.321 | *SCNN1A* |
| evm.model.scaffold_268.22 | evm.model.scaffold_152.489 | *CA4* |
| evm.model.scaffold_51.304 | evm.model.scaffold_152.344 | *C15orf61* |
| evm.model.scaffold_243.153 | evm.model.scaffold_155.20 | *AAED1* |
| evm.model.scaffold_207.49 | evm.model.scaffold_157.118 | *KLF5* |
| evm.model.scaffold_380.55 | evm.model.scaffold_16.9 | *TFB2M* |
| evm.model.scaffold_1591.66 | evm.model.scaffold_160.404 | *IL4R* |
| evm.model.scaffold_335.121 | evm.model.scaffold_165.34 | *sepp1b* |
| evm.model.scaffold_335.62 | evm.model.scaffold_167.249 | *Tmem125* |
| evm.model.scaffold_335.17 | evm.model.scaffold_167.295 | *CTBS* |
| evm.model.scaffold_914.12 | evm.model.scaffold_167.339 | *ZNF92* |
| evm.model.scaffold_344.7 | evm.model.scaffold_170.57 | *Heph* |
| evm.model.scaffold_4.54 | evm.model.scaffold_176.88 | *SAP30BP* |
| evm.model.scaffold_139.100 | evm.model.scaffold_186.991 | *Paqr3* |
| evm.model.scaffold_178.320 | evm.model.scaffold_186.594 | *TMEM154* |
| evm.model.scaffold_198.103 | evm.model.scaffold_186.269 | *PPARGC1A* |
| evm.model.scaffold_299.63 | evm.model.scaffold_186.530 | *TLL1* |
| evm.model.scaffold_71.7 | evm.model.scaffold_186.24 | *Pcgf1* |
| evm.model.scaffold_255.497 | evm.model.scaffold_196.468 | *ASPH* |
| evm.model.scaffold_347.76 | evm.model.scaffold_198.53 | *RAB36* |
| evm.model.scaffold_347.158 | evm.model.scaffold_198.137 | *IGLC1* |
| evm.model.scaffold_243.219 | evm.model.scaffold_201.13 | *HAUS6* |
| evm.model.scaffold_162.24 | evm.model.scaffold_202.123 | *Tspan2* |
| evm.model.scaffold_970.8 | evm.model.scaffold_211.5 | *FABP3* |
| evm.model.scaffold_340.137 | evm.model.scaffold_25.47 | *P2ry2* |
| evm.model.scaffold_351.22 | evm.model.scaffold_25.173 | *CD200R1A* |
| evm.model.scaffold_82.5 | evm.model.scaffold_25.209 | *Lhx8* |
| evm.model.scaffold_1150.42 | evm.model.scaffold_296.47 | *EEF2KMT* |
| evm.model.scaffold_118.84 | evm.model.scaffold_38.78 | *Coprs* |
| evm.model.scaffold_564.47 | evm.model.scaffold_38.198 | *CCDC40* |
| evm.model.scaffold_121.20 | evm.model.scaffold_4.523 | *ATP6V1E1* |
| evm.model.scaffold_331.19 | evm.model.scaffold_4.482 | *Parvb* |
| evm.model.scaffold_331.32 | evm.model.scaffold_4.500 | *Syce3* |
| evm.model.scaffold_386.85 | evm.model.scaffold_4.275 | *Rad51ap1* |
| evm.model.scaffold_52.52 | evm.model.scaffold_40.13 | *UCN3* |
| evm.model.scaffold_73.67 | evm.model.scaffold_509.63 | *SMTNL1* |
| evm.model.scaffold_179.1 | evm.model.scaffold_52.48 | *GNG7* |
| evm.model.scaffold_179.15 | evm.model.scaffold_52.64 | *GZMM* |
| evm.model.scaffold_45.7 | evm.model.scaffold_52.42 | *DDA1* |
| evm.model.scaffold_65.60 | evm.model.scaffold_52.224 | *NFIC* |
| evm.model.scaffold_246.25 | evm.model.scaffold_54.25 | *CAV3* |
| evm.model.scaffold_216.44 | evm.model.scaffold_61.41 | *Fam72a* |
| evm.model.scaffold_509.11 | evm.model.scaffold_634.12 | *PLEKHA7* |
| evm.model.scaffold_223.258 | evm.model.scaffold_65.283 | *Tmem140* |
| evm.model.scaffold_206.412 | evm.model.scaffold_65.288 | *NGB* |
| evm.model.scaffold_206.406 | evm.model.scaffold_65.339 | *LRRC74A* |
| evm.model.scaffold_206.352 | evm.model.scaffold_65.341 | *Fam161b* |
| evm.model.scaffold_206.350 | evm.model.scaffold_65.504 | *FBXO33* |
| evm.model.scaffold_206.197 | evm.model.scaffold_7.94 | *Ttc9* |
| evm.model.scaffold_319.26 | evm.model.scaffold_7.55 | *GNG10* |
| evm.model.scaffold_124.36 | evm.model.scaffold_77.241 | *APOC3* |
| evm.model.scaffold_124.10 | evm.model.scaffold_77.258 | *CD3E* |
| evm.model.scaffold_1280.24 | evm.model.scaffold_77.280 | *Rpl37* |
| evm.model.scaffold_303.3 | evm.model.scaffold_77.193 | *nr4a1* |
| evm.model.scaffold_223.282 | evm.model.scaffold_84.16 | *IKBIP* |
| evm.model.scaffold_223.442 | evm.model.scaffold_84.17 | *LLPH* |
| evm.model.scaffold_315.39 | evm.model.scaffold_84.181 | *DBX2* |
| evm.model.scaffold_236.17 | evm.model.scaffold_84.237 | *SLC31A2* |
| evm.model.scaffold_360.431 | evm.model.scaffold_86.204 | *NUDT15* |
| evm.model.scaffold_360.537 | evm.model.scaffold_90.220 | *BRCA2* |
| evm.model.scaffold_360.557 | evm.model.scaffold_90.313 | *POMP* |
| evm.model.scaffold_86.182 | evm.model.scaffold_90.333 | *Mfap5* |
| evm.model.scaffold_86.173 | evm.model.scaffold_90.591 | *c3ar1* |
| evm.model.scaffold_94.77 | evm.model.scaffold_90.600 | *CD86* |
| evm.model.scaffold_58.216 | evm.model.scaffold_90.615 | *Hoxd10* |
| evm.model.scaffold_58.483 | evm.model.scaffold_95.270 | *Muc13* |
| evm.model.scaffold_1396.190 | evm.model.scaffold_95.520 | *Leg1* |
| evm.model.scaffold_1396.28 | evm.model.scaffold_96.22 | *CCNC* |
| evm.model.scaffold_901.19 | evm.model.scaffold_96.188 | *Fbxo21* |
| evm.model.scaffold_237.452 | evm.model.scaffold_98.50 | *PROCR* |
| evm.model.scaffold_237.450 | evm.model.scaffold_98.52 | *C21orf2* |

**Table S13. The information of 25 species download from NCBI database.**

| **Species** | **Name** | **Abbreviation** |
| --- | --- | --- |
| *Aquila chrysaetos* | Golden eagle | ACH |
| *Acanthisitta chloris* | Rifleman | ACS |
| *Anser cygnoides* | Swan goose | ACY |
| *Anas platyrhynchos* | Mallard | APS |
| *Coturnix japonica* | Japanese Quail | CJA |
| *Columba livia* | Rock pigeon | CLA |
| *Charadrius vociferus* | Killdeer | CVS |
| *Dromaius novaehollandiae* | Emu | DNE |
| *Egretta garzetta* | Little egret | EGA |
| *Ficedula albicollis* | Collared flycatcher | FAS |
| *Falco cherrug* | Saker falcon | FCG |
| *Falco peregrinus* | Peregrine falcon | FPS |
| *Geospiza fortis* | Medium ground-finch | GFS |
| *Gallus gallus* | Red junglefowl | GGS |
| *Meleagris gallopavo* | Turkey | MGO |
| *Melopsittacus undulatus* | Budgerigar | MUS |
| *Manacus vitellinus* | Golden-collared manakin | MVS |
| *Nipponia nippon* | Crested Ibis | NNN |
| *Opisthocomus hoazin* | Hoatzin | OHN |
| *Pygoscelis adeliae* | Adelie penguin | PAE |
| *Pygosceli antarcticus* | Chinstrap penguin | PAS |
| *Thalassarche chlororhynchos* | Atlantic yellow-nosed albatross | TCS |
| *Struthio camelus* | Common ostrich | SCS |
| *Strigops habroptila* | Kakapo | SHA |
| *Taeniopygia guttata* | Zebra finch | TGA |

**Table S14. Expanded and contracted gene families identified by CAFE.**

| **Name** | **No. of**  **expanded families** | **No. of**  **contracted families** | **Rapidly evolving families** |
| --- | --- | --- | --- |
| Golden eagle | 412 (145) | 2900 (0) | 17 |
| Rifleman | 1019 (0) | 4427 (0) | 7 |
| Swan goose | 216 (19) | 1248 (55) | 24 |
| Mallard | 541 (307) | 1281 (13) | 42 |
| Japanese quail | 247 (27) | 1469 (98) | 18 |
| Rock pigeon | 393 (95) | 2293 (0) | 12 |
| Killdeer | 322 (0) | 442 (0) | 1 |
| Emu | 276 (7) | 580 (0) | 5 |
| Little egret | 268 (0) | 1435 (0) | 1 |
| Collared flycatcher | 401 (50) | 1547 (0) | 10 |
| Saker falcon | 137 (4) | 453 (6) | 7 |
| Peregrine falcon | 177 (5) | 327 (0) | 4 |
| Medium ground-finch | 289 (19) | 1201 (17) | 14 |
| Red junglefowl | 282 (482) | 1978 (6) | 47 |
| Turkey | 1216 (88) | 1226 (17) | 22 |
| Budgerigar | 649 (228) | 472 (4) | 15 |
| Golden-collared manakin | 311 (86) | 4606 (0) | 11 |
| Crested Ibis | 417 (0) | 702 (0) | 1 |
| Hoatzin | 189 (34) | 1360 (0) | 6 |
| Adelie penguin | 154 (3) | 1753 (30) | 16 |
| Chinstrap penguin | 667 (136) | 1406 (5) | 35 |
| Atlantic yellow-nosed albatross | 583 (52) | 4361 (0) | 12 |
| Common ostrich | 306 (0) | 1466 (0) | 1 |
| Kakapo | 273 (19) | 1495 (14) | 6 |
| Zebra finch | 323 (235) | 802 (4) | 25 |
| Blue-crowned laughingthrush | 1698 (10) | 1114 (19) | 12 |
| White-browed Laughingthrush | 542 (3) | 890 (3) | 8 |

**Table S15. Sequencing data quality of 66 individuals.**

| **ID** | **Raw Base(bp)** | **Clean Base(bp)** | **Effective Rate(%)** | **Q20(%)** | **GC Content(%)** | **Depth(×)** |
| --- | --- | --- | --- | --- | --- | --- |
| GSA1 | 21,564,341,400 | 21,477,945,000 | 99.60 | 96.40 | 42.31 | 17.5844 |
| GSA2 | 30,667,441,200 | 30,491,949,900 | 99.43 | 97.16 | 43.55 | 23.7258 |
| GSA3 | 28,475,031,300 | 28,406,121,300 | 99.76 | 97.22 | 43.33 | 22.1676 |
| GSA4 | 28,141,348,500 | 28,058,433,600 | 99.71 | 97.62 | 43.20 | 21.7125 |
| GSA5 | 32,481,826,500 | 32,376,627,600 | 99.68 | 97.32 | 43.36 | 24.9324 |
| GSA6 | 27,981,904,200 | 27,924,353,700 | 99.79 | 97.48 | 43.39 | 22.0789 |
| GSA7 | 23,964,924,900 | 23,889,715,800 | 99.69 | 96.86 | 43.25 | 18.1135 |
| GSA8 | 29,065,955,700 | 28,937,517,600 | 99.56 | 96.88 | 44.03 | 21.8438 |
| GSA9 | 26,016,020,700 | 25,877,991,600 | 99.47 | 97.48 | 42.66 | 20.4194 |
| GSA10 | 25,844,317,500 | 25,743,735,300 | 99.61 | 97.32 | 42.87 | 20.1332 |
| GSA11 | 26,754,509,700 | 26,643,310,500 | 99.58 | 96.97 | 42.59 | 20.8195 |
| GCO1 | 29,774,341,800 | 29,663,725,200 | 99.63 | 96.97 | 43.16 | 22.6744 |
| GCO2 | 29,840,148,300 | 29,736,146,400 | 99.65 | 97.41 | 43.88 | 22.5212 |
| GCO3 | 24,430,781,100 | 24,357,151,800 | 99.70 | 96.61 | 43.48 | 18.5067 |
| GCO4 | 33,811,727,700 | 33,731,973,300 | 99.76 | 97.01 | 43.51 | 25.7953 |
| GCO5 | 30,353,672,400 | 29,943,331,500 | 98.65 | 95.53 | 44.48 | 20.2357 |
| GCO6 | 29,271,602,700 | 28,941,621,600 | 98.87 | 93.05 | 45.76 | 19.2632 |
| GCO7 | 24,790,853,400 | 24,690,707,700 | 99.60 | 96.28 | 42.94 | 18.7963 |
| GCO8 | 28,189,671,300 | 28,024,545,900 | 99.41 | 95.82 | 43.68 | 20.3563 |
| GCO9 | 30,021,367,800 | 29,874,273,300 | 99.51 | 95.70 | 43.62 | 22.1544 |
| GCO10 | 28,131,177,900 | 28,019,851,200 | 99.60 | 96.29 | 43.45 | 20.8969 |
| GCO11 | 27,973,888,200 | 27,869,475,000 | 99.63 | 96.85 | 43.55 | 21.2017 |
| GCO12 | 35,803,812,900 | 35,660,558,100 | 99.60 | 97.25 | 43.50 | 27.3169 |
| GCO13 | 31,749,856,200 | 31,655,937,600 | 99.70 | 97.51 | 43.84 | 24.258 |
| GCO14 | 31,519,824,600 | 31,358,538,900 | 99.49 | 97.22 | 44.16 | 22.9331 |
| GPO1 | 31,787,812,800 | 31,491,299,400 | 99.07 | 96.61 | 43.81 | 23.0213 |
| GPO2 | 29,367,437,700 | 29,271,254,100 | 99.67 | 95.67 | 43.72 | 22.1096 |
| GPO3 | 28,194,024,300 | 28,043,958,000 | 99.47 | 96.86 | 43.08 | 21.5717 |
| GPO4 | 21,881,385,900 | 21,736,946,400 | 99.34 | 96.51 | 42.04 | 16.9363 |
| GPO5 | 31,078,825,500 | 30,942,726,600 | 99.56 | 97.03 | 43.38 | 23.4465 |
| GPO6 | 27,297,475,800 | 27,185,539,800 | 99.59 | 95.94 | 43.58 | 20.6059 |
| GPO7 | 24,841,031,100 | 24,660,310,800 | 99.27 | 96.60 | 44.12 | 18.1616 |
| GPO8 | 26,970,156,600 | 26,732,473,200 | 99.12 | 97.09 | 42.97 | 20.1787 |
| GMI1 | 32,011,784,700 | 31,923,168,000 | 99.72 | 97.48 | 43.13 | 24.3803 |
| GMI2 | 28,098,711,600 | 28,032,773,400 | 99.77 | 97.19 | 43.28 | 21.7851 |
| GMI3 | 27,809,235,000 | 27,711,120,300 | 99.65 | 97.38 | 43.51 | 21.5948 |
| GMI4 | 25,174,908,900 | 25,088,986,800 | 99.66 | 97.56 | 43.08 | 19.453 |
| GMI5 | 30,610,786,200 | 30,503,714,700 | 99.65 | 97.62 | 43.20 | 23.8155 |
| GMI6 | 29,009,630,400 | 28,915,183,500 | 99.67 | 97.01 | 43.37 | 22.1293 |
| GMI7 | 23,210,618,700 | 23,143,096,500 | 99.71 | 96.78 | 43.22 | 17.327 |
| GMI8 | 26,643,700,500 | 26,565,919,200 | 99.71 | 97.04 | 43.13 | 20.4929 |
| GCA1 | 29,925,791,700 | 29,869,463,400 | 99.81 | 96.95 | 43.20 | 22.9704 |
| GCA2 | 32,780,181,300 | 32,735,631,600 | 99.86 | 97.43 | 42.58 | 25.336 |
| GCA3 | 31,947,538,200 | 31,837,626,000 | 99.66 | 96.94 | 42.91 | 23.9082 |
| GCA4 | 33,072,742,500 | 32,976,330,600 | 99.71 | 97.00 | 42.84 | 24.8795 |
| GCA5 | 36,835,967,700 | 36,723,285,900 | 99.69 | 97.06 | 43.91 | 27.8928 |
| GCA6 | 21,744,932,700 | 21,689,125,500 | 99.74 | 96.45 | 43.33 | 17.1402 |
| GPE1 | 28,195,309,200 | 28,101,200,400 | 99.67 | 96.85 | 42.87 | 21.4869 |
| GPE2 | 28,476,959,100 | 28,366,884,300 | 99.61 | 97.34 | 43.18 | 21.8035 |
| GPE3 | 27,629,826,600 | 27,340,697,100 | 98.95 | 96.35 | 43.75 | 20.1117 |
| GPE4 | 27,404,495,700 | 27,175,472,100 | 99.16 | 96.01 | 43.83 | 19.8277 |
| GPE5 | 25,611,771,300 | 25,531,906,800 | 99.69 | 96.12 | 42.80 | 19.4177 |
| CCY1 | 30,098,153,100 | 30,002,454,300 | 99.68 | 97.27 | 43.79 | 18.9216 |
| GCH1 | 29,963,983,800 | 29,859,612,300 | 99.65 | 97.16 | 44.01 | 23.0015 |
| GCH2 | 32,043,817,800 | 31,947,705,600 | 99.70 | 97.33 | 44.09 | 24.2757 |
| GCH3 | 32,537,681,700 | 32,477,977,800 | 99.82 | 97.37 | 43.64 | 24.8478 |
| GCH4 | 24,819,172,200 | 24,752,604,600 | 99.73 | 97.35 | 43.25 | 19.0177 |
| GCH5 | 27,750,483,900 | 27,690,989,700 | 99.79 | 97.24 | 43.16 | 21.2201 |
| GCH6 | 34,204,190,100 | 34,113,295,500 | 99.73 | 97.03 | 43.64 | 25.6221 |
| GCI1 | 24,132,486,000 | 23,918,619,300 | 99.11 | 95.92 | 43.74 | 17.0911 |
| GCI2 | 27,747,621,000 | 27,627,747,900 | 99.57 | 97.06 | 43.41 | 21.079 |
| GCI3 | 29,451,532,200 | 29,347,237,500 | 99.65 | 97.12 | 43.97 | 21.7086 |
| GCI4 | 29,940,382,500 | 29,864,263,500 | 99.75 | 97.13 | 43.79 | 22.3464 |
| GCI5 | 23,530,208,100 | 23,454,234,000 | 99.68 | 96.19 | 43.31 | 17.5358 |
| GCI6 | 23,481,453,900 | 23,412,441,000 | 99.71 | 96.44 | 43.50 | 17.4495 |
| GCI7 | 22,068,151,200 | 22,000,926,900 | 99.70 | 96.28 | 43.34 | 16.6203 |

**Table S16. Detailed sampling information of 66 sequenced individuals.**

| **Species** | **Name** | **Abb.** | **Origin** | **IUCN** | **No.** |
| --- | --- | --- | --- | --- | --- |
| *Garrulax courtoisi* | Blue-crowned laughingthrush | *GCO* | Wuyuan, Jaingxi | Critically Endangered, (CR) | 14 |
| *Garrulax canorus* | Chinese Hwamei | *GCA* | Nanchang, Jiangxi | Least Concerned, (LC) | 6 |
| *Garrulax chinensis* | Black-throated laughingthrush | *GCH* | Nanning, Guangxi | Least Concerned, (LC) | 6 |
| *Garrulax perspicillatus* | Masked laughingthrush | *GPE* | Nanchang, Jiangxi | Least Concerned, (LC) | 5 |
| *Garrulax sannio* | White-browed laughingthrush | *GSA* | Nanchang, Jiangxi | Least Concerned, (LC) | 11 |
| *Garrulax berthemyi* | Rusty laughingthrush | *GBE* | Ganzhou, Jiangxi | Least Concerned, (LC) | 8 |
| *Garrulax cineraceus* | Moustached laughingthrush | *GCI* | Ganzhou, Jiangxi | Least Concerned, (LC) | 7 |
| *Trochalopteron milnei* | Red-tailed laughingthrush | *TMI* | Nanning, Guangxi | Least Concerned, (LC) | 8 |
| *Cyanopica cyana* | Azure-winged magpie | *CCY* | Nanchang, Jiangxi | Least Concerned, (LC) | 1 |
| **Total** | **-** | **-** | **-** |  | **66** |

Note: **Abb.**, abbreviation; **IUCN**, International Union for Conservation of Nature; **No.**, sampled number.

**Table S17. Pair individuals with closely relationship of 66 specimens.**

| **IID1** | **IID2** | **Z0** | **Z1** | **Z2** | **PI_HAT** | **DST** | **RATIO** |
| --- | --- | --- | --- | --- | --- | --- | --- |
| GCO1 | GCO14 | 0 | 1 | 0 | 0.5 | 0.77 | 19.67 |
| GCO2 | GCO6 | 0 | 1 | 0 | 0.5 | 0.72 | 26.56 |
| GCO2 | GCO9 | 0 | 1 | 0 | 0.5 | 0.72 | 33.36 |
| GCO2 | GCO12 | 0 | 1 | 0 | 0.5 | 0.74 | 70.31 |
| GCO4 | GCO9 | 0 | 1 | 0 | 0.5 | 0.72 | 31.00 |
| GCO4 | GCO10 | 0 | 1 | 0 | 0.5 | 0.72 | 37.58 |
| GCO8 | GCO11 | 0 | 1 | 0 | 0.5 | 0.71 | 24.02 |
| GCO11 | GCO12 | 0 | 1 | 0 | 0.5 | 0.73 | 45.70 |
| GCO11 | GCO13 | 0 | 1 | 0 | 0.5 | 0.72 | 35.63 |
| TMI1 | TMI6 | 0.10 | 0.26 | 0.64 | 0.77 | 0.90 | 33.05 |
| TMI2 | TMI8 | 0.11 | 0.27 | 0.62 | 0.75 | 0.89 | 29.83 |
| TMI4 | TMI7 | 0.14 | 0.30 | 0.56 | 0.71 | 0.87 | 25.01 |
| GBE2 | GBE4 | 0.11 | 0.11 | 0.78 | 0.84 | 0.93 | 34.63 |

**Table S18. Evidence of gene flow between *Garrulax* species.**

| **Table S18a. *GCO* shows evidence of gene flow with *GSA* and *GPE.*** | | | | | | | | |
| --- | --- | --- | --- | --- | --- | --- | --- | --- |
| **P1** | **P2** | **P3** | **P4** | **D.stat** | **sd** | **Z-score** | **N_BBAA_** | **N_BABA_** |
| *GSA* | *GCH* | *GCO* | *TMI* | -0.007294 | 0.000614 | -11.88 | 16598 | 24261 |
| *GSA* | *GPE* | *GCO* | *TMI* | 0.00036 | 0.000051 | 7.095 | 4457 | 4078 |
| *GPE* | *GBE* | *GCO* | *TMI* | -0.000202 | 0.000251 | -0.805 | 13390 | 13602 |
| *GPE* | *GCH* | *GCO* | *TMI* | -0.007654 | 0.000604 | -12.67 | 16776 | 24819 |
| *GPE* | *GSA* | *GCO* | *TMI* | -0.00036 | 0.000051 | -7.095 | 4078 | 4457 |
|  |  |  |  |  |  |  |  |  |
| **Table S18b. *GCO* shows evidence of gene flow with *GCA* and *GBE*** | | | | | | | | |
| **P1** | **P2** | **P3** | **P4** | **D.stat** | **sd** | **Z-score** | **N_BBAA_** | **N_BABA_** |
| *GSA* | *GCO* | *GBE* | *TMI* | 0.006816 | 0.000668 | 10.199 | 20187 | 13025 |
| *GPE* | *GCO* | *GBE* | *TMI* | 0.006813 | 0.00067 | 10.164 | 20761 | 13602 |
| *GCO* | *GCH* | *GCA* | *TMI* | -0.000012 | 0.000132 | -0.093 | 14567 | 14580 |
|  |  |  |  |  |  |  |  |  |
| **Table S18c. Evidence of gene flow between *Garrulax* species*.*** | | | | | | | | |
| **P1** | **P2** | **P3** | **P4** | **D.stat** | **sd** | **Z-score** | **N_BBAA_** | **N_BABA_** |
| *GPE* | *GCO* | *GCI* | *TMI* | -0.000312 | 0.000209 | -1.49 | 13532 | 13859 |
| *GCO* | *GCH* | *GCI* | *TMI* | -0.000218 | 0.000115 | -1.893 | 14681 | 14909 |
| *GSA* | *GCO* | *GCI* | *TMI* | -0.000177 | 0.000196 | -0.903 | 13098 | 13284 |
| *GPE* | *GCO* | *GCH* | *TMI* | -0.008808 | 0.000633 | -13.921 | 15564 | 24819 |
| *GSA* | *GCO* | *GCH* | *TMI* | -0.008766 | 0.000643 | -13.643 | 15051 | 24261 |
| *GPE* | *GCO* | *GCA* | *TMI* | -0.000835 | 0.000163 | -5.113 | 12995 | 13872 |
| *GCO* | *GBE* | *GCI* | *TMI* | 0.000573 | 0.000169 | 3.388 | 15289 | 14687 |
| *GCO* | *GBE* | *GCA* | *TMI* | 0.00071 | 0.000126 | 5.622 | 15170 | 14424 |
| *GCO* | *GCI* | *GCA* | *TMI* | 0.00073 | 0.000181 | 4.036 | 17374 | 16606 |

**Table S19. Genetic diversity of eight *Garrulax* species based on a reference genome of white-browed laughingthrush*.***

| **Species** | **SNPs** | **INDELs** | **P_n_** | **H_e_** | **H_o_** |
| --- | --- | --- | --- | --- | --- |
| *G. canorus* | 593,342 | 293,415 | 0.12 | 0.05 | 0.04 |
| *G. chinensis* | 902,204 | 296,501 | 0.21 | 0.08 | 0.07 |
| *G. cineraceus* | 871,796 | 276,314 | 0.20 | 0.07 | 0.06 |
| *G. courtoisi* | 1,945,320 | 371,960 | 0.51 | 0.21 | 0.18 |
| *T. milnei* | 733,565 | 280,860 | 0.11 | 0.05 | 0.04 |
| *G. perspicillatus* | 494,389 | 108,173 | 0.12 | 0.05 | 0.05 |
| *G. berthemyi* | 1,034,563 | 245,541 | 0.23 | 0.08 | 0.07 |
| *G. sannio* | 725,313 | 125,222 | 0.17 | 0.06 | 0.06 |

**Table S20. Deleterious variants of blue-crowned laughingthrush and white-browed laughingthrush.**

| **Species** | **ID** | **N_SNV_** | **N_SHoD_** | **N_SHo_** | **N_SHe_** | **N_SyHoD_** | **N_SyHo_** | **N_SyHe_** | **N_MHoD_** | **N_MHo_** | **N_MHe_** |
| --- | --- | --- | --- | --- | --- | --- | --- | --- | --- | --- | --- |
| GSA | GSA1 | 1,127,170 | 133 | 246 | 165 | 3,736 | 4,514 | 4,452 | 675 | 1,023 | 877 |
| GSA | GSA2 | 1,194,458 | 143 | 235 | 200 | 3,720 | 4,339 | 5,531 | 729 | 989 | 1,097 |
| GSA | GSA3 | 1,178,327 | 136 | 241 | 192 | 3,481 | 4,628 | 5,280 | 623 | 1,077 | 1,053 |
| GSA | GSA4 | 1,188,373 | 111 | 228 | 240 | 3,336 | 4,658 | 5,564 | 533 | 1,145 | 1,133 |
| GSA | GSA5 | 1,195,896 | 105 | 192 | 282 | 3,384 | 4,500 | 5,718 | 615 | 1,069 | 1,142 |
| GSA | GSA6 | 1,182,613 | 120 | 245 | 204 | 3,556 | 4,615 | 5,321 | 615 | 1,082 | 1,084 |
| GSA | GSA7 | 1,147,184 | 121 | 214 | 215 | 3,517 | 4,664 | 4,966 | 610 | 1,114 | 969 |
| GSA | GSA8 | 1,181,577 | 49 | 157 | 350 | 2,411 | 5,804 | 5,298 | 0 | 1,687 | 1,113 |
| GSA | GSA9 | 1,165,105 | 139 | 245 | 180 | 3,919 | 4,428 | 4,867 | 794 | 957 | 942 |
| GSA | GSA10 | 1,170,612 | 114 | 233 | 212 | 3,334 | 4,553 | 5,521 | 581 | 1,077 | 1,115 |
| GSA | GSA11 | 1,172,726 | 130 | 221 | 201 | 3,518 | 4,372 | 5,382 | 665 | 944 | 1,095 |
| GCO | GCO3 | 3,010,503 | 190 | 542 | 461 | 13,656 | 24,908 | 23,569 | 2,467 | 4,675 | 4,513 |
| GCO | GCO5 | 3,045,841 | 175 | 520 | 511 | 13,663 | 24,926 | 24,058 | 2,529 | 4,680 | 4,502 |
| GCO | GCO6 | 2,795,484 | 189 | 449 | 500 | 13,924 | 20,533 | 24,186 | 2,785 | 3,446 | 4,741 |
| GCO | GCO7 | 3,003,205 | 191 | 525 | 455 | 13,746 | 24,585 | 23,117 | 2,606 | 4,520 | 4,325 |
| GCO | GCO8 | 3,018,964 | 173 | 479 | 536 | 14,330 | 20,256 | 26,980 | 2,948 | 3,393 | 5,187 |
| GCO | GCO9 | 3,012,162 | 193 | 504 | 498 | 15,184 | 21,754 | 24,544 | 3,043 | 3,752 | 4,767 |
| GCO | GCO10 | 3,069,980 | 223 | 480 | 494 | 14,927 | 21,058 | 26,754 | 3,056 | 3,567 | 5,139 |
| GCO | GCO12 | 3,171,564 | 209 | 481 | 565 | 14,520 | 19,781 | 30,481 | 2,970 | 3,208 | 6,032 |
| GCO | GCO13 | 3,139,789 | 205 | 489 | 561 | 14,169 | 19,792 | 30,451 | 2,816 | 3,317 | 6,016 |
| GCO | GCO14 | 3,138,959 | 211 | 495 | 551 | 15,825 | 22,289 | 26,343 | 3,109 | 3,818 | 5,228 |

**N_SHoD_**, homozygous strong deleterious derived variants; **N_SHo_**, homozygous strong deleterious variants; **N_SHe_**, heterozygous strong deleterious variants; **N_SyHoD_**, homozygous synonymous derived variants; **N_SyHo_**, homozygous synonymous variants; **N_SyHe_**, heterozygous synonymous variants; **N_MHoD_**, homozygous missence derived variants; **N_MHo_**, homozygous missence variants; **N_MHe_**, heterozygous missence variants.

**Table S21. The heterozygosity rate of *GCO*, *GSA*, and the other 186 species** **(excel file).**

**Table S22. The overlapping windows identified by top 1% *F*_ST_ and zHp value.**

| **Scafffold** | **Win_start** | **Win_end** | ***F*_ST_** | **zHp** | **Gene** |
| --- | --- | --- | --- | --- | --- |
| 12 | 19400001 | 19440000 | 0.55 | -4.83 | *-* |
| 18 | 1440001 | 1480000 | 0.55 | -3.17 | *GSTA2/TM14C* |
| 18 | 1800001 | 1840000 | 0.61 | -3.60 | *-* |
| 18 | 6680001 | 6720000 | 0.52 | -5.24 | *-* |
| 34 | 5480001 | 5520000 | 0.53 | -4.68 | *SIA8F* |
| 50 | 1 | 40000 | 0.52 | -3.50 | *-* |
| 65 | 38520001 | 38560000 | 0.62 | -5.33 | *-* |
| 65 | 680001 | 720000 | 0.52 | -3.90 | *MAT1* |
| 65 | 9200001 | 9240000 | 0.53 | -7.10 | *BKRB2* |
| 69 | 2360001 | 2400000 | 0.61 | -4.65 | *PPIP2* |
| 69 | 6920001 | 6960000 | 0.55 | -3.36 | *TT39B* |
| 77 | 9120001 | 9160000 | 0.58 | -3.98 | *-* |
| 77 | 12600001 | 12640000 | 0.58 | -3.19 | *-* |
| 90 | 41680001 | 41720000 | 0.54 | -3.43 | *-* |
| 90 | 8320001 | 8360000 | 0.58 | -3.66 | *-* |
| 95 | 10720001 | 10760000 | 0.52 | -4.71 | *CARTF* |
| 95 | 15080001 | 15120000 | 0.53 | -3.36 | *MTX2/HXD3* |
| 96 | 27400001 | 27440000 | 0.53 | -3.72 | *-* |
| 104 | 200001 | 240000 | 0.64 | -4.42 | *-* |
| 104 | 1040001 | 1080000 | 0.52 | -4.67 | *EDIL3* |
| 131 | 20080001 | 20120000 | 0.55 | -8.27 | *CP26A* |
| 157 | 1000001 | 1040000 | 0.53 | -3.99 | *PCGF3* |
| 166 | 1320001 | 1360000 | 0.67 | -5.46 | *-* |
| 166 | 3200001 | 3240000 | 0.62 | -4.98 | *RUSC2* |
| 166 | 6120001 | 6160000 | 0.63 | -4.47 | *-* |
| 171 | 160001 | 200000 | 0.52 | -3.18 | *-* |
| 171 | 200001 | 240000 | 0.64 | -4.60 | *-* |
| 171 | 320001 | 360000 | 0.55 | -4.59 | *-* |
| 194 | 4080001 | 4120000 | 0.53 | -4.57 | *LMX1B* |
| 194 | 4120001 | 4160000 | 0.53 | -3.17 | *-* |
| 194 | 4240001 | 4280000 | 0.53 | -3.57 | *-* |
| 194 | 4280001 | 4320000 | 0.52 | -3.24 | *-* |
| 194 | 4320001 | 4360000 | 0.53 | -3.47 | *PBX3* |
| 196 | 14880001 | 14920000 | 0.56 | -4.95 | *-* |
| 196 | 15320001 | 15360000 | 0.53 | -3.54 | *CSMD3* |
| 196 | 44800001 | 44840000 | 0.59 | -3.14 | *-* |
| 370 | 2920001 | 2960000 | 0.52 | -3.48 | *-* |
| 456 | 2720001 | 2760000 | 0.53 | -3.60 | *CI085* |
| 456 | 2800001 | 2840000 | 0.53 | -5.10 | *MCTP1* |
| 456 | 4320001 | 4360000 | 0.58 | -5.19 | *-* |
| 456 | 4720001 | 4760000 | 0.55 | -3.91 | *-* |
| 607 | 520001 | 560000 | 0.58 | -3.17 | *-* |

**Table S23. Scaffolds belong to chromosome Z and W and the depth information of *GCO* (excel file).**

**Table S24. Scaffolds belong to chromosome Z and W and the depth information of *GSA* (excel file).**

**Table S25. The 218 STRs makersof blue-crowned laughingthrush.**

| **Scaffold** | **Position** | **Repeat** | **Allele number** | **PIC** | **Gene start** | **Gene end** | **Annotated gene** |
| --- | --- | --- | --- | --- | --- | --- | --- |
| scaffold_3 | 74783 | TTTG | 6 | 0.51 | 56975 | 82383 | *-* |
| scaffold_3 | 202600 | AGGG | 10 | 0.86 | 197870 | 205621 | *-* |
| scaffold_3 | 1135269 | TTTA | 5 | 0.70 | 1132412 | 1136676 | *-* |
| scaffold_5 | 2476632 | ACAA | 7 | 0.79 | 2475251 | 2503318 | *-* |
| scaffold_5 | 2501243 | AGAA | 19 | 0.94 | 2475251 | 2503318 | *-* |
| scaffold_6 | 897310 | GTTT | 5 | 0.64 | 866615 | 919587 | *-* |
| scaffold_6 | 4973594 | TAAA | 6 | 0.73 | 4967053 | 4998900 | *-* |
| scaffold_6 | 6997477 | AGGC | 6 | 0.68 | 6988546 | 7007657 | *-* |
| scaffold_18 | 7643198 | GTGA | 11 | 0.88 | 7626123 | 7662430 | *-* |
| scaffold_18 | 21650381 | GTTT | 5 | 0.67 | 21646740 | 21674904 | *-* |
| scaffold_35 | 326588 | TTGT | 5 | 0.67 | 275115 | 375406 | *-* |
| scaffold_42 | 176005 | TATG | 7 | 0.82 | 173668 | 192119 | *-* |
| scaffold_50 | 1854663 | AAAC | 5 | 0.37 | 1846948 | 1962566 | *-* |
| scaffold_51 | 480129 | GATA | 7 | 0.77 | 479586 | 504212 | *T55BB* |
| scaffold_51 | 1065099 | TTTG | 6 | 0.75 | 991390 | 1097888 | *-* |
| scaffold_51 | 2893932 | TATT | 6 | 0.71 | 2893139 | 2906735 | *-* |
| scaffold_51 | 4012034 | ATAT | 6 | 0.70 | 4003522 | 4014978 | *-* |
| scaffold_51 | 8508120 | TGAT | 5 | 0.69 | 8470932 | 8512183 | *-* |
| scaffold_51 | 12957403 | CGGG | 5 | 0.46 | 12953332 | 12979169 | *-* |
| scaffold_51 | 14139163 | GGAG | 5 | 0.55 | 14138126 | 14140755 | *-* |
| scaffold_51 | 15692883 | AGGA | 5 | 0.68 | 15686624 | 15708371 | *-* |
| scaffold_51 | 16470661 | CATC | 6 | 0.66 | 16462460 | 16479832 | *-* |
| scaffold_51 | 16954635 | GGGA | 7 | 0.84 | 16920558 | 17008782 | *-* |
| scaffold_52 | 6092422 | CCAA | 5 | 0.61 | 5977003 | 6100736 | *SBNO2* |
| scaffold_52 | 6874518 | ATCC | 6 | 0.70 | 6873715 | 6892883 | *BASI* |
| scaffold_52 | 6874636 | ATCC | 5 | 0.62 | 6873715 | 6892883 | *BASI* |
| scaffold_52 | 7967438 | AAAT | 7 | 0.75 | 7951727 | 7970440 | *PX11C* |
| scaffold_52 | 9102094 | AAAC | 6 | 0.47 | 9065705 | 9111244 | *C2C4B* |
| scaffold_52 | 9285012 | GTTT | 5 | 0.59 | 9221763 | 9307254 | *-* |
| scaffold_52 | 10262517 | AAAC | 5 | 0.47 | 10182661 | 10274007 | *KCNV2* |
| scaffold_52 | 10262549 | CAAA | 6 | 0.71 | 10182661 | 10274007 | *KCNV2* |
| scaffold_52 | 11031878 | AATT | 7 | 0.74 | 11028479 | 11069055 | *RB11B* |
| scaffold_52 | 11495145 | ATAT | 5 | 0.67 | 11493700 | 11517274 | *TMPS9* |
| scaffold_58 | 10157454 | TTTG | 8 | 0.81 | 10130990 | 10186914 | *LPAR4* |
| scaffold_58 | 13592078 | AACC | 5 | 0.37 | 13587270 | 13688694 | *-* |
| scaffold_58 | 14724005 | CAAA | 6 | 0.63 | 14697370 | 14756698 | *-* |
| scaffold_58 | 16108824 | AGAG | 5 | 0.47 | 16077560 | 16195466 | *-* |
| scaffold_58 | 24100139 | GTGT | 11 | 0.89 | 24062985 | 24113494 | *-* |
| scaffold_65 | 1160471 | TCCC | 5 | 0.65 | 1147810 | 1178552 | *AHNK2* |
| scaffold_65 | 1395705 | GGAG | 5 | 0.74 | 1392647 | 1419128 | *-* |
| scaffold_65 | 1638899 | TTAT | 14 | 0.90 | 1624784 | 1649530 | *-* |
| scaffold_69 | 776909 | AAAC | 5 | 0.59 | 755457 | 804824 | *-* |
| scaffold_83 | 103904 | CCAT | 5 | 0.77 | 32223 | 120908 | *-* |
| scaffold_86 | 1162394 | TTTC | 20 | 0.94 | 1159500 | 1177000 | *TF3C5* |
| scaffold_92 | 470866 | ATAA | 5 | 0.73 | 430799 | 471696 | *SNTA1* |
| scaffold_92 | 542100 | CCCA | 7 | 0.80 | 530507 | 544873 | *-* |
| scaffold_92 | 542117 | CCAT | 7 | 0.82 | 530507 | 544873 | *-* |
| scaffold_92 | 573914 | ATGG | 6 | 0.80 | 571281 | 575368 | *-* |
| scaffold_94 | 294164 | TTCG | 9 | 0.82 | 283285 | 312471 | *-* |
| scaffold_94 | 580033 | AAAC | 5 | 0.51 | 559542 | 589561 | *-* |
| scaffold_97 | 43268 | TTTG | 6 | 0.78 | 32006 | 66703 | *STIM1* |
| scaffold_98 | 1414382 | GGGA | 5 | 0.67 | 1360057 | 1425848 | *TBCC1* |
| scaffold_99 | 997362 | GTTT | 5 | 0.54 | 996183 | 1035380 | *-* |
| scaffold_99 | 2201791 | TGTT | 5 | 0.47 | 2165779 | 2236401 | *-* |
| scaffold_99 | 6225172 | TCCC | 5 | 0.62 | 6206675 | 6225339 | *-* |
| scaffold_104 | 374535 | TTTA | 5 | 0.64 | 369319 | 473540 | *-* |
| scaffold_104 | 2263734 | AAAC | 5 | 0.65 | 2190145 | 2266089 | *RS23* |
| scaffold_104 | 11068345 | GTGT | 9 | 0.80 | 11024717 | 11083075 | *-* |
| scaffold_108 | 1728396 | TTTC | 23 | 0.94 | 1713489 | 1790783 | *-* |
| scaffold_111 | 329063 | TGTG | 5 | 0.75 | 319439 | 357117 | *ACAP1* |
| scaffold_115 | 154309 | CAAA | 6 | 0.68 | 153815 | 154580 | *FBN2* |
| scaffold_118 | 2963641 | ATTC | 8 | 0.52 | 2955038 | 2964350 | *-* |
| scaffold_122 | 3770830 | TATT | 6 | 0.47 | 3762523 | 3784533 | *-* |
| scaffold_123 | 1107194 | GGAG | 5 | 0.66 | 1105882 | 1112289 | *CBS* |
| scaffold_123 | 1477167 | CTTT | 18 | 0.92 | 1458547 | 1502152 | *MUC1* |
| scaffold_123 | 2343558 | GATG | 6 | 0.68 | 2333015 | 2358382 | *PSMG1* |
| scaffold_124 | 207376 | GGGA | 7 | 0.78 | 204086 | 221274 | *MSH5* |
| scaffold_124 | 324876 | GGGA | 5 | 0.73 | 322234 | 329688 | *-* |
| scaffold_128 | 69547 | GGAT | 6 | 0.79 | 46174 | 85714 | *-* |
| scaffold_131 | 99764 | TAGA | 9 | 0.80 | 64210 | 187332 | *KLRBC* |
| scaffold_139 | 4173015 | CATA | 5 | 0.64 | 4172385 | 4213454 | *-* |
| scaffold_145 | 257410 | GGAG | 9 | 0.82 | 244007 | 272359 | *-* |
| scaffold_148 | 331399 | AGGG | 6 | 0.76 | 325091 | 340877 | *GTR1* |
| scaffold_158 | 458297 | TCTC | 5 | 0.73 | 454876 | 460323 | *-* |
| scaffold_163 | 416260 | CTCC | 5 | 0.66 | 412405 | 417507 | *MK04* |
| scaffold_165 | 222423 | TTTG | 5 | 0.42 | 191532 | 232581 | *-* |
| scaffold_178 | 1068784 | TTGT | 5 | 0.65 | 1031096 | 1092493 | *RHG12* |
| scaffold_178 | 1566248 | CACA | 8 | 0.78 | 1498346 | 1632651 | *-* |
| scaffold_178 | 1812413 | TATG | 7 | 0.56 | 1717263 | 1813259 | *CXD4* |
| scaffold_179 | 248689 | TCCC | 7 | 0.55 | 243130 | 256111 | *-* |
| scaffold_179 | 248842 | TCCC | 7 | 0.74 | 243130 | 256111 | *-* |
| scaffold_182 | 335053 | GGGA | 5 | 0.47 | 224311 | 339142 | *-* |
| scaffold_182 | 929128 | TTTA | 5 | 0.73 | 917466 | 933068 | *-* |
| scaffold_183 | 127632 | TCCA | 6 | 0.56 | 125426 | 137532 | *-* |
| scaffold_186 | 230521 | AGGA | 7 | 0.73 | 227048 | 244774 | *-* |
| scaffold_198 | 2530626 | TTTA | 6 | 0.76 | 2488856 | 2538882 | *TBX6L* |
| scaffold_198 | 2957924 | AAAG | 6 | 0.66 | 2939403 | 2965852 | *BCR* |
| scaffold_204 | 11823668 | TCTG | 7 | 0.80 | 11819010 | 11834172 | *-* |
| scaffold_206 | 13784516 | ATCT | 6 | 0.71 | 13732383 | 13842774 | *-* |
| scaffold_206 | 19411544 | AACA | 6 | 0.78 | 19311363 | 19441597 | *-* |
| scaffold_206 | 19842936 | AACC | 7 | 0.82 | 19802700 | 19844217 | *-* |
| scaffold_206 | 21462939 | CCTT | 8 | 0.77 | 21450173 | 21463283 | *-* |
| scaffold_206 | 22470005 | GGAG | 5 | 0.70 | 22463312 | 22474662 | *-* |
| scaffold_206 | 29986939 | CAAA | 7 | 0.73 | 29925790 | 29995262 | *-* |
| scaffold_206 | 33962750 | TTCT | 5 | 0.70 | 33962077 | 34038047 | *-* |
| scaffold_206 | 38198915 | ATAC | 5 | 0.58 | 38162450 | 38294849 | *-* |
| scaffold_206 | 39079490 | AGAG | 5 | 0.42 | 38980221 | 39097387 | *-* |
| scaffold_206 | 39641926 | AACA | 5 | 0.65 | 39615831 | 39668804 | *-* |
| scaffold_212 | 189598 | CCAT | 5 | 0.63 | 118809 | 207320 | *-* |
| scaffold_213 | 2466642 | CCCT | 6 | 0.52 | 2406828 | 2468939 | *-* |
| scaffold_213 | 3719550 | TGTC | 5 | 0.61 | 3710512 | 3765322 | *-* |
| scaffold_213 | 3728521 | GGAC | 6 | 0.73 | 3710512 | 3765322 | *-* |
| scaffold_216 | 2187700 | TCCA | 7 | 0.76 | 2171005 | 2193029 | *-* |
| scaffold_228 | 222702 | CCAT | 5 | 0.74 | 221037 | 224486 | *-* |
| scaffold_236 | 396613 | TCCC | 6 | 0.67 | 360466 | 411907 | *-* |
| scaffold_236 | 2167817 | TGTC | 6 | 0.69 | 2164827 | 2184288 | *-* |
| scaffold_236 | 2167873 | TGTC | 5 | 0.57 | 2164827 | 2184288 | *-* |
| scaffold_236 | 2208100 | AGAG | 7 | 0.73 | 2202984 | 2234420 | *-* |
| scaffold_237 | 6592244 | TATA | 6 | 0.70 | 6551073 | 6615833 | *-* |
| scaffold_237 | 17046139 | AAAC | 7 | 0.80 | 17011196 | 17057267 | *-* |
| scaffold_242 | 256742 | TCTG | 6 | 0.74 | 254101 | 259185 | *-* |
| scaffold_246 | 628301 | AGAC | 7 | 0.64 | 622280 | 635664 | *-* |
| scaffold_250 | 83286 | TCTT | 21 | 0.93 | 81505 | 87715 | *-* |
| scaffold_255 | 52373 | AATC | 6 | 0.78 | 32921 | 82891 | *-* |
| scaffold_255 | 3283426 | AAAC | 6 | 0.52 | 3257655 | 3301359 | *-* |
| scaffold_255 | 4220983 | TTTC | 22 | 0.94 | 4155404 | 4243751 | *-* |
| scaffold_255 | 7866448 | TATC | 11 | 0.86 | 7849586 | 7867809 | *-* |
| scaffold_255 | 12399489 | TTTG | 5 | 0.69 | 12390771 | 12419922 | *-* |
| scaffold_255 | 17126712 | TATG | 5 | 0.32 | 17124266 | 17131574 | *-* |
| scaffold_255 | 22983861 | GTTT | 5 | 0.47 | 22948697 | 22995641 | *-* |
| scaffold_255 | 26664920 | AACA | 6 | 0.67 | 26657967 | 26667384 | *-* |
| scaffold_255 | 34179091 | GGGC | 5 | 0.76 | 34178912 | 34188801 | *-* |
| scaffold_255 | 35713009 | GTTT | 8 | 0.83 | 35711100 | 35725204 | *-* |
| scaffold_256 | 5700631 | CTAC | 6 | 0.63 | 5688859 | 5723873 | *-* |
| scaffold_256 | 10178596 | TGAG | 5 | 0.66 | 10112396 | 10195219 | *-* |
| scaffold_256 | 10546634 | TATC | 8 | 0.83 | 10540284 | 10547410 | *-* |
| scaffold_256 | 11431473 | AACA | 5 | 0.67 | 11400619 | 11445962 | *-* |
| scaffold_264 | 878381 | CACA | 6 | 0.80 | 851049 | 879511 | *-* |
| scaffold_266 | 2556755 | CATA | 5 | 0.55 | 2546276 | 2561173 | *-* |
| scaffold_268 | 761555 | CCTC | 5 | 0.64 | 760981 | 776016 | *-* |
| scaffold_268 | 6624139 | CAAA | 8 | 0.70 | 6604524 | 6658621 | *-* |
| scaffold_269 | 1277919 | GTGG | 5 | 0.50 | 1264117 | 1279714 | *-* |
| scaffold_269 | 1377209 | AAAC | 5 | 0.37 | 1363951 | 1383265 | *-* |
| scaffold_281 | 12091007 | TTTG | 5 | 0.70 | 12063514 | 12106215 | *-* |
| scaffold_282 | 111372 | GACA | 5 | 0.59 | 110214 | 114328 | *-* |
| scaffold_287 | 1003047 | AAAT | 11 | 0.85 | 938926 | 1088698 | *-* |
| scaffold_287 | 4430533 | AGGG | 6 | 0.64 | 4397923 | 4446287 | *-* |
| scaffold_293 | 1216102 | TGTG | 5 | 0.58 | 1213751 | 1279846 | *-* |
| scaffold_299 | 5103819 | AGAG | 9 | 0.81 | 5063378 | 5106759 | *-* |
| scaffold_299 | 7454099 | GGAT | 6 | 0.75 | 7412102 | 7540207 | *-* |
| scaffold_303 | 63695 | TCCC | 6 | 0.66 | 62252 | 64660 | *-* |
| scaffold_303 | 63873 | CCCT | 5 | 0.70 | 62252 | 64660 | *-* |
| scaffold_304 | 520204 | CCCT | 5 | 0.42 | 516745 | 549315 | *-* |
| scaffold_304 | 902547 | AGGG | 5 | 0.37 | 900857 | 904869 | *-* |
| scaffold_307 | 2673983 | AGGG | 5 | 0.67 | 2673937 | 2681205 | *-* |
| scaffold_307 | 2674001 | GGAA | 5 | 0.71 | 2673937 | 2681205 | *-* |
| scaffold_307 | 5024618 | GATG | 6 | 0.79 | 5020879 | 5028650 | *-* |
| scaffold_315 | 3768919 | ATAC | 6 | 0.55 | 3757538 | 3779641 | *-* |
| scaffold_335 | 7384980 | TTTG | 5 | 0.72 | 7261444 | 7386538 | *-* |
| scaffold_336 | 95712 | GGGT | 6 | 0.79 | 89657 | 137913 | *-* |
| scaffold_336 | 143029 | GACA | 10 | 0.87 | 140027 | 149872 | *ACAD9* |
| scaffold_338 | 245331 | AGAG | 6 | 0.72 | 242329 | 245916 | *-* |
| scaffold_340 | 1959458 | ATAA | 5 | 0.69 | 1945919 | 1962727 | *-* |
| scaffold_346 | 740422 | GAAG | 6 | 0.70 | 721802 | 919192 | *-* |
| scaffold_347 | 2783394 | AACA | 5 | 0.64 | 2753996 | 2835133 | *-* |
| scaffold_347 | 2815335 | AAAT | 6 | 0.59 | 2753996 | 2835133 | *-* |
| scaffold_347 | 3361643 | GAAG | 5 | 0.67 | 3333929 | 3370044 | *-* |
| scaffold_352 | 12047252 | TATG | 8 | 0.80 | 12027290 | 12053648 | *-* |
| scaffold_356 | 2382548 | TGTA | 5 | 0.70 | 2349160 | 2383702 | *-* |
| scaffold_356 | 12081341 | CCCT | 5 | 0.67 | 12076615 | 12113463 | *-* |
| scaffold_356 | 12611854 | TATA | 7 | 0.55 | 12557863 | 12613439 | *-* |
| scaffold_360 | 1760371 | TGTT | 6 | 0.60 | 1620326 | 1809522 | *-* |
| scaffold_360 | 2411403 | CAAA | 7 | 0.79 | 2356728 | 2452568 | *-* |
| scaffold_360 | 30033192 | TCTT | 17 | 0.91 | 30033182 | 30033829 | *-* |
| scaffold_361 | 5819665 | GTTT | 5 | 0.64 | 5785276 | 5859855 | *-* |
| scaffold_362 | 448458 | GAGG | 5 | 0.69 | 447327 | 450157 | *-* |
| scaffold_367 | 1567813 | CCAT | 7 | 0.68 | 1484665 | 1578895 | *-* |
| scaffold_368 | 1979227 | GGGA | 8 | 0.86 | 1978771 | 1992769 | *-* |
| scaffold_376 | 8957075 | AACA | 5 | 0.55 | 8890871 | 9010336 | *-* |
| scaffold_377 | 601463 | AACC | 5 | 0.68 | 578682 | 695911 | *-* |
| scaffold_379 | 372764 | TTTA | 6 | 0.76 | 363704 | 376844 | *-* |
| scaffold_379 | 575505 | TTTC | 22 | 0.95 | 573335 | 577918 | *-* |
| scaffold_380 | 7251150 | TGTG | 8 | 0.78 | 7201252 | 7286624 | *-* |
| scaffold_380 | 15557116 | AACC | 11 | 0.86 | 15510586 | 15630174 | *-* |
| scaffold_380 | 20443273 | GTCT | 7 | 0.82 | 20440741 | 20477751 | *-* |
| scaffold_384 | 508738 | TATC | 6 | 0.47 | 490940 | 524311 | *-* |
| scaffold_386 | 14876525 | CTGT | 5 | 0.58 | 14867201 | 14896871 | *-* |
| scaffold_411 | 2201169 | TCCA | 7 | 0.81 | 2198104 | 2221496 | *-* |
| scaffold_424 | 486816 | GTGT | 6 | 0.75 | 473142 | 490939 | *-* |
| scaffold_470 | 32191 | GGAG | 5 | 0.76 | 2259 | 171222 | *AHNK* |
| scaffold_470 | 196004 | GAAA | 22 | 0.94 | 191227 | 283473 | *AHNK* |
| scaffold_491 | 111516 | GTCT | 5 | 0.71 | 110818 | 112296 | *-* |
| scaffold_509 | 5618487 | ACAT | 6 | 0.52 | 5588886 | 5618765 | *-* |
| scaffold_564 | 466468 | TCCA | 6 | 0.74 | 379347 | 471494 | *-* |
| scaffold_564 | 3578879 | CAAA | 7 | 0.76 | 3575683 | 3597892 | *-* |
| scaffold_564 | 4734617 | TGTC | 5 | 0.77 | 4686367 | 4803201 | *-* |
| scaffold_564 | 4769472 | CTGT | 6 | 0.74 | 4686367 | 4803201 | *-* |
| scaffold_678 | 50196 | TGGA | 7 | 0.72 | 38369 | 59778 | *BOP1* |
| scaffold_750 | 163824 | AAAC | 6 | 0.68 | 121295 | 178902 | *-* |
| scaffold_750 | 544275 | TGGA | 15 | 0.90 | 540025 | 607562 | *-* |
| scaffold_768 | 33543 | GGGA | 5 | 0.62 | 30441 | 41807 | *-* |
| scaffold_768 | 33852 | GGGA | 6 | 0.51 | 30441 | 41807 | *-* |
| scaffold_847 | 1004766 | GAGG | 9 | 0.84 | 946464 | 1036218 | *-* |
| scaffold_891 | 288341 | AAAT | 9 | 0.87 | 194672 | 318390 | *-* |
| scaffold_901 | 5647020 | AAAC | 6 | 0.56 | 5637930 | 5667945 | *-* |
| scaffold_1150 | 2274758 | TCCC | 6 | 0.71 | 2273115 | 2283852 | *-* |
| scaffold_1150 | 3072946 | TTTC | 8 | 0.77 | 3063261 | 3117454 | *-* |
| scaffold_1150 | 4533913 | TATC | 7 | 0.79 | 4409295 | 4643985 | *-* |
| scaffold_1150 | 6151131 | AGGG | 7 | 0.67 | 6149630 | 6170480 | *-* |
| scaffold_1198 | 1354172 | GTTA | 6 | 0.66 | 1347808 | 1355504 | *-* |
| scaffold_1198 | 1354240 | GTTT | 5 | 0.65 | 1347808 | 1355504 | *-* |
| scaffold_1237 | 5483021 | AAAC | 5 | 0.79 | 5482846 | 5490874 | *-* |
| scaffold_1237 | 6172766 | GGGA | 5 | 0.65 | 6166171 | 6194918 | *-* |
| scaffold_1366 | 23412 | TGTC | 6 | 0.73 | 11562 | 69357 | *-* |
| scaffold_1396 | 6564292 | ATAT | 8 | 0.78 | 6516426 | 6571398 | *-* |
| scaffold_1396 | 19496904 | TTGT | 5 | 0.47 | 19446093 | 19591579 | *-* |
| scaffold_1396 | 21178890 | AACC | 7 | 0.64 | 21166701 | 21233773 | *-* |
| scaffold_1396 | 26948913 | AAAC | 5 | 0.61 | 26879735 | 26974516 | *-* |
| scaffold_1439 | 1022903 | TCTG | 6 | 0.63 | 1000473 | 1076236 | *-* |
| scaffold_1552 | 472539 | TGTT | 5 | 0.58 | 442153 | 559497 | *-* |
| scaffold_1552 | 5732692 | TGTG | 5 | 0.65 | 5701709 | 5766544 | *-* |
| scaffold_1578 | 12862664 | ATTT | 5 | 0.53 | 12862109 | 12864665 | *-* |
| scaffold_1584 | 40392 | GGAG | 7 | 0.81 | 37931 | 41512 | *-* |
| scaffold_1590 | 521526 | TCTC | 7 | 0.81 | 515818 | 624112 | *-* |
| scaffold_1591 | 652955 | GGAT | 6 | 0.74 | 589731 | 683507 | *-* |
| scaffold_1591 | 1324938 | CTTC | 15 | 0.91 | 1319776 | 1337519 | *-* |
| scaffold_1603 | 946976 | TGTG | 7 | 0.78 | 931250 | 967350 | *-* |
| scaffold_1608 | 412855 | AATT | 5 | 0.71 | 409669 | 431996 | *-* |

**Table S26. The 43 STRs were identified on W-related scaffolds to be used as sex confirmation.**

| **Scaffold** | **Position** | **Repeat unit** | **Gene** |
| --- | --- | --- | --- |
| scaffold_61 | 102151 | ATA | *NFASC* |
| scaffold_61 | 102508 | AAACCC |  |
| scaffold_61 | 106197 | AAACC |  |
| scaffold_61 | 114472 | TTTATA |  |
| scaffold_61 | 127857 | AAGGAA | *CNTN2* |
| scaffold_61 | 136993 | GGAGTT |  |
| scaffold_61 | 477223 | CAAAGA | *TMCC2* |
| scaffold_61 | 1458867 | ATATGC | *MFSD4* |
| scaffold_61 | 1460488 | TTTTTA |  |
| scaffold_61 | 1463897 | CAGGA |  |
| scaffold_61 | 1492354 | GGATAG |  |
| scaffold_61 | 1492388 | AAG |  |
| scaffold_61 | 1496289 | CCCCCT |  |
| scaffold_61 | 1503122 | CAGGAA |  |
| scaffold_61 | 1507977 | AGAAAG |  |
| scaffold_61 | 2121020 | GCTGCC | S45A3 |
| scaffold_61 | 2379894 | CAAAAA | *NUCKS* |
| scaffold_61 | 2395422 | GGCCC |  |
| scaffold_61 | 2400203 | AAAACC |  |
| scaffold_61 | 2424657 | AAGCA |  |
| scaffold_61 | 2430797 | TTCT |  |
| scaffold_61 | 2455027 | TTGGGT |  |
| scaffold_61 | 3322641 | TCA | *CATE* |
| scaffold_61 | 3832308 | TAA | *IKKE* |
| scaffold_61 | 3833305 | TAAATT |  |
| scaffold_61 | 3905493 | GAT |  |
| scaffold_61 | 3922565 | ATATTT |  |
| scaffold_61 | 4196604 | TTTTTG | *EIF2D* |
| scaffold_61 | 4202830 | ATTT |  |
| scaffold_61 | 4207373 | AAAAAAAG |  |
| scaffold_61 | 4207374 | AAAAAAG |  |
| scaffold_61 | 4207375 | AAAAAG |  |
| scaffold_61 | 4212091 | GTTTTT |  |
| scaffold_61 | 4402123 | TATGGT | *DYRK3* |
| scaffold_61 | 4409447 | TGTACA |  |
| scaffold_61 | 4416363 | TAT |  |
| scaffold_61 | 4439548 | TAAGAT |  |
| scaffold_61 | 4439594 | AAGTT |  |
| scaffold_61 | 5241469 | CAATGA | *PIGR* |
| scaffold_61 | 5379711 | AGAAA | *OTU1* |
| scaffold_61 | 5390045 | TGTGCA |  |
| scaffold_61 | 5407847 | TGTTGC |  |
| scaffold_61 | 5409885 | CTATT |  |

**Table S27. The Z and W chromosomes of 31** **avian.**

| **Scientific name** | **Assembly ID** | **Z_Chromosome_ID** | **W_Chromosome_ID** |
| --- | --- | --- | --- |
| Acrocephalus scirpaceus scirpaceus | GCA_910950805.1 | OU383776.1 | OU383795.1 |
| Agelaius phoeniceus | GCA_020745825.1 | CM036750.1 | CM036749.1 |
| Camarhynchus parvulus | GCF_901933205.1 | LR595700.1 |  |
| Catharus ustulatus | GCF_009819885.2 | CM020376.2 | CM020377.2 |
| Certhia Americana | GCA_018697195.1 | CM031878.1 |  |
| Chiroxiphia lanceolata | GCF_009829145.1 | CM020566.1 | CM020567.1 |
| Coloeus monedula | GCA_013407035.1 | CM023938.1 |  |
| Corvus cornix cornix | GCA_000738735.6 | CM022210.2 |  |
| Corvus hawaiiensis | GCA_020740725.1 | CM036388.1 |  |
| Corvus moneduloides | GCF_009650955.1 | CM018841.1 | CM018842.1 |
| Diglossa brunneiventris | GCA_019023105.1 | CM032382.1 |  |
| Erithacus rubecula | GCA_903797595.2 | LR812112.1 | LR812130.2 |
| Ficedula albicollis | GCF_000247815.1 | CM002017.1 |  |
| Fringilla coelebs coelebs | GCA_015532645.2 | CM032176.1 |  |
| Geothlypis trichas | GCA_009764595.1 | CM019933.1 | CM019934.1 |
| Hirundo rustica | GCA_015227805.3 |  |  |
| Lamprotornis superbus | GCA_015883425.2 | CM040335.1 | CM040334.1 |
| Lichenostomus cassidix | GCA_008360975.2 | CM039211.1 | CM039210.1 |
| Lonchura striata domestica | GCF_005870125.1 | CM016791.1 |  |
| Molothrus ater | GCF_012460135.1 | CM022871.1 | CM022870.1 |
| Malurus cyaneus samueli | GCA_009741485.1 | CM019236.1 |  |
| Motacilla alba alba | GCF_015832195.1 | CM027504.1 |  |
| Parus major | GCF_001522545.3 |  |  |
| Passer domesticus | GCA_001700915.1 | CM004527.1 |  |
| Poecile atricapillus | GCA_011421415.1 | CM022174.1 | CM020499.1 |
| Prinia subflava | GCA_021018805.1 |  |  |
| Rhegmatorhina hoffmannsi | GCA_013398505.2 | CM031079.1 | CM031078.1 |
| Setophaga coronata coronata | GCA_001746935.2 | CM027535.1 |  |
| Sylvia atricapilla | GCA_009819655.1 | CM020498.1 |  |
| Sylvia borin | GCA_014839755.1 | CM025993.1 |  |
| Taeniopygia guttata | GCF_003957565.2 | CM012113.2 | CM018260.2 |

# References

1. Ruan J, Li H: Fast and accurate long-read assembly with wtdbg2. *Nat Methods* 2020, 17(2):155-158.

2. Li H, Durbin R: Fast and accurate short read alignment with Burrows-Wheeler transform. *Bioinformatics* 2009, 25(14):1754-1760.

3. Walker BJ, Abeel T, Shea T, Priest M, Abouelliel A, Sakthikumar S *et al*: Pilon: an integrated tool for comprehensive microbial variant detection and genome assembly improvement. *PLoS One* 2014, 9(11):e112963.

4. Parra G, Bradnam K, Korf I: CEGMA: a pipeline to accurately annotate core genes in eukaryotic genomes. *Bioinformatics* 2007, 23(9):1061-1067.

5. Simao FA, Waterhouse RM, Ioannidis P, Kriventseva EV, Zdobnov EM: BUSCO: assessing genome assembly and annotation completeness with single-copy orthologs. *Bioinformatics* 2015, 31(19):3210-3212.

6. Altschul SF, Boguski MS, Gish W, Wootton JC: Issues in searching molecular sequence databases. *Nat Genet* 1994, 6(2):119-129.

7. Birney E, Clamp M, Durbin R: GeneWise and Genomewise. *Genome Res* 2004, 14(5):988-995.

8. Stanke M, Steinkamp R, Waack S, Morgenstern B: AUGUSTUS: a web server for gene finding in eukaryotes. *Nucleic Acids Res* 2004, 32(Web Server issue):W309-312.

9. Meng X, Ji Y: Modern Computational Techniques for the HMMER Sequence Analysis. *ISRN Bioinform* 2013, 2013:252183.

10. Majoros WH, Pertea M, Salzberg SL: TigrScan and GlimmerHMM: two open source ab initio eukaryotic gene-finders. *Bioinformatics* 2004, 20(16):2878-2879.

11. Leskovec J, Sosic R: SNAP: A General Purpose Network Analysis and Graph Mining Library. *ACM Trans Intell Syst Technol* 2016, 8(1).

12. Trapnell C, Pachter L, Salzberg SL: TopHat: discovering splice junctions with RNA-Seq. *Bioinformatics* 2009, 25(9):1105-1111.

13. Trapnell C, Roberts A, Goff L, Pertea G, Kim D, Kelley DR *et al*: Differential gene and transcript expression analysis of RNA-seq experiments with TopHat and Cufflinks. *Nat Protoc* 2012, 7(3):562-578.

14. Jones P, Binns D, Chang HY, Fraser M, Li W, McAnulla C *et al*: InterProScan 5: genome-scale protein function classification. *Bioinformatics* 2014, 30(9):1236-1240.

15. Chan PP, Lowe TM: tRNAscan-SE: Searching for tRNA Genes in Genomic Sequences. *Methods Mol Biol* 2019, 1962:1-14.

16. Nawrocki EP, Eddy SR: Infernal 1.1: 100-fold faster RNA homology searches. *Bioinformatics* 2013, 29(22):2933-2935.
